# Fibrous hydrogels under biaxial confinement

**DOI:** 10.1038/s41467-022-30980-7

**Published:** 2022-06-07

**Authors:** Yang Li, Yunfeng Li, Elisabeth Prince, Jeffrey I. Weitz, Sergey Panyukov, Arun Ramachandran, Michael Rubinstein, Eugenia Kumacheva

**Affiliations:** 1 Department of Chemical Engineering & Applied Chemistry, University of Toronto, 200 College Street, Toronto, ON M5S 3E5 Canada; 2 Department of Chemistry, University of Toronto, 80 Saint George Street, Toronto, ON M5S 3H6 Canada; 3 Thrombosis and Atherosclerosis Research Institute, 237 Barton Street East, Hamilton, L8L 2 × 2 ON Canada; 4 Department of Biochemistry and Biomedical Sciences, McMaster University, 1280 Main Street West, Hamilton, ON L8S 4K1 Canada; 5 Department of Medicine, McMaster University, 1200 Main Street West, Hamilton, ON L8N 3Z5 Canada; 6 P. N. Lebedev Physics Institute, Russian Academy of Sciences, 53 Leninskiy Prospekt, Moscow, 119991 Russian Federation; 7 Department of Mechanical Engineering and Materials Science, Duke University, Durham, NC 27708 USA; 8 Department of Biomedical Engineering, Duke University, Durham, NC 27708 USA; 9 Department of Chemistry, Duke University, Durham, NC 27708 USA; 10 Department of Physics, Duke University, Durham, NC 27708 USA; 11 World Primer Institute for Chemical Reaction Design and Discovery (WPI-ICReDD), Hokkaido University, Sapporo, Hokkaido 001-0021 Japan; 12 Institute of Biomedical Engineering, University of Toronto, 164 College Street, Toronto, ON M5S 3G9 Canada; 13 Present Address: Department of Orthopaedics, University Medical Center Utrecht, Utrecht University, Heidelberglaan 100, 3584 CX Utrecht, The Netherlands; 14 Present Address: State Key Laboratory of Supramolecular Structure and Materials, College of Chemistry, Jilin University, 2699 Qianjin Street, Changchun, 130012 China; 15 Present Address: Department of Chemistry, Massachusetts Institute of Technology, 88 Ames Street, Apartment 306, Cambridge, MA 02142 USA

**Keywords:** Soft materials, Condensed-matter physics

## Abstract

Confinement of fibrous hydrogels in narrow capillaries is of great importance in biological and biomedical systems. Stretching and uniaxial compression of fibrous hydrogels have been extensively studied; however, their response to biaxial confinement in capillaries remains unexplored. Here, we show experimentally and theoretically that due to the asymmetry in the mechanical properties of the constituent filaments that are soft upon compression and stiff upon extension, filamentous gels respond to confinement in a qualitatively different manner than flexible-strand gels. Under strong confinement, fibrous gels exhibit a weak elongation and an asymptotic decrease to zero of their biaxial Poisson’s ratio, which results in strong gel densification and a weak flux of liquid through the gel. These results shed light on the resistance of strained occlusive clots to lysis with therapeutic agents and stimulate the development of effective endovascular plugs from gels with fibrous structures for stopping vascular bleeding or suppressing blood supply to tumors.

## Abstract (web summary)

Studies of the confinement of filamentous hydrogels such as fibrin, a major component of blood clots, can shed light on the resistance of strained occlusive clots to lysis with therapeutic agents. Here, the authors show the mechanism of the response of fibrous hydrogels to confinement.

## Introduction

Fibrous networks are a major structural and functional component of tissue and living cells. Actin is the main element of the cytoskeleton^1^; fibrin is a crucial element of wound healing and thrombosis^2^, and collagen, elastin, and fibronectin are the constituents of the extracellular matrix in the animal kingdom^3^. Reconstituted networks of fibrous biopolymers have emerged as materials with a broad range of applications in tissue engineering^4^.

Filamentous networks represent a distinct class of biological soft matter, with mechanical properties that are distinct from those of networks of flexible molecules^5^. Some of these properties have developed during the course of evolution to control the response of biological matter to deformation^6^. For example, fibrous networks show linear elasticity at small strains^7,8^, while at larger deformation, they exhibit an increase in stiffness^9,10^, thus ensuring tissue integrity. The implications of other mechanical properties of fibrous gels, e.g., the negative normal stress in response to the shear strain^11,12^ are yet to be discovered.

The mechanical properties of semiflexible fibrous hydrogels have been studied under uniaxial stretching^13,14^ and compression^8,15^, however their confinement-induced biaxial compression in narrow capillaries or tubes has not been examined. Here, we report experimental findings and propose theoretically the mechanism of the behavior of fibrous hydrogel under biaxial confinement in a microfluidic channel.

## Results

### Preparation and characterization of fibrin microgels

**Fig. 1:**
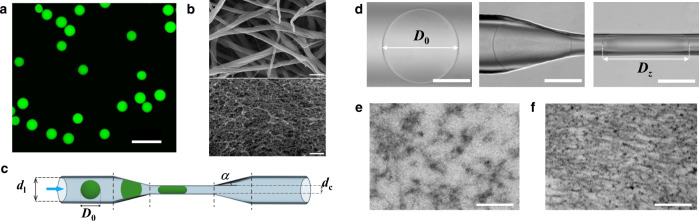
Properties of fibrin microgels. **a** Fluorescence microscopy image of fluorescein isothiocyanate (FITC)-labeled RMs suspended in TBS. The scale bar is 500 μm. **b** SEM images of the SM (top) and RM (bottom). The scale bars are 500 nm. **c** Schematic of the microfluidic channel containing a channel-at-large (diameter of *d*_l_) and a constriction with the entrance angle, *α*, of the tapered region of 15° and a diameter, *d*_c_ = 65 μm. **d** Left-to-right: optical microscopy images of RM (diameter of *D*_0_) in the channel-at-large, the tapered zone, and in the constriction (length of confined gel of *D*_z_). The scale bars are 100 μm. **e**, **f** TEM images of the undeformed RM (**e**) and occlusive RM (**f**), following its one-hour confinement in the constriction at 1/*λ*_r_ = 2.7, subsequent release, and fixing with 5 wt% solution of glutaraldehyde in TBS. The diameter of the undeformed RM was 176 μm. The scale bar is 100 nm.

Fibrin microgels with varying fibrinogen-to-thrombin concentration ratio and diameter, *D*_0_, from 150 to 220 μm were generated using a microfluidic approach (Supplementary Fig. 1). Figure 1a shows a confocal fluorescence microscopy (CFM) image of fluorescent dye-labeled microgels. The microgels had a spherical shape, polydispersity below 5%, and a uniform structure on the scale examined by CFM (Supplementary Information and Movies S1 and S2). The mean pore size of the microgels (determined by measuring the Darcy permeability^16^) reduced from 2280 to 60 nm with fibrin content increasing from 5.25 to 37.9 mg/mL and thrombin concentration reducing from 2.56 to 0.27 Unit/mL, respectively (Supplementary Figs. 2, 3 and Supplementary Table 1). The corresponding microgel stiffness increased from 0.85 to 3.6 kPa (Supplementary Fig. 4). Agarose microgels with various stiffness were used as an example of gels formed by flexible strands^17^.

We focused on fibrin microgels with the stiffness of 0.85, 1.87, and 3.6 kPa (later in the text referred to as soft microgels (SMs), medium rigidity microgels (MMs), and rigid microgels (RMs), respectively. This range of fibrin gels stiffness was on the same order of magnitude as that reported for blood clots^18,19^ and therefore, fibrin gels studied in our work have direct relevance to the real biological systems. Figure 1b, top and bottom shows a scanning electron microscopy (SEM) image of the structure of SM and RM, respectively. An SM network was formed by thicker fibers with fewer branching points, in comparison with the RM structure, in agreement with earlier reports^20,21^ (Supplementary Fig. 5). The difference in the hydrogel structure correlated with the trend in the variation in its properties: gel permeability reduced with the decreasing pore size from SMs to MMs to RMs (Supplementary Table 1), while gel stiffness changed in the opposite order. Microgel structure did not noticeably change after 30 days of storage at 4 °C (Supplementary Fig. 6).

Figure 1c shows a schematic of the microfluidic channel with a circular cross-section, which contains (from left to right): a channel-at-large with diameter *d*_l_, in which the microgel remained undeformed, a tapered region, a constriction with diameter *d*_c_ < *D*_0_, a tapered region, and a channel-at-large with diameter *d*_l_ (Supplementary Fig. 7). In a typical experiment, a microgel was introduced under a positive pressure difference Δ*P* of 0.2–16 kPa into the microfluidic channel (Supplementary Fig. 8). This range of pressures corresponds to the biologically relevant blood pressure (120 mmHg = 16 kPa)^22^. Figure 1d (from left to right) shows representative images of an RM in the channel-at-large, the tapered region, and the constriction. Microgel motion and shape were recorded and analyzed using a MATLAB program. Importantly, in the tapered region and the constriction the microgel was in conformal contact with the microchannel walls (Supplementary Fig. 8). The degree of radial microgel confinement *D*_0_/*d*_c_ = 1/*λ*_r_ in the constriction was in the range of 2.4 ≤ 1/*λ*_r_ ≤ 4.2, where 1/*λ*_r_ is the compression ratio. At Δ*P* > Δ*P*_tr_, the microgel passed the constriction, where Δ*P*_tr_ is the translocation pressure difference. The length and the size of pores of biaxially confined microgels were determined for their equilibrated state, since accounting for gel’s viscoelasticity is of utter importance in biological systems. The time of equilibration was 10 and 30 min for agarose and fibrin microgels, respectively. After these time intervals, confined microgel reached their steady-state position and shape, which were recorded with a high-speed camera and analyzed by MATLAB.

Figure 1e, f show transmission electron microscopy (TEM) images of the structure of the undeformed and biaxially confined RM. Upon RM confinement in the constriction, the size of microgel pores significantly reduced and their shape became anisotropic, with smaller dimensions in the direction of compression, in agreement with an earlier report^23^.

### Experimental and theoretical analysis of biaxially compressed microgels

**Fig. 2:**
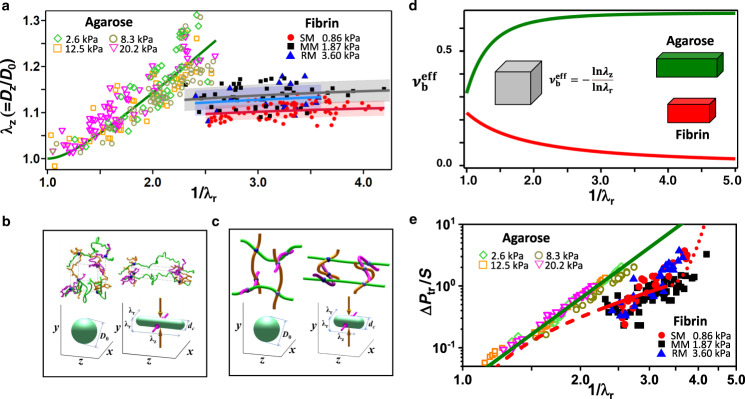
Biaxial compression of fibrin and agarose gels. **a** Variation in experimentally measured elongation *λ*_z_ of agarose microgels with various elastic moduli (2.6 kPa, green hollow diamonds; 8.3 kPa, brown hollow circles; 12.5 kPa, orange hollow squares; and 20.2 kPa, magenta hollow inverted triangles) and SM (red solid circles), MM (black solid squares), and RM (blue solid triangles). The solid lines show theoretically predicted *λ*_z_ of agarose (green line) and fibrin microgels (the colors of lines and symbols coincide). **b**, **c** Top: schematic of network strands of agarose (**b**) and fibrin (**c**) prior to (left) and after (right) biaxial compression. Bottom: the shapes of the corresponding networks before and after deformation. The compression directions x and y are shown by the magenta and brown arrows, respectively. In the top cartoons, the network strands oriented along these x and y directions are shown as the corresponding magenta and brown color lines, while the strands oriented in the unconstrained z-direction are depicted by green lines. In fibrin gel (**c**), magenta and brown strands oriented in the x and y directions are bent more than in the undeformed state, whereas the green strands oriented in the z-direction are bent and stretched. Stress between compression and stretching directions is transmitted through the filaments with intermediate orientations. In agarose gels, the strands of all orientations determine the osmotic pressure, which makes a significant contribution to gel deformation. **d** Predicted variation in biaxial Poisson’s ratio, ${\nu }_{{{{{{\rm{b}}}}}}}^{{{{{{\rm{eff}}}}}}}=-{{{{{\rm{ln}}}}}}{\lambda }_{z}/{{{{{\rm{ln}}}}}}{\lambda }_{r}$, for equi-biaxial compression of agarose (green line) and fibrin (red line) gels. Inset illustrates biaxial deformation of gels. **e** Variation in the translocation pressure, Δ*P*_tr_, normalized by the gel stiffness *S*, plotted as a function of the compression ratio for agarose and fibrin microgels. The colors of symbols correspond to those in (**a**). The green and red lines depict the theoretical relationships between Δ*P*_tr_/S and 1/*λ*_r_ for agarose and fibrin gels, respectively. The dotted fragment of the red line shows the rise in Δ*P*_tr_ at strong compression, due to inter-fiber interactions.

Biaxial compression in the constriction resulted in microgel elongation in the unconstrained direction by the factor *λ*_z_ = ${D}_{{{{{{\rm{z}}}}}}}$/${D}_{0}$, where ${D}_{{{{{{\rm{z}}}}}}}$ is the length of the confined microgel. Figure 2a shows the variation of *λ*_z_ *vs*. 1/*λ*_r_ for fibrin and agarose microgels. Surprisingly, for strong compression of 2.4 ≤ 1/*λ*_r_ ≤ 4.2, fibrin microgels exhibited an insignificant elongation *λ*_z_ of 1.12 + /−0.03, which was only weakly affected by the 1/*λ*_r_ value. Such response to biaxial confinement was in marked contrast with the behavior of confined agarose microgels, for which larger elongation *λ*_z_ = 1.3 was observed even at a weaker compression of 1/*λ*_r_ = 2.6.

This difference is due to the different mechanism of deformation of fibrin and agarose microgel networks that are composed of flexible^24^ and stiff^25^ filaments, respectively. Biaxial compression of a flexible gel leads to the decrease of its volume and the related increase of concentration and osmotic pressure that results in gel elongation in the unconstrained direction. The ultimate gel elongation is determined by the balance of entropic free energy increase of extended chains and osmotic free energy decrease due to the lower polymer concentration in the extended gel^17^. For strong biaxial compression, gel elongation increases as *λ*_z_ ≈ 0.6 ${{\lambda}_{{{\rm{r}}}}^{-2/3}}$ (*see* the green line in Fig. 2a and Supplementary Discussion Section 5.3.3). The changes in the conformation of flexible strands prior to and after biaxial confinement and of the corresponding network shapes are illustrated in Fig. 2b.

In marked contrast, filamentous gels such as fibrin respond to biaxial confinement in a qualitatively different manner. The filaments that are predominantly oriented parallel to the compression direction, bend (thus decreasing the distance between the crosslinking points), while the filaments that are mostly perpendicular to the compression direction are straightened and stretched by elastic forces, leading to gel elongation (Fig. 2c). The structure of undeformed SMs, MMs, and RMs was characterized by analyzing their SEM and CFM images (Supplementary Discussion Section IV and Supplementary Fig. 9). By determining the elastic modulus (*E*), diameter (*d*), contour length (*R*_0_), end-to-end distance (*L*_0_ ≈ *R*_0_), and central angle (*ψ*_0_) of the filaments in the undeformed fibrin microgels (Supplementary Tables 2–4), we found that the bending filament modulus, ${k}_{{{{{{\rm{b}}}}}}}=\frac{9\pi E{d}^{4}}{4{\psi }_{0}^{2}{L}_{0}}$ is significantly smaller than its stretching modulus ${k}_{{{{{{\rm{s}}}}}}}=E\frac{\pi {d}^{2}{R}_{0}}{4}$, so that *k*_b_/*k*_s_ ≈ 0.1 (Supplementary Table 4). Thus, upon biaxial confinement of a gel, fibrin filaments easily bend but resist stretching. The elongation of filamentous network subjected to biaxial compression is illustrated in Supplementary Fig. 17.

We developed a theoretical affine model (Supplementary Discussion Section V and Supplementary Figs. 10–16), in which fibrous gel elongation was determined from the local balance of elastic forces acting in the gel, and predicted strain *λ*_z_ −1 under strong biaxial confinement as

1 $${\lambda }_{{{{{{\rm{z}}}}}}}-1\simeq {\left(\frac{{k}_{{{{{{\rm{b}}}}}}}}{{k}_{{{{{{\rm{s}}}}}}}}\right)}^{1/2}\left[\frac{1}{\sqrt{3}}+\frac{\sqrt{3}}{2}{\lambda }_{{{{{{\rm{r}}}}}}}^{2}\,\ln{\lambda }_{{{{{{\rm{r}}}}}}}\right]$$

Equation (1) shows that even under strong compression (${\lambda }_{{{\mbox{r}}}}\,\to \,0$) there is a weak increase and subsequent saturation of the gel elongation strain at *λ*_z_–1 = 0.15 ± 0.05. Such behavior originates from (i) the small value of ${\left({k}_{{{{{{\rm{b}}}}}}}/{k}_{{{{{{\rm{s}}}}}}}\right)}^{1/2}$ ≈ 0.15−0.4 and (ii) the term in the square brackets asymptotically approaching $1{{\mbox{/}}}\sqrt{3}$ for strong biaxial confinement. Importantly, the prefactor ${\left({k}_{{{\mbox{b}}}}/{k}_{{{\mbox{s}}}}\right)}^{1/2}$ is independent of the filament stiffness *E* and is determined only by the filament aspect ratio *d*/*L*_0_ and central angle of an arc *ψ*_0_, which are similar for SM, MM and RM (Supplementary Table 4).

To further highlight the difference in the confinement-induced strain of flexible and filamentous gels, we introduced a biaxial Poisson’s ratio ${\nu }_{{{{{\rm{b}}}}}}{{\mbox{=}}}\,\mathop{{\lim}}\limits_{{\lambda }_{{{{{\rm{r}}}}}}\to 1}\frac{{\lambda }_{{{{{\rm{z}}}}}}-1}{1-{\lambda }_{{{{{\rm{r}}}}}}},$ which describes gel deformation in the unconstrained direction in response to equal deformations in two radial directions, and extended it to large uniform deformation ${{{{{{\rm{\nu }}}}}}}_{{{{{{\rm{b}}}}}}}^{{{{{{\rm{eff}}}}}}}=-{{{{{\rm{ln}}}}}}{\lambda }_{z}/{{{{{\rm{ln}}}}}}{\lambda }_{{{{{{\rm{r}}}}}}}$. Figure 2d shows the predicted variation of ${{{{{{\rm{\nu }}}}}}}_{{{{{{\rm{b}}}}}}}^{{{{{{\rm{eff}}}}}}}$ for the uniform biaxial compression of flexible (such as agarose) and stiff (such as fibrin) gels (Supplementary Discussion Section 5.3.4) and highlights a strong difference in their response to confinement. For an agarose gel, under strong confinement, ${{{{{{\rm{\nu }}}}}}}_{{{{{{\rm{b}}}}}}}^{{{{{{\rm{eff}}}}}}}$ increases to an asymptotic value of 2/3, while for fibrin gel it decreases to zero, as ln*λ*_z_/ln*λ*_r_ → 0 because *λ*_z_ saturates with increasing *λ*_r_. Note that in experiments, a confined spherical microgel underwent non-uniform deformation, with its central part experiencing stronger compression, however, the extrapolation to large 1/*λ*_r_ enabled comparison between experiment and theory of uniformly deformed gels.

A further difference in the behavior of flexible-strand and filamentous gels was found for their translocation through the constriction. The translocation pressure Δ*P*_tr_ normalized by gel stiffness *S*, increased with increasing compression (Fig. 2e), however for 2.0 ≤ 1/*λ*_r_ ≤ 3.5, fibrin microgels passed the constriction at substantially smaller Δ*P*_tr_/*S* values. Confinement of agarose microgels led to an increase in osmotic pressure, resulting in gel extension in the longitudinal direction with the stretching of the polymer molecules (Fig. 2b, left) and an increase in the translocation pressure as Δ*P*_tr_/*S* ∼ (1/*λ*_r_)^14/3^ ^17^. In contrast, the shape of the confined fibrin microgels was determined by the balance of the energy of filaments compressed radially and stretched in the longitudinal direction, which resulted in the maximum longitudinal strain *λ*_z_ ~$\sqrt{{k}_{{{{{{\rm{b}}}}}}}/{k}_{{{{{{\rm{s}}}}}}}}$. For 1/*λ*_r_ ≫ 1, the change in translocation pressure scaled as Δ*P*_tr_/*S* ∼${{\lambda }}_{{{{{{\rm{r}}}}}}}^{{-}1}{{{{{\rm{ln}}}}}}\left({{\lambda }}_{{{{{{\rm{r}}}}}}}^{{-}1}\right)$ (Supplementary Discussion Section 5.4), as shown by the solid red line in Fig. 2e. Thus, the dependence of Δ*P*_tr_ on confinement was weaker than for agarose gel. For compression of 1/*λ*_r_ > 3.5, a significant increase in filament volume fraction and interactions of neighboring filaments constrained further gel deformation and resulted in the deviation of experimental results from the prediction (the red dotted line in Fig. 2e). We conclude that for the same 1/*λ*_r_ and Δ${P}_{{{{{{{\rm{tr}}}}}}}_{{{{{{\rm{fibrin}}}}}}}}$ < Δ*P* < Δ${P}_{{{{{{{\rm{tr}}}}}}}_{{{{{{\rm{agarose}}}}}}}}$, the agarose gel would be trapped in the microchannel, while a fibrin gel of the same stiffness would pass it. For Δ*P* < Δ${P}_{{{{{{{\rm{tr}}}}}}}_{{{{{{\rm{fibrin}}}}}}}}$, both gels would obstruct the channel, however the fibrin gel would be pushed deeper and would experience a stronger compression, thus more effectively blocking the flow of liquid. The results shown in Fig. 2 imply that fibrous gels would act as effective plugs to reduce bleeding or suppress blood supply to the tumor.

**Fig. 3:**
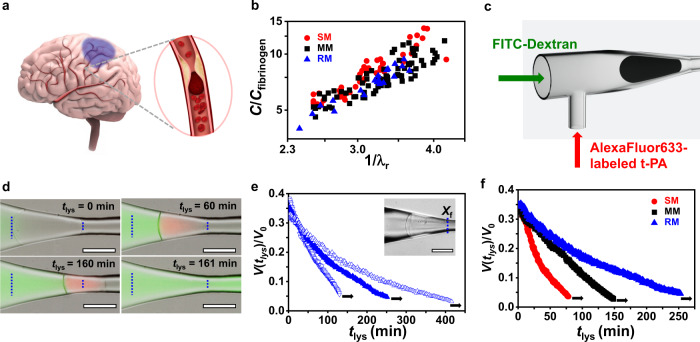
Lysis of occlusive fibrin microgels. **a** Schematic of occlusion of a cerebral artery in the brain. **b** Confinement-mediated relative increase in fibrin concentration in obstructive SMs (red solid circles), MMs (black solid squares), and RMs (blue solid triangles). **c** Experimental design used for studies of lysis of the confined fibrin gel. A solution of fluorescently labeled tPA in TBS was infused at a flow rate of 5.6 × 10^7^ μm^3^/s and an additional pressure difference of 0.7 Pa from a channel placed orthogonally to the long axis of the main microchannel. **d** Merged multi-channel microscopy images of an obstructive MM (*D*_0_ = 200 μm) positioned at *X*_f_ = 28 μm at Δ*P* = 700 Pa and subjected to digestion. Vertical dashed lines show the original positions of the back and front MM edges at *t*_lys_ = 0. Green and pink colors correspond to FITC-Dextran (70 kDa) and AlexaFluor633-labeled tPA, respectively. **e** Temporal variation in the relative volume of occlusive RM with *D*_0_ of 174 μm (blue hollow inverted triangles), 199 μm (blue triangles), and 218 μm (blue hollow triangles), respectively, placed at *X*_f_ = 28 ± 1 μm in the tapered microchannel region at Δ*P* of 1200, 1800, and 3000 Pa, respectively, and Q = 1860 ± 70 μm^3^/s. Inset shows RM (*D*_0_ = 218 μm) blocking the microchannel. **f** Temporal variation in the relative volume of SM, MM, or RM (*D*_0_ = 197 ± 3 μm) placed at *X*_f_ = 32 ± 12 μm in the tapered microchannel zone at Δ*P* of 400, 750, and 1800 Pa and Q of 12300, 2400, and 1860 μm^3^/s, respectively. *X*_f_ is the front edge position of the microgel determined its distance from the beginning of the constriction. *V*(*t*_lys_) and *V*_0_ is temporal volume of lysed microgel and the volume of the unperturbed microgel, respectively. The colors of symbols correspond to those in **b**. The black arrows in **e**, **f** correspond to the last time point before the microgel passed the microchannel. Scale bars in **d**, **e** are 100 μm.

On the other hand, fibrin forms the scaffold of blood clots that cause thromboembolism, a pathological condition in which a thrombus obstructs a blood vessel at Δ*P* < Δ*P*_tr_ in e.g., some types of ischemic strokes (Fig. 3a). A weaker confinement-induced elongation of fibrin microgels, in comparison with flexible-strand gels, leads to a stronger increase in fibrin concentration, *C/C*_fibrinogen_, where *C* and *C*_fibrinogen_ are the polymer concentrations in the confined and undeformed microgel, respectively. Figure 3b shows a more than seven-fold increase in *C/C*_fibrinogen_ at 1/*λ*_r_ ≈ 4.0 for SM, MM, and RM, driven by the confinement and the loss of water (Supplementary Fig. 16).

### Lysis of occlusive fibrin microgels

To examine the consequences of confinement on the reduction in the flux of liquid through obstructive fibrin gels, we studied lysis of SMs, MMs, and RMs that were infiltrated by with a thrombolytic agent tissue plasminogen activator (tPA). Figure 3c shows the experimental design used for lysis experiments. At Δ*P* = 700 Pa (<Δ*P*_tr_) and a flow rate, *Q* = 2400 μm^3^/s, of Tris-buffered saline (TBS) mixed with 0.1 mg/mL of (fluorescein isothiocyanate) FITC-Dextran, the microgel occluded the tapered microchannel region. The front edge position, *X*_f_, of the microgel determined its distance from the beginning of the constriction, *X*_0_. To induce lysis, a solution of fluorescently labeled tPA in TBS was infused from a channel placed orthogonally to the long axis of the main microchannel.

When the tPA solution reached the occlusive MM, the back microgel edge became blurry, indicating the initiation of fibrin digestion at time *t*_lys_ = 0 (Fig. 3d and Supplementary Fig. 18). During fibrinolysis, the dye-labeled tPA accumulated in the MM interior and bound to fibrin filaments^26^, leading to the gradually increasing intensity of the pink color of the microgel. At *t*_lys_ = 60 min, the MM shrank, due to the dissolution of its back portion, while the position of its front edge, *X*_f_, changed insignificantly. After 160 min, a strongly contracted MM moved further into the constriction, and at *t*_lys_ = 161 min, it passed through the constriction, thus restoring the flow of the liquid through the microchannel (Fig. 3d and Supplementary Fig. 18, right column).

Figure 3e shows lysis-mediated time-dependent reduction in volume *V*(*t*_lys_) normalized by the original volume, *V*_0_, of fibrin microgels with different dimensions. An RM with *D*_0_ of 174, 199, or 218 μm was placed in the microchannel at Δ*P* of 1200, 1800, or 3000 Pa, respectively, and Q = 1860 ± 70 µm^3^/s to obstruct the microchannel (Fig. 3e, inset). With a tPA supply, the microgels gradually shrank, until they became sufficiently small to pass the channel. Longer lysis time was required for the critical volume reduction for RMs with a larger original diameter. As flux through the RMs with different dimensions was similar, lysis occurred at a similar rate, resulting in the digestion of a smaller fraction of larger RMs and their delayed translocation. Figure 3f shows the lysis-driven relative decrease in *V*(*t*_lys_)/*V*_0_ for SM, MM, and RM with *D*_0_ = 197 ± 3 μm, plotted as a function of *t*_lys_. Each microgel was placed the microchannel at Δ*P* of 400, 750, or 1800 Pa, and Q of 12300, 2400, or 1860 μm^3^/s for SM, MM, and RM, respectively. Even though the pressure applied to SM was 4.5-fold lower than that for RM, the flux through SM was more than six-fold stronger, due to its higher permeability, and the rate of microgel shrinkage decreased from SM to MM to RM. For example, at *t*_lys_ = 78 min, the SM largely dissolved and translocated, while the MM and RM in spite of maintaining only 16 and 20% of their original volumes, respectively, continued to obstruct the microchannel. These results signify the importance of convection-mediated lysis of confined fibrous gels and correlate with reports on the faster digestion of clots with a lower fibrin content^27,28^.

In summary, our work shows experimentally and theoretically the mechanism of the response of filamentous gels to biaxial confinement. The behavior of fibrous gels under confinement was governed by the strong asymmetry in the deformation energy of filaments (soft upon compression and stiff upon extension) and was controlled solely by the filament aspect ratio and curvature. This response led to a minimal elongation of fibrous gels confined to narrow capillaries, a decrease in their biaxial Poisson’s ratio with increasing compression, and smaller translocation pressures, compared to flexible-strand gels with similar stiffness.

Since biaxial confinement of soft deformable particles is used in a broad range of technologies^29–32^, our findings stimulate the development of new fibrous materials. In particular, biaxial confinement of filamentous gels in narrow capillaries or tubes would result in their strong densification and a significant reduction in permeability. A strongly suppressed liquid flux through occlusive fibrous gels offers advantages in their use as plugs for preventing bleeding or reducing blood supply to malignant tumors^33–35^. On the other hand, the reduction in the liquid flux through obstructive fibrin gels and hence, suppressed convection-mediated thrombolysis provides insight into the slow lysis of occlusive blood clots^27,36,37^. Our model system is the first step toward developing understanding of the consequences of the mechanical response of fibrous biopolymer hydrogels to biaxial confinement. Incorporating blood cells or platelets in obstructive fibrin gels will affect their confinement-mediated behavior^38^ and would be the next step in uncovering the behavior of more complex biologically relevant systems.

## Methods

### Preparation of fibrin microgels

The reagents for the preparation of fibrin microgels and the fabrication of MF devices are described in the Supplementary Information (Supplementary Methods Section 2 and 4). Fibrin microgels were prepared using emulsification of the mixed solution of fibrinogen, Tris buffer and thrombin in a flow-focusing MF device, which was followed by droplet gelation. Bovine fibrinogen solution (60 mg/mL in TBS), Tris buffer and bovine thrombin solution (5 U/mL in 10 mM CaCl_2_ solution) were injected in the MF device using two independently controlled syringe pumps (PhD 200 Harvard Apparatus PHD 2000 Syringe Pump, USA). The continuous phase, F-oil containing 1 wt% of block copolymer PFPE-P(EO-PO)-PFPE, was injected into the MF device using the third syringe pump. The droplets generated in the MF device were collected in a 15 mL centrifuge tube containing F-oil. The tube was placed for 1 h in the water bath at 37 °C to complete fibrin gelation. FITC-labeled fibrin microgels were prepared from the mixture of bovine fibrinogen and FITC-labeled human fibrinogen that were mixed at the weight ratio of 33:1, respectively. The procedure was identical to that used for the preparation of fibrin microgels.

The microgels were transferred from F-oil to TBS by centrifuging the dispersion at 185 *g* for 2 min. The settled microgels were dispersed in F-oil mixed with 20 wt% of perfluorooctanol, and after that, in hexane containing 0.5 wt% of Span 80, hexane, aqueous 0.1 wt% Triton X solution, and TBS. Finally, the microgels were dispersed in TBS containing 0.01 wt% of Tween 20 and stored at 4 °C for ~1–2 weeks prior to the experiments.

### Microfluidic study of fibrin microgels

The fabrication of the MF devices is described in the Supplementary Information (Supplementary Methods Section 5). In a typical experiment, a positive Δ*P* controlled by the relative heights of water reservoirs connecting upstream and downstream to the MF device, was used to introduce a microgel with a diameter 150 < *D*_0_ < 270 μm into a microchannel. The unperturbed size of the microgel was determined by imaging it in the channel-at-large. The microgel stopped in a tapered zone at the constriction entrance. A MATLAB program was used to determine the position of the microgel along the x-axis when the front microgel tip remained invariant for 2 min. Following a step-wise increase in Δ*P*, the microgel moved along the tapered zone, until it entered the constriction. After complete microgel insertion into the constriction, Δ*P* was rapidly reduced to zero by balancing the water level between the reservoirs, and the occlusive microgel was maintained in the constriction in a stationary state. The length of the obstructive microgel was measured after its 30 min confinement in the constriction.

### Lysis of fibrin microgels

In the course of fibrinolysis experiments, t-PA and FITC-labeled dextran solutions permeated an occlusive microgel. The flow of each liquid was monitored by individual-channel fluorescence imaging. AlexaFluor 633-labeled t-PA attached to fibrin fibers and accumulated in the interior of the shrinking fibrin microgel (TRITC channel in Supplementary Fig. 18). The solution of FITC-labeled dextran moved through the microgel without accumulation.

## Supplementary information

Supplementary Information

Description of Additional Supplementary Files

Supplementary Movie 1

Supplementary Movie 2

## Data Availability

The data that support the findings of this study are available from the corresponding author upon request. The raw SEM images of fibrin gel, the raw TEM images of fibrin gel before and after confinement, and the source data underlying Figs. 2 and 3 are provided in the Source Data file. Source data are provided with this paper.

## Source data

Source Data
