# Supplementary material for: Fibrous hydrogels under biaxial confinement
Source: Nat Commun. 2022 Jun 7;13:3264. doi: 10.1038/s41467-022-30980-7 (PMC9174476; doi:10.1038/s41467-022-30980-7)
Supplement: Supplementary file 1 — Supplementary Information [file 41467_2022_30980_MOESM1_ESM.pdf]

Supplementary Information

Fibrous hydrogels under biaxial confinement

Yang Li, Yunfeng Li, Elisabeth Prince, Jeffrey I. Weitz, Sergey Panyukov, Arun Ramachandran*, Michael Rubinstein**, Eugenia Kumacheva ***

Email: *arun.ramchandran@utoronto.ca *(AR)* **mr351@duke.edu *(MR)*, ***eugenia.kumacheva@utoronto.ca *(EK)*

This file includes:

Supplementary Methods......1

1. Materials......1
2. Generation of microgels......2
3. Microgel characterization......2
4. Fabrication of microfluidic devices......5
5. Biaxial microgel compression......5

Supplementary Discussion......6

Section I. Determination of permeability and pore size of fibrin gels......6

Section II. Determination of mechanical properties of fibrin and agarose gels......6

Section III. Confinement-mediated change in fibrin microgel volume......7

Section IV. Characterization of fibrin filaments in the undeformed gel (Supplementary Table 1)......7

Section V. Theoretical model (Supplementary Tables 2 to 4)......8

Supplementary Figures 1 to 18......21

Supplementary References......28

Supplementary Methods

1. Materials

All chemicals, unless specified, were purchased from Sigma-Aldrich Canada. SU-8 photoresist was supplied by MicroChem. Dow Corning Sylgard 184 Silicone elastomer kit (a polydimethylsiloxane, PDMS, prepolymer and crosslinker) was purchased from Ellsworth Adhesives Canada (Burlington, ON). Fluorinated oil HFE-7500 3M Novec (F-oil) was received from 3M Corporation. SU-8 2025 photoresist and SU-8 developer were purchased from MicroChem Corp. (Newton, MA). AZ40XT photoresist and MIF-300 developer were supplied by Integrated Micro Materials (Argyle, TX). Human

fibrinogen and thrombin were supplied by Enzyme Research Laboratories. Ultralow gelling temperature agarose (SeaPrep) was supplied by Lonza (Switzerland). All chemicals were used as received. Silicon wafers were supplied by Wafer World, Inc. (West Palm Beach, FL).

The triblock copolymer of perfluoropolyether and a poly(ethylene oxide) polypropylene was synthesized as described elsewhere[1] and used as a surfactant for MF droplet generation. Fluorescein isothiocyanate (FITC)-labeled fibrinogen was prepared by modifying fibrinogen with 5-(and-6)-carboxyfluorescein succinimidyl ester (Invitrogen). 143 µL 10 mg/mL 5-(and-6)-carboxyfluorescein succinimidyl ester in dry DMSO was added to 5 mL of mg/mL fibrinogen solution (0.1 M $NaHCO_3$ buffer, pH = 8.0) under stirring at room temperature. After the mixture was stirred for 1 hour in the dark, 100 µL of 1.0 M ethanolamine in 0.1 M $NaHCO_3$ buffer was added to the mixture to stop the reaction. The resultant FITC-fibrinogen solution was dialyzed against Tris buffer saline (TBS, 50 mM Tris, 150 mM NaCl, pH 7.6) at 4 °C in the dark, using 10 kDa molecular weight cutoff membranes, with TBS replaced every 24 hours. Thrombin with blocked active sites was prepared by incubating thrombin with a 50-fold molar excess of Phe-Pho-Arg-chloromethyl ketone (PPACK; Molecular Innovations), followed by dialysis against TBS at 4 °C, with four dialysis exchanges every 24 hours. Tissue plasminogen activator (tPA, 1 mg; Genentech) was buffer-exchanged with 50 mM sodium phosphate, 0.15 M NaCl, 0.5 M ammonium sulfate, pH 7.2 using a PD-10 column. Alexa Fluor 633 C5 maleimide (Molecular Probes) was dissolved in dimethyl sulfoxide to 10 mg/ml and 100 µLwas slowly added to the tPA with continuous mixing. The reaction was conducted at 23 °C in the dark for 2 hours, and the product was dialyzed overnight using 10 kDa MW cutoff dialysis tubing in the same buffer.

2. Generation of microgels

To generate fibrin microgels, a mixture of aqueous solutions of fibrinogen, thrombin and Tris-buffered saline (TBS) was emulsified in F-oil. Supplementary Figure 1a shows a snapshot of the MF generation of the droplets in the MF flow-focusing device[2]. The droplets collected at the outlet of the microchannel, were subjected to gelation for 1 hour at 37 °C and subsequently, transferred to TBS. Fibrin microgels with diameter, D_0, from 150 to 270 µm were obtained by changing the flow rate, Q_c, of F-oil from 0.6 to 2.5 mL/hour, respectively, while maintaining the total flow rate Q_{total} (equal to the sum of the flow rates $Q_{fibrinogen}$, $Q_{thrombin}$ and Q_{buffer}) (Supplementary Fig. 1b).

Uniform mixing of fibrinogen, TBS, and thrombin in the droplets was verified by emulsifying a mixture of fibrinogen solution with 1 mM Methylene Blue dye PPACK-blocked thrombin and TBS, and subsequently, analyzing the distribution of the intensity of the color of the dye in the droplets with a Casio EX-F1 high-speed camera (Movies *S*1 and *S*2). In addition, a uniform distribution of fibrin throughout the microgel was verified by covalently labeling fibrinogen molecules with FITC and examining the variation in fluorescence intensity in the microgel using confocal fluorescence microscopy. Image analysis was conducted for the undeformed microgels[3]. The relative standard deviation in fluorescence intensity throughout the microgel interior was 12.0% ± 1.4% (based on the analysis of > 40 microgels), indicating that a swollen microgel had a uniform distribution of fibrin.

Agarose microgels were produced by the MF emulsification of an aqueous agarose solution with polymer concentration of 2.0, 3.0, 4.0, or 5.0 wt% and subsequent gelation of the precursor droplets at 4 °C for 20 min, following the procedure reported elsewhere[4].

3. Microgel characterization

3.1 Charaterization of fibrinogen/thrombin composition within precursor droplets and microgels

The concentration of fibrinogen and thrombin in the precursor droplets (and corresponding microgels) was determined by supplying a known amount of Methylene Blue dye mixed with each stream and analyzing pixel intensity of the resultant droplets. A calibration curve was generated by relating the pixel intensity for the droplets to the concentration of Methylene Blue in the solution (Supplementary Fig. 2). To determine fibrinogen concentration, the droplets containing a mixture of the fibrinogen solution with 1.0 mM Methylene Blue, PPACK-blocked thrombin, and TBS were generated at the varying ratios of flow rates of the individual solutions of fibrinogen and thrombin, at the constant flow rate of TBS. Droplet images were captured using a QIMAGING-QIClick camera. The average pixel intensity in the droplets was determined using a MATLAB program and corrected by subtracting the pixel intensity value of the background. The same method was used to find the concentration of thrombin in the droplets, with 1.0 mM Methylene Blue introduced in the thrombin solution. Supplementary Figure 3 shows the composition of fibrin gels studied in the present work.

3.2 Measurement of gel permeability and pore sizes

Darcy's law[5] was used to characterize the permeability of fibrin microgels with different compositions, as described elsewhere[6]. Experiments were conducted for macroscopic gels with the same composition as the microgels prepared by the MF method. A mixed solution of fibrinogen, TBS, and thrombin was transferred into a 13.7 mm × 3 mm × 3 mm (length × width × height) sealed chamber fabricated in PDMS and equilibrated for 2 hours at room temperature. The ends of the chamber were connected to inlet and outlet reservoirs containing TBS (50 mM Tris, 150 mM NaCl, pH 7.6) *via* perfluoroalkoxyalkane (PFA) tubing (IDEX Health & Science). The variation of the height of the inlet reservoir with respect to the outlet reservoir controlled the pressure difference across the gel. A constant pressure difference, ΔP = 3175 Pa, was applied across the entire MF setup. The volumetric flow rate, Q_P, of TBS through the gel with varying compositions was determined by measuring the change in the weight of the outlet reservoir over a defined time interval.

3.3 Measurement of mechanical properties of fibrin and agarose microgels

The stiffness of the fibrin microgels was determined using a micropipette aspiration technique. A fused silica capillary (Molex Inc., IL, U.S.A.) with an inner diameter of 76 μm was connected to a manometer using PFA tubing. The capillary and PFA tubing were filled with a 0.01 wt% solution of Tween 20 in TBS. The pressure, $\Delta P'$, at the capillary tip was controlled with the manometer. A microgel was trapped without excess pressure at the opening of the microcapillary mounted on the stage of an inverted microscope (Olympus CKX 41). A particular $\Delta P'$ was applied to the microgel and subsequently, increased in a stepwise manner at a rate of 25 Pa/min. The change in the microgel shape was monitored using a microscope camera (QImaging QIClick). Representative bright field images of the microgel under respective aspiration pressures are shown in Supplementary Figs. 4a-c.

The elastic moduli of agarose microgels with concentrations from 2.0 to 5.0 wt% were in the range from 2.6 to 20.2 kPa, measured using atomic force microscopy (AFM)[7]. Supplementary Discussion II describes the conversion of the elastic modulus to gel stiffness.

3.4 Characterization of fibrin microgel structure

Microgel structure was examined using scanning electron microscopy (SEM). The microgels were fixed for 2 hours using a 5 wt% glutaraldehyde solution in TBS. The wafer supporting fibrin microgels was placed in a microporous specimen capsule (30 μm pore size, Canemco-Marivac), and

water in the microgels was gradually replaced with ethanol by consecutively submerging the capsules in 20, 40, 60, 80, and 100% (v/v) ethanol/water mixtures. The capsule was placed in an Autosamdri-810 Tousimis critical point drier, in which the ethanol was exchanged with liquid CO_2. The liquid CO_2 was brought to a supercritical state. Slow venting of the chamber produced the dried samples, which were then gold-sputtered using a SC7640 High Resolution Sputter Coater (Quorum Technologies) for 15 s at 2.0 kV and 20 mA. Subsequently, the samples were imaged by SEM using a Quanta FEI Scanning Electron Microscope. Supplementary Figure 5 shows SEM images of the microgels obtained from the droplets generated at $Q_{fibrinogen}/Q_{total}$ ratios of 0.13 (A), 0.33 (B) and 0.53 (C), referred to as SM, MM and RM in the main text, respectively. Fiber diameters varied from 10 to 180 nm, as shown in the inset. Supplementary Figure 6 shows the SEM images of the microgels one day after their preparation and after 1 month-long storage at 4 °C , with no noticeable change in microgel structure.

The structure of fibrin and plasma microgels after confinement in the constriction of the MF channel was examined using Transmission Electron Microscopy (TEM). After a 1 hour confinement (*see* section Supplementary Methods 4 and Supplementary Fig. 7f), the microgels were rapidly (in less than 1 min) ejected into a 2 mL centrifuge tube containing 1 mL of 5 wt% solution of glutaraldehyde in TBS for 2 hours. Subsequently, the microgels were washed by TBS using three cycles of centrifugation at 185 *g* for 2 min. After the supernatant was removed, 500 μL of 1 wt% solution of OsO_4 in TBS was added in the centrifuge tube to stain the fibrin microgel for 1 hour. The stained microgels were washed by TBS using three cycles of centrifugation at 185 *g* for 2 min. Then, 100 μL of 1 wt% agarose solution (Lonza Rockland Inc., USA) in TBS at 37 °C was introduced into the centrifuge tube containing the stained fibrin microgels. The microgel dispersion in the 37 °C agarose solution was cast on a glass slide. Following agarose gelation at 4 °C for 1 hour, the fibrin microgel-laden agarose gel was cut into 3 × 3 mm cubes, which were subsequently loaded in a 2 mL glass vial. The water in the microgels was gradually replaced with ethanol by consecutively submerging the microgel-laden agarose gel into 20, 40, 60, 80 and 100 % (v/v) ethanol /water mixtures. Agarose gels laden with fibrin microgels were submerged into propylene oxide (PO) for 15 min, to replace ethanol in the microgels with PO, and were then infiltrated with Epon Araldite (E/A) resin by the consecutive immersion of microgel-laden agarose cubes in a 50 vol% solution of Epon Araldite resin (E/A) in PO for 30 min, a 75 vol% E/A in PO for 1 hour and 100 % E/A for 12 hours. All the infiltration steps were performed at room temperature with gentle agitation. The samples were placed into polyethylene capsules (BEEM, Electron Microscopy Sciences), and additional E/A resin was added to fill the capsules. The capsules with the samples were placed in an oven for 48 hours at 60 °C to polymerize E/A resin. The solid resin with embedded microgels was sectioned on an ultramicrotome to 80 nm-thick slices and the slices were collected on 300 mesh copper grid. The samples were stained for 15 min using saturated uranyl acetate, rinsed in distilled water, stained by Reynold's lead citrate for 15 min and subsequently rinsed with distilled water. The slices were imaged in a Hitachi H7000 transmission electron microscope at an accelerating voltage of 75 kV. In a control experiment, the structure of the nondeformed fibrin microgels was examined using an identical protocol.

To image fibrin gels using confocal fluorescence microscopy, a solution of fibrinogen (a mixture of bovine fibrinogen and FITC-labelled human fibrinogen in a 33:1 weight ratio), thrombin and TBS were mixed to prepare gels with compositions corresponding to SM, MM, and RM. A droplet of the mixed solution was placed on a glass slide with a silicone spacer and covered with a glass coverslip. The samples were then sealed and the mixed solution was incubated at 37 °C overnight to allow for gel formation. The structure of the hydrogel was imaged using a Nikon A1R Confocal Microscope with a 100X oil immersion lens and 488 nm laser.

4. Fabrication of microfluidic devices

Microfluidic devices were fabricated by combining a photoresist reflow method[8] and soft lithography[9]. The details of the fabrication procedure are described elsewhere[10]. The AZ40XT photoresist was used to fabricate the master on a silicon wafer. The photomask used to fabricate the MF device had features of the channel-at-large, transitional channel, and constriction, with the widths of 300, 200 and 50 μm, respectively (Supplementary Fig. 7a). The rectangular cross-sections of the photoresist features were subsequently reshaped into semi-circular *via* a reflow procedure. After replicating the reshaped features in PDMS, two identical topographically patterned PDMS molds were aligned and bonded under the microscope. The cross-section of the transitional channel and constriction are shown in Supplementary Figs. 7b and c. Supplementary Figure 7d shows a smooth transition between the transitional channel and constriction, ensuring a conformal contact between the microgel and the inner wall of the MF channel. After fabrication, the MF devices were maintained in an oven at 140 °C for 12 hours to enhance the hydrophobicity of the surface of the microchannels.

To study lysis of fibrin hydrogels, the MF device shown in Supplementary Fig. 7a was modified. Two symmetric side channels were placed orthogonally to the main microchannel upstream of the constriction (Supplementary Fig. 7e). To scale up the generation of occlusive fibrin microgels for TEM imaging, the transitional channel in the photomask diverged into four parallel tapered regions and four subsequent parallel constrictions (Supplementary Fig. 7f).

5. Microfluidic experiments with biaxially confined microgels

To introduce a microgel into the microchannel, the pressure difference, ΔP, was applied, as shown in Supplementary Fig. 8. Once a microgel (Supplementary Fig. 8a) was trapped in the tapered microchannel section (Supplementary Fig. 8c), ΔP was changed incrementally at a step of 498 Pa by raising the upstream reservoir and maintaining the resultant pressure difference for 10 min (Supplementary Fig. 8b). This time interval allowed the microgel to achieve its equilibrium position: the position of the microgel center (recorded using by a MATLAB program) did not change more than 1 μm within 5 hours (Supplementary Fig. 8b, insert).

To characterize the flow rate of liquid through a microgel, we used fluorescence recovery after photobleaching (FRAP) in combination with confocal laser scanning microscopy (CLSM)[11]. After introducing a microgel into a microchannel, an intense laser pulse was used to photobleach a rectangular 250 μm × 150 μm region upstream of the occlusive microgel. To monitor fluorescence recovery, a series of 45 confocal laser scanning microscopy images was recorded at the attenuated beam intensity with 5-second intervals between image capturing. The flow rate of TBS through the microgel was calculated from the change in the fluorescence intensity distribution between the images. The position of the microgel center, X_c, was approximated as $X_c = (X_b + X_f)/2$, where X_b and X_f are the positions of the back and front microgel edges, respectively, with respect to the beginning of the constriction, X_0.

The procedure of lysis of fibrin microgels was conducted as follows. Before the lysis experiments, the microgels were incubated for 2 hours in a solution containing native glutamic acid-plasminogen (Glu-Plg) at a concentration of 1.07 μM. Then, microgel dispersion was introduced in the main microchannel (Fig. 3C, main text), until a microgel was trapped in the tapered region of the microchanenl. The Glu-Plg solution was supplied to the main microchannel for the entire period of experiment at ΔP of 400-3000 kPa, required to keep the microgel at a fixed position. To induce microgel lysis, a solution of 7.4 nM Alexa Fluor 633-labeled t-PA in TBS was infused at a flow rate of 5.6×10^7 μm^3/s and an additional pressure difference of 0.7 Pa from a channel placed orthogonally to the long axis of the main microchannel (Supplementary Fig. 7e). The entire experiment was imaged

with either a monochrome microscope camera (at an interval of 30 seconds), or a fluorescence multi-channel camera (at an interval of 60 seconds) for multi-color-channel imaging.

Supplementary Discussion

Section I. Determination of permeability and pore size of fibrin gels

Fibrin gel permeability was determined by measuring the volumetric flow rate, Q_p of TBS through the hydrogels with five compositions corresponding to those in Supplementary Fig. 3 in the Supplementary Information. The Darcy's constant[5], K_s, was deduced using the relationship[6]

$$K_s = \frac{\eta L_p Q_p}{A \Delta P}, \quad (1)$$

where A is the cross-sectional area of the gel (9 mm^2), L_p is the gel length (L_p = 13.7 mm), η is the viscosity of water at room temperature (1.002 cP) and ΔP is the applied pressure difference. The value of ΔP was in the range from 635 to 3175 Pa. The relationship between K_s and Q_p satisfied a linear relationship in the selected ΔP range.

The average pore diameter, ξ, of fibrin hydrogel was estimated as[12]

$$\frac{\xi}{2} = \sqrt{\frac{8K_s}{1\text{-}\phi_f}} \quad (2)$$

where ϕ_f is the fiber volume fraction determined from the relationship between $C_{fibrinogen}$ and ϕ_f, as described elsewhere[13]. The pores sizes in gels with different compositions are listed in Supplementary Table 1.

Section II. Determination of mechanical properties of fibrin and agarose gels

The negative pressure differential, $\Delta P'$, applied to the microgel, was related to the aspirated length of the microgel, $(x_i - x_{i,0})$, by the relationship

$$\Delta P' = S(x_i - x_{i,0})/R_c, \quad (3)$$

where R_c is the inner capillary radius, and $x_{i,0}$ and x_i are the intrusion lengths of the microgel into the capillary at $\Delta P'$ = 0 and at $\Delta P'$, respectively. The slope, S, of the plot of $\Delta P'$ vs. $(x_i - x_{i,0})/R_c$, is the microgel stiffness. For incompressible, linear elastic materials the stiffness S is proportional to the elastic modulus, however the microgels were compressible and lost water under stress. Therefore, we adhered to the description of the slope of $\Delta P'$ vs. $(x_i - x_{i,0})/R_c$ as an "apparent stiffness"[14].

Supplementary Figure 4d shows the plot of $\Delta P'$ vs. $(x_i - x_{i,0})/R_c$ for RM. The stiffness S was extracted as the slope of the $\Delta P'$ vs. $(x_i - x_{i,0})/R_c$ graph, shown with the red linear fitting line in the strain range before the onset of non-linearity. The value of S increased from ~850 to 3600 Pa with an increasing fibrinogen-to-thrombin concentration ratio (Supplementary Fig. 4e), in agreement with earlier findings that fibrin gels with short, thin, and highly branched fibers formed at high fibrin content are stiffer than the gels with long, thick and loosely packed fibers, formed at low fibrin concentration[15].

The stiffness of the agarose gels was estimated using the relationship between the stiffness S and the measured elastic modulus G[7] of a gel with uniaxial Poisson ratio, ν_u[16]

$$G = \beta S, \tag{4}$$

where the parameter β depends on ν_u and the ratio $\frac{D_0}{2R_c}$

$$\beta = \beta\left(\nu_u, \frac{D_0}{2R_c}\right) \tag{5}$$

Here, D_0 is the gel diameter, and R_c is the radius of the aspirating capillary. For fibrin gels, measurements of stiffness were preformed for microgels with D_0 = 250 µm and R_c = 38 µm, that is, $\frac{D_0}{2R_c}$ = 3.3. Under similar conditions, for agarose gels, from the interpolating function developed for β following previous publication[16], we obtained β = 1.4, and hence, $S \approx G/1.4$.

Section III. Confinement-mediated change in fibrin microgel volume

Upon biaxial compression of a fibrin microgel in the constriction, its volume, V, greatly reduced, in comparison with its original volume, V_0. For fibrin microgels at D_0/d_c = 4.0 (Supplementary Fig. 9), approximately 90% of water left the microgel interior. The reduction in V agreed with the calculation of the change in shape of the spherical microgel with diameter D_0 to a cylinder with a base diameter of d_c and length of $1.1D_0$. Supplementary Figure 9 shows that the relative reduction in volume of the biaxially compressed fibrin microgels was not dependent on fibrin concentration in the microgels.

Section IV. Characterization of fibrin filaments in the undeformed gel

The dimensions of fibrin fibers within undeformed SMs, MMs and RMs were measured by analyzing the SEM and CFM imaging of the gels. The filaments for analysis were selected with the criterion that both ends of the filament are from a branching point of the fibers. Supplementary Figure 10a and b shows exemplary SEM and CFM images of SM used for analysis, with red dots indicating two ends of a filament and a red arrow pointing perpendicularly to the filament. The end-to-end distance (r), contour length (l), and diameter (d) of the filaments were measured and statistically analyzed with ImageJ software, as indicated in Supplementary Fig. 10c. The shape of the filament projection was approximated by a circular arc with the curvature $\varkappa$ and central angle ω. The central angle of the arc and its curvature were determined as

$$l/r = \omega / \left(2\sin\frac{\omega}{2}\right) \tag{6}$$

$$r = 2\varkappa^{-1}\sin\frac{\omega}{2} \tag{7}$$

Supplementary Table 1. Fibrinogen concentration, $C_{\text{fibrinogen}}$, permeability, K_s, fiber volume fraction, ϕ_f, mesh (pore) size, ξ, and stiffness, S, of undeformed fibrin microgels

$C_{fibrinogen}$ (mg/mL)	K_s (10^{-9} cm^2)	ϕ_f	ξ (µm)	S (kPa)
5.25 (**SM**)*	1.5910	0.024	2.28	0.85
14.4	0.0837	0.061	0.53	1.11
22.8 (**MM**)*	0.0201	0.092	0.27	1.87
30.6	0.0013	0.121	0.07	2.50

37.9 (**RM**)*	0.0009	0.147	0.06	3.60

* Notations SM, MM and RM correspond soft microgel, medium rigidity microgel and rigid microgel, respectively, all described in main text

Section V. Theoretical model

5.1. Structural characteristics of filaments in the undeformed rigid gel

We define "filaments" as fibers connecting the neighboring crosslinking points in the hydrogel. In the undeformed network, a cylinder-shape filament with diameter d, contour length L_0, and elastic modulus E can adopt a straight or a curved shape (Supplementary Fig. 11a and b, respectively). Note that the filament diameter is the same in a 3D gel and when viewed in its 2D projection.

Supplementary Fig. 11b shows a curved filament with an end-to-end distance R_0 and curvature κ_0. We assume that a filament forms an arc with the central angle ψ_0. The radius of curvature of this filament is $\kappa_0^{-1} = L_0/\psi_0$ and

$$L_0 = \psi_0/\kappa_0,$$

$$R_0 = \frac{2}{\kappa_0}\sin\frac{\psi_0}{2} \simeq L_0\left(1 - \frac{1}{24}\psi_0^2\right), \tag{8}$$

The used approximation has 1% accuracy at $\psi_0 = 2$.

Based on experimental observations, an undeformed fibrin gel (Fig. 1B, main text and Supplementary Fig. 10), the filaments were slightly curved, due to their growth mechanism or during the network formation. If the filament is stretched or compressed, the values of L_0, R_0, ψ_0 and κ_0 change to L, R, ψ, and κ, respectively, however the relations in Supplementary Eq. (8) remain valid, that is,

$$L = \psi/\kappa, \quad \text{and}$$

$$R = \frac{2}{\kappa}\sin\frac{\psi}{2} \simeq L\left(1 - \frac{1}{24}\psi^2\right), \tag{9}$$

The structural characteristics of the filaments can be determined from their images, that is, from the 2D filament projections on the observation plane (Supplementary Fig. 12). A curved filament is characterized by L_0, R_0 and deflection P_0 (the distance between the filament center and the end-to-end line) as

$$P_0 = \kappa_0^{-1}[1 - \cos(\psi_0/2)] \tag{10}$$

By dividing Supplementary Eq. (10) by Supplementary Eq. (8), we find the expression for the central angle of the filament

$$\psi_0 = 4\arctan(2P_0/R_0) \tag{11}$$

In the spherical coordinate system, filament orientation is characterized by angles θ_0 and φ_0 (Supplementary Fig. 12). The angle θ_0 determines the orientation of the end-to-end vector with respect to the z-axis (the unconstrained direction), and the angle φ_0 describes the orientation of the filament with respect to its end-to-end vector ($\varphi_0 = 0$ corresponds to the vector P_0 lying in the plane

formed by the vector R_0 and the coordinate axis z in Supplementary Fig. 12). For each filament, these angles are different and none of the filament characteristics can be directly measured from the images. Instead, we directly measure the projections p, r and l of the corresponding characteristics P_0, R_0 and L_0 on the observation x-y plane in Supplementary Fig. 12. These projections depend on the angles θ_0 and φ_0 as

$$p^2 = P_0^2(1 - \sin^2\theta_0 \cos^2\varphi_0) \tag{12}$$

$$r^2 = R_0^2 \sin^2\theta_0 \tag{13}$$

Below we estimate the end-to-end distance, R_0 and deflection, P_0, of the filaments in the undeformed network from the value of r and p on the 2D projection images. We first, assume that all filaments have the same R_0 and P_0, and then estimate the spread of these values. By averaging the expression of Supplementary Eq. (13) over the orientation angles θ_0 using the relation

$$\langle \sin^2\theta_0 \rangle = \frac{1}{2}\int_0^{\pi} \sin^2\theta_0 \sin\theta_0 d\theta_0 = \frac{2}{3}, \tag{14}$$

we find the square of the end-to-end distances of a filament

$$R_0^2 = \frac{\langle r^2 \rangle}{\langle \sin^2\theta_0 \rangle} \tag{15}$$

Here and below the sign $\langle \cdots \rangle$ means the average over all filament projections on the 2D image plane for isotropic undeformed networks.

By dividing Supplementary Eq. (12) by Supplementary Eq. (13) , we find

$$\frac{p^2}{r^2} = P_0^2\left(\frac{1}{r^2} - \frac{\cos^2\varphi_0}{R_0^2}\right) \tag{16}$$

The angular average of this equation is

$$\langle \cos^2\varphi_0 \rangle = \frac{1}{2\pi}\int_0^{2\pi} \cos^2\varphi_0 d\varphi_0 = \frac{1}{2} \tag{17}$$

By solving the average of Supplementary Eq. (16) for P_0^2, we find the square of the deflection

$$P_0^2 \simeq \frac{\langle p^2/r^2 \rangle}{\langle 1/r^2 \rangle - 1/(2R_0^2)}, \tag{18}$$

Instead of the maximum deflection p one can measure the lengths l of the filament projections. To find p for a given l, we approximate the shape of the filament projection by a circular arc with the curvature $\varkappa$ and the central angle ω (Supplementary Fig. 12). With 1% accuracy at $\omega \lesssim 2$, $l = \omega/\varkappa$, $r \simeq \varkappa^{-1}(\omega - \omega^3/24)$ [*see* Supplementary Eq. (9)] and $p \simeq \varkappa^{-1}[1 - \cos(\omega/2)] \simeq \varkappa^{-1}\omega^2/8$ [*see* Supplementary Eq. (10)]. By combining these equations, we find

$$p \simeq \frac{1}{4}\sqrt{6l(l-r)} \tag{19}$$

The filament diameter d is equal to its projection value on the 2D image. Supplementary Table 2 shows the average values of $\langle r^2 \rangle$, $\langle 1/r^2 \rangle$, $\langle p^2/r^2 \rangle$ obtained from the SEM images of fibrin hydrogels,

as well as the mean d and standard deviations. Here and below, we use the notation R_0 and d for the corresponding average values $\widetilde{R}_0$ and $\tilde{d}$ of specific filaments.

Supplementary Table 2. Measured characteristics of filaments in fibrin gel

Gel*	N**	$\langle r^2\rangle$ (μm^2)	$\langle 1/r^2\rangle$ (μm^{-2})	$\langle p^2/r^2\rangle$	$d\pm\langle \Delta d^2\rangle^{1/2}$ (μm)	$\langle\omega\rangle$
SM	37	4.85	0.34	0.022	0.18 ± 0.04	1.06
MM	46	1.15	1.25	0.043	0.08 ± 0.02	1.47
RM	46	0.0038	397	0.036	0.01	1.36

*Notations SM, MM and RM correspond soft microgel, medium rigidity microgel and rigid microgel, respectively, all described in main text

** Number of filaments analyzed in scanning electron microscopy images

Supplementary Table 3 shows the root-mean-square end-to-end distance of the filaments (calculated using the data in Supplementary Table 2) from $\langle r^2\rangle$ as $R_0 = \sqrt{3\langle r^2\rangle/2}$ [Supplementary Eq. (15)] and deflection P_0 from Supplementary Eq. (18). The central angle of the filament, ψ_0, was found from the values of R_0 and P_0 calculated from Supplementary Eq. (11). For all filaments with length L_0 comparable to the average distance ξ between the filaments, the central angle $\psi_0 \simeq 1$ (*see* Supplementary Table 4 for the value of ξ). Supplementary Table 3 also shows the ratio $R_0\psi_0/d \simeq 8P_0/d$, that characterizes the deviation of the filament shape from a straight rod, which can be neglected only if this ratio is on the order of unity.

Supplementary Table 3. Calculated characteristics of filaments

Gel*	$R_0\pm\langle \Delta R_0^2\rangle^{1/2}$ (μm)	ψ_0	$R_0\psi_0/d$
SM	2.69 ± 0.91	0.85	14.4 ± 6.1
MM	1.31 ± 0.23	1.25	19.5 ± 6.5
RM	0.07 ± 0.02	1.11	8.36

*Notations SM, MM and RM correspond to soft microgels, medium rigidity microgels and rigid microgels, respectively.

Along with the averages R_0 and d, Supplementary Tables 2 and 3 show their standard deviations $\pm\langle \Delta R_0^2\rangle^{1/2}$ and $\pm\langle \Delta d^2\rangle^{1/2}$. These deviations enable the evaluation of the applicability of the above-used approximation of the same R_0 and P_0 values for all filaments. Although the mean square deviation of the filament diameter $\tilde{d} = d + \Delta d$ can be estimated from its projection, the mean square deviation of the filament end-to-end distance $\widetilde{R}_0 = R_0 + \Delta R_0$ cannot be obtained from its projection, and we estimate it from the 2-nd and the 4-th moments of the end-to-end distance projection $r = \tilde{R}_0|sin\theta_0|$ as

$$\langle r^2\rangle = \frac{2}{3}\langle \tilde{R}_0^2\rangle, \quad \langle r^4\rangle = \frac{8}{15}\langle \tilde{R}_0^4\rangle \tag{20}$$

since we have $\langle \sin^4\theta_0\rangle = 8/15$.

Supplementary Equations (20) are the generalizations of Supplementary Eq. (15) for the case of the second and fourth moments of the random variables $\tilde{R}_0$, which can be characterized by the dimensionless ratio

$$\alpha_0 = \frac{\langle \tilde{R}_0^4 \rangle}{\langle \tilde{R}_0^2 \rangle^2} \simeq \frac{5}{6} \frac{\langle r^4 \rangle}{\langle r^2 \rangle^2} \tag{21}$$

By evaluating the value of α_0 for SM, MM and RM, we find it to be 1.39, 1.16 and 1.34, respectively. The proximity of α_0 to unity justifies the model described above, which assumes that all filaments have the same end-to-end distance R_0 and deflection P_0, and differ only in their orientation. Below, we will use these values of α to estimate the width of the distribution of the value of $\widetilde{R}_0$.

Assuming the Gaussian shape of this distribution, we find

$$\langle \tilde{R}_0^2 \rangle = R_0^2 + \langle \Delta R_0^2 \rangle, \quad \langle \tilde{R}_0^4 \rangle = R_0^4 + 6R_0^2 \langle \Delta R_0^2 \rangle + 3\langle \Delta R_0^2 \rangle^2, \tag{22}$$

and by solving Supplementary Eqs. (S22) for $\langle \Delta R_0^2 \rangle$ and R_0^2, we find the mean square deviation $\langle \Delta R_0^2 \rangle$ as a function of the dimensionless ratio in Supplementary Eq. (21) as

$$\langle \Delta R_0^2 \rangle = R_0^2 \left[\sqrt{2/(3-\alpha_0)} - 1 \right] \tag{23}$$

This equation was used to estimate the errors in R_0 and $R_0\psi_0/d$ in Supplementary Table 3.

5.2 Is the elasticity of fibrillar hydrogels enthalpic or entropic?

Entropic gels. It is usually assumed that the elasticity of fiber networks has an entropic origin, due to the presence of transverse fluctuations (undulations) of otherwise straight filaments (Supplementary Fig. 11a).[18] The hydrogel network is modelled as a collection of thermally fluctuating straight filament segments of length $L_0 \ll l_p$, where

$$l_p \simeq \frac{Ed^4}{kT} \tag{24}$$

is the persistence length of the filament with diameter d_0, Young's modulus E and kT is the thermal energy. Thermally driven undulations reduce the end-to-end distance of the filaments that can be considered as entropic springs. The force, f, required to extend this spring by a small distance δR is $f \simeq (kTl_p^2/L_0^4)\delta R$. The gel stress $\sigma \simeq f/\xi^2$ is the force per area of a mesh with the size ξ, and the gel strain is $\gamma \simeq \delta R/R_0$. Thereby, the linear elastic modulus of an entropic gel is

$$G \simeq \frac{\sigma}{\gamma} \simeq \frac{kT}{\xi^2} \frac{l_p^2}{L_0^3}, \tag{25}$$

where we assume that $R_0 \approx L_0$. By solving Supplementary Eqs. (S24) and (S25) for E, we find

$$E \simeq (kT\xi^2 L_0^3 G)^{1/2}/d^4 \tag{26}$$

Substituting the the parameters of fibrin gels in our work (*see* Supplementary Table 4 below), we find for them a significant spread in the values of E (430, 34, and 17 kPa, respectively, for RM, SM and MM). Such a spread can be explained by assuming that the filaments in these gels are composed of a different number of thin fibers (protofibrils) that can slide relative to each other[17]. The Young's modulus $E \simeq E_0/N$ of such multi-fiber filaments is significantly smaller than the the Young's modulus E_0 of individual protofibrils. Since the cross-sectional area of a filament is $d^2 \sim N$, the protofibril modulus is proportional to d^2, $E_0 \simeq EN \sim Ed^2$. The large scatter of E_0 values obtained suggests that protofilaments of different gel types have different diameters as well. Below we show that there is no need to invoke a multi-fiber model to describe filaments with enthalpic elasticity.

Enthalpic gels. Next, we assume that the elasticity of fibrin gels is of enthalpic origin, due to the spontaneous bending of rigid filaments (Supplementary Fig. 11b). We validated this hypothesis by developing a theoretical model for this system and comparing the experimental results with the theoretical predictions. The sections of filaments between neighboring crosslinks are considered to be enthalpic springs with end-to-end distance $R_0 \simeq L_0$ and the filament bending modulus k_b. The force required to extend the spring by a small distance δR is $f \simeq k_b \delta R / R_0^2$. By repeating the derivation of Supplementary Eq. (25) with this force *f*, we find the elastic modulus of the enthalpic gel

$$G \simeq \frac{k_b}{\xi^2 L_0} \tag{27}$$

The filament bending modulus k_b is expressed in terms of filament characteristics as [*see* Supplementary Eq. (47)]

$$k_b = \frac{9\pi E d^4}{4\psi_0^2 L_0} \tag{28}$$

We compare the elastic moduli G of the networks with enthalpic and entropic mechanisms of elasticity, Supplementary Eqs. (25) and (27). The values of G become on the same order at $\psi_0 \simeq \psi_c$, where

$$\psi_c^2 \simeq L_0 / l_p \tag{29}$$

The networks with $\psi_0 > \psi_c$ exhibit the enthalpic mechanism of elasticity, while at $\psi_0 < \psi_c$ the entropic elasticity mechanism is valid. As for fibrin networks $\psi_0 \simeq 1$ and $L_0 < l_p$, they exhibit the enthalpic elasticity. Fluctuations contribute only little to the filament elasticity, which is mainly determined by the enthalpic flexibility of curved rods. The enthalpic elastic modulus of the fibrin gel is

$$G \simeq \frac{9\pi E d^4}{4\xi^2 \psi_0^2 L_0^2} \tag{30}$$

Supplementary Table 4 shows the filament Young's modulus E and its persistence length l_p calculated from Supplementary Eqs. (30) and (24) for the measured gel elastic moduli G (*see* Supplementary Discussion Section II). Note that the values of the filament Young's modulus E in different gels are on the same order, thus confirming our assumption about the enthalpic mechanism of elasticity of fibrin hydrogels.

Supplementary Table 4. Elastic properties of SM, MM and RM and characteristics of the consitiuent filaments. The filament stretching modulus k_s is defined in Supplementary Eq. (43) below.

Gel	G (kPa)	ξ (μm)	E (MPa)	l_p (µm)	k_s (nJ)	k_b (nJ)	k_s/k_b
SM	1.19	2.28	4	4×10^4	220	9.5	23
MM	2.62	0.27	0.8	500	5.3	0.11	46
RM	5.04	0.06	1	0.15	5.5×10^{-3}	0.82×10^{-3}	6.7

5.3 Deformation of filamentous networks

5.3.1 Affine model of filamentous networks

This section describes biaxial deformation of a gel formed by crosslinked stiff filaments. We assume that in the undeformed network all the filaments have the same shape and dimensions, and are randomly oriented and weakly bent (Supplementary Fig. 13a). Biaxial compression occurs along radial *x* and *y* directions, while the extension occurs in the unconstrained z-direction (Supplementary Fig. 13a). Under such compression, filament deformation depends on their orientation with respect to the z-direction (Supplementary Fig. 13b). The filaments that are oriented predominantly along the z-axis (shown by the green color), straighten and stretch (thus increasing their end-to-end distance) by elastic forces. The elongation of the network under compression occurs due to the stretching of these filaments. The filaments oriented predominantly parallel to the biaxial compression directions (shown by the brown and magenta colors) bend stronger than in the undeformed state, thereby decreasing the distance between the crosslinking points.

Below we estimate the distribution of angles of the filaments with respect to *z*-axis. The stretching of the gel in the unconstrained *z*-direction is determined from the condition of the minimum of the total stretching/compression energy of all the filaments, including those with orientations that do not coincide with the principal directions of gel deformation. The bending filament rigidity is significantly lower than the extensional rigidity of the filaments. We will demonstrate that a rigid network extends weakly in the unconstrained direction, even under strong compression, due to the strong asymmetry in filament bending and stretching moduli.

Due to the elastic stress, the polymer network deforms affinely on the length scales greater than the size of the network loop (a sequence of crosslinked filaments forming a minimum-size mesh). Since in a rigid network the loop size is on the order of the distance between the filament ends (neighboring crosslinks), below we describe the deformation of the rigid network within the framework of the “affine model” assuming that the distances between the crosslinks vary proportionally to the macroscopic network deformation[18], as shown in Supplementary Fig. 13. We also assume that the elastic energy of a filament, $U(R)$, depends only on its end-to-end distance, R, and the function $U(R)$ is the same for all filaments of the network. The force acting on the filament is

$$f(R) = \frac{dU(R)}{dR} \tag{31}$$

The stress-free state of the rigid network is determined by the conditions of its preparation, at which the distance R_0 between the filament ends is obtained from the condition of zero force on the filament

$$f(R_0) = 0 \tag{32}$$

Under biaxial compression of the network, the external stress is applied only in the radial *r*-direction (*x*-*y* plane), with no stress in the perpendicular z-direction (Supplementary Fig. 13b, bottom). For a network, deformed at the elongation ratios $\lambda_r = \lambda_y = \lambda_y < 1$ and by the (yet undetermined) extension ratio $\lambda_z > 1$, the two corresponding components of the end-to-end vector of any particular filament in the deformed state are

$$R_\mathrm{r} = \lambda_\mathrm{r} R_{0\mathrm{r}}, \quad R_\mathrm{z} = \lambda_\mathrm{z} R_{0\mathrm{z}}, \tag{33}$$

where the radial and azimuthal components of the end-to-end vector in the undeformed network are

$$R_{0r} = R_0 \sin\theta_0, \quad R_{0z} = R_0 \cos\theta_0 \tag{34}$$

The angle θ_0 characterizes the orientation of each particular filament in the undeformed network with respect to the *z*-axis (Supplementary Fig. 14).

Note that the filament orientation and its end-to-end distance

$$R = \sqrt{R_r^2 + R_z^2} = R_0\sqrt{\lambda_r^2 \sin^2\theta_0 + \lambda_z^2 \cos^2\theta_0} \tag{35}$$

change with network deformation. The deformation ratio for the network in the unconstrained *z*-direction, λ_z, is determined by the balance of elastic forces resulting in the zero stress in this direction.

The elastic energy of the network is equal to the sum of elastic energies of the constituent filaments. The average deformation energy per individual filament $\overline{U}(\lambda_r, \lambda_z)$ depends on the network deformation ratios λ_r and λ_z and can be found by averaging the energy $U(R)$ over the original orientation angle θ_0 of the filaments as

$$\overline{U}(\lambda_r, \lambda_z) = \int_0^{\pi/2} U\,(R) \sin\theta_0 d\theta_0 \tag{36}$$

under the assumption of the uniform distribution of filaments in the undeformed network. The value of λ_z is found from the minimum of the average elastic energy per filament as a function of the degree of network deformation in the unconstrained direction. The gel biaxially compressed in x and y directions adjusts its size in the unconstrained z-direction to minimize its total elastic energy as

$$\frac{\partial \overline{U}(\lambda_r, \lambda_z)}{\partial \lambda_z} = \int_0^{\pi/2} \frac{dU(R)}{dR} \frac{\partial R}{\partial \lambda_z} \sin\theta_0 d\theta_0 = 0 \tag{37}$$

The differentiation of Supplementary Eq. (35) with respect to λ_z yields

$$\frac{\partial R}{\partial \lambda_z} = \frac{R_0^2}{R} \lambda_z \cos^2\theta_0 \tag{38}$$

and by solving Supplementary Eq. (35) for $\cos\theta_0$, we obtain

$$\cos\theta_0 = \sqrt{\frac{R^2/R_0^2 - \lambda_r^2}{\lambda_z^2 - \lambda_r^2}} \tag{39}$$

By substituting Supplementary Eqs. (38) and (39) into the integrand in Supplementary Eq. (37), we obtain the condition for the minimum of the average elastic energy of the filament

$$\int_{R_0\lambda_r}^{R_0\lambda_z} f\,(R) \sqrt{R^2 - R_0^2\lambda_r^2} dR = 0 \tag{40}$$

This equation describes both entropic and enthalpic affine models. It determines the deformation ratio λ_z in the unconstrained direction of the gel for a given ratio $\lambda_r = d_c/D_0$ in the radial direction and a particular force-extension dependence $f(R)$ of the individual filaments (*see* next section). Here D_0 and d_c are the x-y diameters of undeformed and biaxially compressed gel, respectively (Fig. 2c and Supplementary Fig. 13)

5.3.2 Change in elastic energy of the filament upon biaxial confinement-induced gel deformation

The change in the elastic energy of the filament upon its deformation is determined by the difference between its contour length L and the filament end-to-end distance R and their equilibrium values (L_0 and R_0), respectively, in the undeformed gel

$$U(R, L) = U_{\mathrm{s}}(L) + U_{\mathrm{b}}(R, L), \tag{41}$$

where $U_S(L)$ is the stretching energy of the filament

$$U_{\mathrm{s}}(L) = \frac{k_s}{2}\left(\frac{L}{L_0} - 1\right)^2 \tag{42}$$

with the filament stretching modulus

$$k_{\mathrm{s}} = E\frac{\pi d^2 R_0}{4} \tag{43}$$

which is found from the linear theory of rod elasticity[19].

The second term in Supplementary Eq. (41) is the bending energy of a filament with a fixed contour length L and circular cross-section with a radius $d/2$

$$U_{\mathrm{b}} = \frac{\pi E L}{8}\left(\frac{d}{2}\right)^4 (\kappa - \kappa_0)^2 \tag{44}$$

Substituting κ_0 and κ obtained from the solution of Supplementary Eqs. (8) and (9) into Supplementary Eq. (44) one obtains the expression for the bending energy as a function of R and L

$$U_{\mathrm{b}}(R, L) = 3\pi E\left(\frac{d}{2}\right)^4 \left(\frac{\sqrt{L - R} - \sqrt{L - R_0}}{L}\right)^2 \tag{45}$$

By expanding $U_{\mathrm{b}}(R, L)$ for small deviations of filament end-to-end distance R from its equilibrium value R_0 we find

$$U_{\mathrm{b}}(R, L) \simeq \frac{k_{\mathrm{b}}}{2}\left(\frac{R}{R_0} - 1\right)^2, \tag{46}$$

where the filament bending modulus k_{b} is

$$k_{\mathrm{b}} = \frac{9\pi E d^4}{4\psi_0^2 L_0} \tag{47}$$

Since $R_0 \approx L_0$, the ratio of the bending-to-stretching moduli is

$$\frac{k_{\mathrm{b}}}{k_{\mathrm{s}}} \simeq \left(\frac{3d}{L_0 \psi_0}\right)^2 \tag{48}$$

and is $k_{\mathrm{b}}/k_{\mathrm{s}} \ll 1$ when the aspect ratio d_0/L_0 is smaller than the central arc angle, that is, $d/L_0 \ll \psi_0$.

The equilibrium contour length $L_{eq} = L_{eq}(R)$ for a given end-to-end distance R of the filament is found from the minimum of the filament elastic energy using Supplementary Eq. (41)

$$\left.\frac{\partial U(R,L)}{\partial L}\right|_{L=L_{\mathrm{eq}}} = 0, \tag{49}$$

which determines the filament elastic energy $U(R) = U[R, L_{\mathrm{eq}}(R)]$ and the force acting on the filament as in Supplementary Eq. (31). The minimum of filament elastic energy $U(R)$ is in the undeformed state of the gel for $R = R_0$, and at the corresponding contour length $L_{eq} = L_0$. To find the network energy in the biaxially compressed state, the elastic energy $U(R)$ is averaged over end-to-end distances R of all the filaments in the network in the interval $\lambda_r R_0 < R < \lambda_z R_0$,

$$\overline{U}(\lambda_{\mathrm{r}}, \lambda_{\mathrm{z}}) = \int_{R_0\lambda_{\mathrm{r}}}^{R_0\lambda_{\mathrm{z}}} q(R)U(R)dR, \tag{50}$$

where we changed the variable of integration in Supplementary Eq. (36) using Supplementary Eq. (39). The distribution function of the filament end-to-end distances is

$$q(R) = \frac{R}{R_0\sqrt{\lambda_{\mathrm{z}}^2 - \lambda_{\mathrm{r}}^2}\sqrt{R^2 - R_0^2\lambda_{\mathrm{r}}^2}} \tag{51}$$

In the undeformed state, $\lambda_r = \lambda_z = 1$, and the distribution function of the filament end-to-end distances is assumed to be a delta-function, $q(R) = \delta(R - R_0)$. The maximum increase of the end-to-end distance λ_z is for the filaments that are oriented in the z-direction. The maximum decrease of the end-to-end distance λ_{r} is for the filaments oriented parallel the direction of compression. The filaments oriented in other directions in the undeformed state have deformation ratios R/R_0 between λ_r and λ_z, with a maximum of the distribution function $q(R)$ at the edge of the interval at $R = R_0\lambda_r$, *see* Supplementary Fig. 15.

Since the filaments have a high resistance to stretching $k_{\mathrm{s}} \gg k_{\mathrm{b}}$, we split the interval of integration in Eqs. (S40) and (S50) into two sub-intervals: before and after the minimum of the function $U(R)$ at $R = R_0$. For the filaments that are compressed with respect to their undeformed tension-free size R_0, with $\lambda_r R_0 < R < R_0$, the bending energy $U_b(R, L_{eq})$ prevails in Supplementary Eq. (41), with an equilibrium contour length $L_{\mathrm{eq}} \simeq L_0$ from the solution of Supplementary Eq. (49). For extended filaments with $R_0 < R < \lambda_z R_0$, the stretching energy $U_S(L_{eq})$ prevails in Supplementary Eq. (41) with the equilibrium contour length $L_{\mathrm{eq}}(R) \simeq R$ (Supplementary Eq. (49)) corresponding to straight filaments with curvature $\kappa \simeq 0$.

We conclude that the elastic energy of stretched filaments can be approximated as

$$U(R) = U_s(R) = \frac{k_s}{2}\left(\frac{R}{R_0} - 1\right)^2 \quad \text{for } R > R_0 \tag{52}$$

and for the compressed filaments can be approximated as

$$U(R) = U_b(R, L_0) = \frac{k_b}{2}\left(\frac{R}{R_0} - 1\right)^2 \text{ for } R < R_0 \tag{53}$$

By substituting expressions of Supplementary Eqs. (52) and (53) into Supplementary Eq. (31), we find the forces acting on the stretched and compressed filaments as

$$f(R) = k_{\mathrm{s}}(R - R_0)/R_0^2 \quad \text{at } R > R_0$$

$$f(R) = k_{\mathrm{b}}(R - R_0)/R_0^2 \quad \text{at } R < R_0 \tag{54}$$

By calculating the integral in Supplementary Eq. (40) with this function $f(R)$, we find the expression for the deformation ratio λ_z of the network in the unconstrained direction

$$2(\lambda_z^2-\lambda_r^2)^{3/2}-3\lambda_z\sqrt{\lambda_z^2-\lambda_r^2}+3\lambda_r^2\ln\left(\lambda_r^{-1}+\sqrt{\lambda_r^{-2}-1}\right)$$

$$+\left(\frac{k_b}{k_s}-1\right)\left[-(1+2\lambda_r^2)\sqrt{1-\lambda_r^2}+3\lambda_r^2\ln\left(\lambda_r^{-1}+\sqrt{\lambda_r^{-2}-1}\right)\right]=0 \tag{55}$$

The solution of Supplementary Eq. (55) for λ_z depends only on the ratio of bending and stretching moduli k_b/k_S [Supplementary Eq. (48)] and lies in the interval $1<\lambda<\lambda_{\max}$. The asymptotic value of the deformation ratio $\lambda_{\max}$ in the limit of strong network compression is obtained from the equation

$$(3-2\lambda_{\max})\lambda_{\max}^2=1-k_b/k_s \tag{56}$$

For $k_b/k_s \ll 1$, the solution of Supplementary Eq. (56) for the maximum network strain in the unconstrained z-direction is $\gamma_{e,\max}=\lambda_{\max}-1\approx(k_b/3k_s)^{1/2}$. With a strong anisotropy of the response of the filament to its bending and stretching, that is at $k_b \ll k_s$, the expression for the network strain γ_e in the unconstrained direction is a function of the deformation ratio λ_r in radial direction:

$$\begin{aligned}\gamma_e(\lambda_r) &= \lambda_z(\lambda_r)-1\\ &\simeq\left(\frac{k_b}{k_s}\right)^{1/2}\left[\frac{2\lambda_r^2+1}{3}-\frac{\lambda_r^2}{\sqrt{1-\lambda_r^2}}\ln\left(\lambda_r^{-1}+\sqrt{\lambda_r^{-2}-1}\right)\right]^{1/2}\end{aligned} \tag{57}$$

The average elastic energy per individual filament in a uniformly deformed biaxially confined gel is determined by Supplementary Eq. (50). By performing calculation similar to the above, we find that for $k_s \gg k_b$

$$\overline{U}(\lambda_r,\lambda_z)\simeq\frac{k_b}{2}\left[\frac{2\lambda_r^2+1}{3}-\frac{\lambda_r^2}{\sqrt{1-\lambda_r^2}}\ln\left(\lambda_r^{-1}+\sqrt{\lambda_r^{-2}-1}\right)\right] \tag{58}$$

The expression in square brackets in Supplementary Eqs (57) and (58) is limited to $1/3$.

Although only bending stiffness k_b and radial compression ratio λ_r are used in Supplementary Eq. (58), it follows from the equilibrium condition as in Supplementary Eq. (37) that the average elastic energy of the filament is on the order of the equilibrium stretching energy, $\overline{U}\simeq k_s\gamma_e^2/2$. Note that Supplementary Eq. (57) for the network strain corresponds to the balance, $k_s\gamma_e^2/2\simeq k_b\gamma_b^2/2$, of the average energies of filament stretching and its bending by the effective strain γ_b.

5.3.3 Deformation of spherical microgel under biaxial confinement in a constriction

Consider a spherical shape gel with a diameter D_0, which is placed in the narrow capillary with a diameter d_c. The fragments and the shape of the gel before and after its biaxial compression in the capillary is shown in the cartoons in Supplementary Fig. 13a and b, respectively.

As described in Supplementary Discussion Section 5.3.1, the deformation of the network is characterized by the elongation ratios, λ_x, λ_y, and λ_z with respect to its undeformed state. The deformation in the compression direction is described by the radial elongation factor $\lambda_r=\lambda_x=\lambda_y=d_c/D_0<1$. The deformation in the direction perpendicular to the compression is described by the longitudinal deformation ratio $\lambda_z=D_z/D_0>1$, where D_z is the length of the network in this

direction. When placed in the constriction, an initially spherical network with the volume $(4\pi/3)(D_0/2)^3$ transforms into a close-to-cylindical shape, and in the limit of strong network compression at $\lambda_r \ll 1$ the volume of the cylinder is $\pi D_z(d_c/2)^2$. The volume V of the network decreases ($V < V_0$), as the solvent is squeezed out of it

$$\frac{V}{V_0} = \frac{3}{4}\frac{D_z(d_c/2)^2}{(D_0/2)^3} = \frac{3}{2}\lambda_z\lambda_r^2 \tag{59}$$

In general, the variation of λ_z *vs.* λ_r in the r-direction can be presented as

$$\lambda_z(\lambda_r) = \frac{S(\lambda_r)\lambda_r^{-2}V(\lambda_r)}{V_0}, \tag{60}$$

where the shape factor $S(\lambda_r)$ depends on the network shape. For the uncompressed spherical network with $\lambda_r = \lambda_z = 1$, $V = V_0$, the shape factor in Supplementary Eq. (60) is $S(1) = 1$. For a strongly compressed network with a cylindrical shape (at $\lambda_r \ll 1$) by comparing Supplementary Eqs. (59) and (60) we find the shape factor $S(\lambda_r = 0) = 2/3$. For the entire range of $\lambda_r \ll 1$, we interpolate the shape factor between the limits of $S(0) = 2/3$ and $S(1) = 1$ as

$$S(\lambda_r) = \frac{2}{3} + \frac{1}{3}e^{c(1-1/\lambda_r)} \tag{61}$$

The constant c in Supplementary Eq. (61) is determined from the conditions of the weak hydrogel compression $1 - \lambda_r \ll 1$, at which the hydrogel deforms only locally near the gel surface, in the central region with the thickness and depth approximately equal to the width of the equatorial ring (depicted by a red ribbon in Supplementary Fig. 16). Since the strain in the gel decreases as the power law of the distance from this surface ring, in the limit $\lambda_r \to 1$ the network size $D_z = D_0\lambda_z$ does not depend on λ_r,

$$\left.\frac{d\lambda_z(\lambda_r)}{d\lambda_r}\right|_{\lambda_r=1} = 0 \tag{62}$$

The above considerations for a network in the constriction are general and can be applied to both entropic gels formed by flexible polymers[20–22] and enthalpic gels. For example, for flexible entropic gels, the volume ratio $V(\lambda_r)/V_0$ induced by biaxial compression was calculated from the balance of elastic stress and osmotic pressure in the network[11]

$$\frac{V(\lambda_r)}{V_0} = \left(\frac{5}{7}\right)^{1/3}\lambda_r^{4/3}\left[\left(\frac{1}{2}+v\right)^{1/3} + \left(\frac{1}{2}-v\right)^{1/3}\right], \tag{63}$$

where $v = \left[1/4 - (8/4725)\lambda_r^{10}\right]^{1/2}$. By substituting Supplementary Eq. (63) into Supplementary Eq. (60) with $S(\lambda_r)$ given by Supplementary Eq. (61), we find that the condition of Supplementary Eq. (62) for flexible gels is satisfied for $c = 0.947$. Supplementary Equation (63) can be used to determine the variation in elongational strain $\gamma_e(\lambda_r)$ under conditions of biaxial compression of flexible entropic gels. For a strongly compressed network, $\lambda_r \ll 1$, the elongation increases as

$$\lambda_z(\lambda_r) = 1 + \gamma_e(\lambda_r) \simeq 0.6\lambda_r^{-2/3}. \tag{64}$$

Figure 2a of the main text exhibits excellent agreement between the experimentally measured and theoretically predicted [Supplementary Eq. (64)] values of elongational strain with progressive confinement of agarose gels in cylindrical constrictions.

For enthalpic gels formed by rigid bent strands, such as fibrin filaments, the dependence of strain $\gamma_e(\lambda_r) = \lambda_z - 1$ in unconstrained direction on biaxial compression is given by Supplementary Eq. (57). Figure 2a in main text shows the agreement between predited and measured vavues of λ_z for SM, MM, and RM confined in the constriction under biaxial compression.

The dependence of volume ratio V/V_0 on $\lambda_r = d_c/D_0$for the filamentous gel for a known function $\lambda_z = 1 + \gamma_e(\lambda_r)$ is found from the solution of Supplementary Eq. (60). To find constant c in Supplementary Eq. (61) for the shape factor $S(\lambda_r)$, we note that under weak compression ($1 - \lambda_r \ll 1$) of a spherical gel in a cylindrical tube, the longitudinal deformation in the direction of the cylinder axis is small, $\lambda_z \simeq 1$. Therefore, the gel volume changes only due to the compression of the sphere in the region of the equatorial ring in Supplementary Fig. 16 to a cylinder with the diameter $d_c = \lambda_r D_0$. By subtracting the volume of the equatorial ring from the undeformed gel volume V_0, we find

$$V(\lambda_r) \simeq V_0\left[1 - (1 - \lambda_r^2)^{3/2}\right] \quad \text{at} \quad 1 - \lambda_r \ll 1 \tag{65}$$

By differentiating this expression with respect to λ_r at $\lambda_z = 1$, we find the condition for determining constant c as

$$\left.\frac{dV(\lambda_r)}{d\lambda_r}\right|_{\lambda_r=1} = 0 \tag{66}$$

Substituting $V(\lambda_r)$ from Supplementary Eq. (60) into Supplementary Eq. (66), we find that for the RM gel this condition is satisfied with $c \simeq 6.5$. The large c value for fibrin gels is associated with the correction of interpolation formulas in Supplementary Eqs. (60) and (65) to take into account the regime of small deformations, $\lambda_r \simeq 1$. Supplementary Figure 9 shows agreement between the calculated and experimental dependence of the volume ratio V/V_0 on the degree of confinement of the RM in the cylindrical constriction.

5.3.4 Effective Poisson's ratio

The biaxial Poisson ratio $\nu_b = \lim_{\lambda_r \to 1} \frac{\lambda_z - 1}{1 - \lambda_r}$ describes the deformation in the z-direction in response to equal deformations in two radial directions. The significant uniform deformations experienced by the gels can be described by the effective Poisson ratio $\nu_b^{eff} = -\ln\lambda_z/\ln\lambda_r$. Deformation in the z-direction is related to the change in the volume of the gel as $\lambda_z(\lambda_r) = V(\lambda_r)/(V_0\lambda_r^2)$, where the dependence $V(\lambda_r)/V_0$ for agarose is given in Supplementary Eq. (63). In the limit of strong radial compression ($\lambda_r \ll 1$), these equations take the form $\lambda_z(\lambda_r) \approx 0.6\lambda_r^{-2/3}$, describing an increase in the value of ν_b^{eff} to its asymptotic value of $2/3$. The dependence $\lambda_z(\lambda_r)$ for fibrin gels is calculated in Supplementary Eq. (57), however, in the limit of strong compression it is simplified as

$$\lambda_z(\lambda_r) \approx 1 + \left(\frac{k_b}{3k_s}\right)^{1/2}\left(1 + \frac{3}{2}\lambda_r^2\ln\lambda_r\right) \quad \text{at} \quad \lambda_r \ll 1 \tag{67}$$

Thus, under strong compression of fibrin gels, their effective Poisson ratio ν_b^{eff} decreases to zero.

5.4 Scaling analysis for the translocation pressure drop for agarose and fibrin gels

5.4.1 Agarose gels

The free energy per unit volume of a fully swollen agarose gel, $\bar{f}$, is given by[11]

$$\bar{f} \sim G\left[\underbrace{\lambda_z^{-5/4}\lambda_r^{-5/2}}_{\text{Osmotic energy}} + \underbrace{2\lambda_z^{-1/4}\lambda_r^{3/2}}_{\text{Elastic energy in } r \text{ direction}} + \underbrace{\lambda_z^{7/4}\lambda_r^{-1/2}}_{\text{Elastic energy in } z \text{ direction}}\right], \quad (68)$$

where G is the elastic modulus of the gel. The axial normal stress is zero, due to the condition $\partial\bar{U}/\partial\lambda_z = 0$, as the microgel is unconstrained in the z-direction [Supplementary Eq. (37)].

$$\sigma_{zz} = 0. \quad (69)$$

For strong compression ratios ($\lambda_r \ll 1$), this equation implies that the osmotic stresses in the gel are balanced by extensional elastic stresses in the z-direction and $\lambda_z^{-5/4}\lambda_r^{-5/2} \sim \lambda_z^{7/4}\lambda_r^{-1/2}$, which yields

$$\lambda_z \sim \lambda_r^{-2/3}. \quad (70)$$

The expression for $\bar{f}$ then simplifies to

$$\bar{f} \sim G\lambda_r^{-5/3}. \quad (71)$$

The radial stress, given by $\sigma_{rr} = \frac{1}{\lambda_r\lambda_z}\frac{d\bar{f}}{d\lambda_r}$[11], becomes

$$\sigma_{rr} \sim G\lambda_r^{-3}. \quad (72)$$

Hence the translocation pressure drop for strong confinement of the gel is[11]

$$\Delta P_{tr} \sim \frac{\lambda_z}{\lambda_r}\tan\alpha\,(2\sigma_{zz} - \sigma_{rr}). \quad (73)$$

Substitution of σ_{rr} and σ_{zz} into the above equation yields

$$\Delta P_{tr} \sim G\tan\alpha\;\lambda_r^{-14/3}. \quad (74)$$

Since gel stiffness $S \sim G$ (*see* Supplementary Discussion Section II),

$$\frac{\Delta P_{tr}}{S} \approx 0.093\tan\alpha\left(\frac{D_0}{d_c}\right)^{14/3}. \quad (75)$$

The prefactor is obtained using the fit of all experimental data for agarose gels in Fig. 2e in main text for $\alpha = 15°$. The normalized translocation pressure drop predicted by the above equation is the green solid curve shown in Fig. 2e in the main text.

5.4.2 Fibrin gels

For fibrin gel experiencing a radial elongation ratio λ_r and an axial extension λ_z, the radial normal stress, σ_{rr}, is[11]

$$\sigma_{rr} = n_{f_0}\frac{\lambda_r}{\lambda_r^2\lambda_z}\frac{\partial\bar{U}}{\partial\lambda_r} = \frac{n_{f_0}}{\lambda_r\lambda_z}\frac{\partial\bar{U}}{\partial\lambda_r}, \quad (76)$$

where $\bar{U}$ is the average energy per filament, and n_{f_0} is the number of fibers per unit volume of the undeformed gel. In the limit $k_b/k_s \ll 1$, the expression for $\bar{U}$ in Supplementary Eq. (58) can be substituted into the above expression for σ_{rr} to yield

$$\sigma_{rr} = \frac{k_b n_{f_0}}{2\lambda_z}\left[\frac{4}{3} + \frac{1}{1-\lambda_r^2} - \frac{(2-\lambda_r^2)}{(1-\lambda_r^2)^{\frac{3}{2}}}\ln\left(\lambda_r^{-1} + \sqrt{\lambda_r^{-2} - 1}\right)\right]. \quad (77)$$

As with agarose gels, the axial normal stress is zero, due to the absence of a geometrical constraint in the z-direction.

$$\sigma_{zz} = 0. \tag{78}$$

The translocation pressure drop is determined as[11]

$$\Delta P_{tr} \sim \frac{\lambda_z}{\lambda_r} \tan\alpha\,(2\sigma_{zz} - \sigma_{rr}) \sim \frac{k_b n_{f_0} \tan\alpha}{2\lambda_r} \left[\frac{(2-\lambda_r^2)}{(1-\lambda_r^2)^{3/2}} \ln\left(\lambda_r^{-1} + \sqrt{\lambda_r^{-2} - 1}\right) - \frac{4}{3} - \frac{1}{1-\lambda_r^2}\right], \tag{79}$$

where λ_r at the constriction entrance is approximated to be $\lambda_r \approx d_c/D_0$. In the strong confinement limit, for fibrin gels $\bar{U} \sim \frac{k_b}{2}\left(\frac{1}{3} - \lambda_r^2 \ln\frac{2}{\lambda_r}\right)$, and $\Delta P_{tr} \propto \lambda_r^{-1} \ln(2e^{-7/6}\lambda_r^{-1})$. Notably, the dimensionless translocation pressure drop, $\Delta P_{tr}/k_b n_{f_0}$ is independent of the bending and stretching moduli in the limit $k_b/k_s \ll 1$. Using $S \approx k_b n_{f_0}$ as the stiffness of the fibrin gel allowed us to fit of the experimental measurements of $\Delta P_{tr}/S$ to the above equation. Hence,

$$\frac{\Delta P_{tr}}{S} \approx \frac{0.65 \tan\alpha}{\lambda_r} \left[\frac{(2-\lambda_r^2)}{(1-\lambda_r^2)^{3/2}} \ln\left(\lambda_r^{-1} + \sqrt{\lambda_r^{-2} - 1}\right) - \frac{4}{3} - \frac{1}{1-\lambda_r^2}\right] \tag{80}$$

The equation

$$\Delta P_{tr}/S \sim \lambda_r^{-1} k_b n_{f_0} \ln(2e^{-7/6}\lambda_r^{-1}) \tag{81}$$

was used to plot the red solid curve in Fig. 2e (main text).

Supplementary Figures

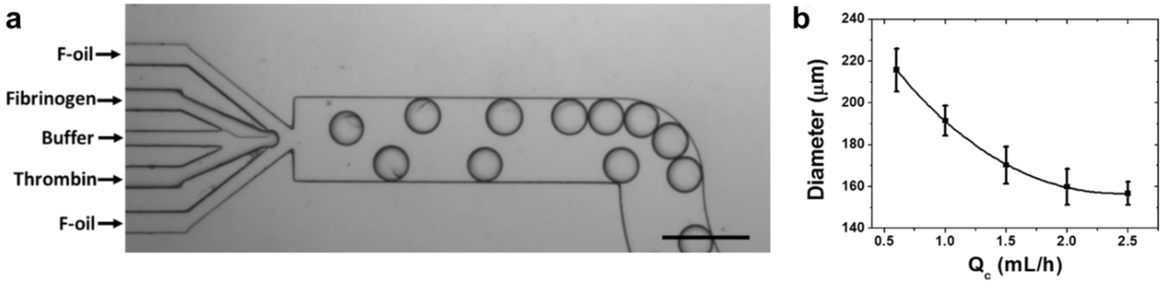

Supplementary Figure 1: Microfludiic generation of microgels. **a**, Generation of precursor droplets in the MF flow-focusing device. The flow rates of the liquids were $Q_{fibrinogen} = Q_{thrombin} = Q_{buffer} = 0.05$ mL/hour. The flow rate, Q_c, of the continuous F-oil phase was 1.5 mL/hour. The scale bar is 500 μm. **b**, Variation in the microgeldiameter, plotted as a function of the flow rate of the F-oil phase, Q_c, at $Q_{total} = 0.15$ mL/hour. For each data point, 100 microgels were analyzed.

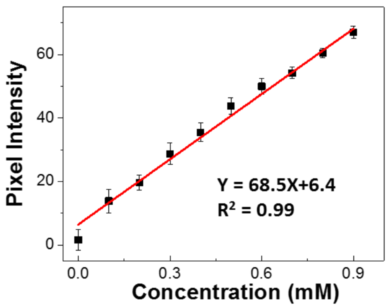

Supplementary Figure 2: Determination of the composition of the precursor droplets and the resulting fibrin microgels. Calibration graph obtained by linear regression fitting the light intensity (black symbols) of the droplets with varying concentration of Methylene Blue. For each data point, a minimum of 30 droplets were analyzed.

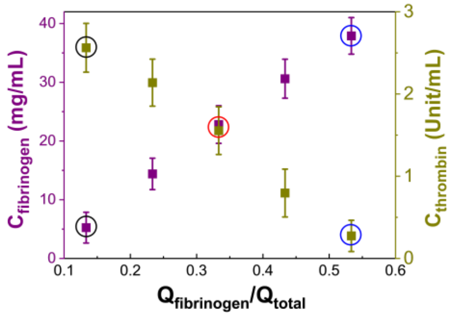

Supplementary Figure 3: Change in composition of fibrin micorgels. Variation in the concentration of fibrinogen and thrombin in the microgels, plotted as a function of the ratio $Q_{\text{fibrinogen}}/Q_{\text{total}}$. Q_c = 0.8 mL/hour, Q_{buffer} = 0.1 mL/hour, and Q_{total} = 0.3 mL/hour. Symbols labeled with black, red, and blue circles correspond to soft microgel (SM), microgel with intermediate rigidity (MM), and rigid microgel (RM), respectively. For each data point, a minimum of 30 microgels were analyzed.

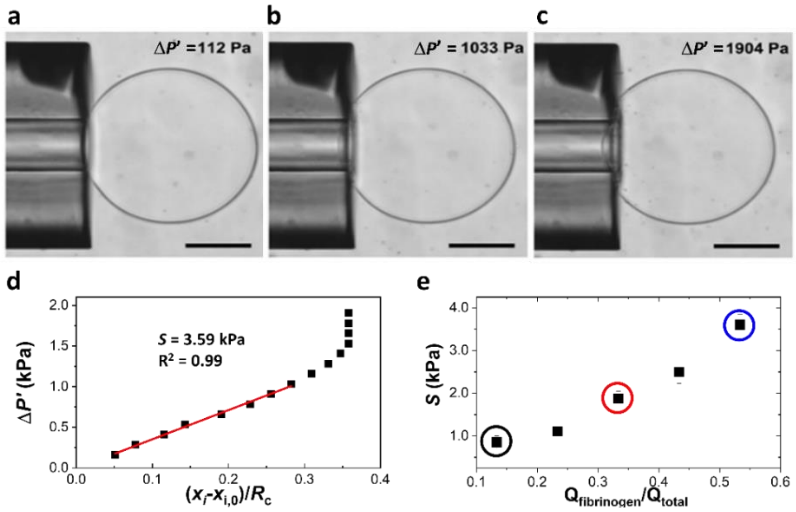

Supplementary Figure 4: Variation in apparent stiffness of fibrin microgels. **a-c**, Optical microscopy images of a fibrin microgel (D_0 = 250 μm) aspirated into a capillary with an inner

diameter of 76 μm. The microgel contained 37.9 mg/mL of fibrinogen and 0.27 U/mL thrombin. The scale bar is 100 μm. (d) Stress-strain relationship for the aspirated microgel shown in (a-c). Red line shows linear fitting of the data points for the linear stress-strain regime, with the slope (apparent stiffness) of 3.59 kPa. (e) Variation in the microgel stiffness, plotted as a function of the ratio of $Q_{\text{fibrinogen}}/Q_{\text{total}}$. Symbols labeled with black, red, and blue circles correspond to SM, MM, and RM, respectively. For each data point, a minimum of 10 microgels were analyzed.

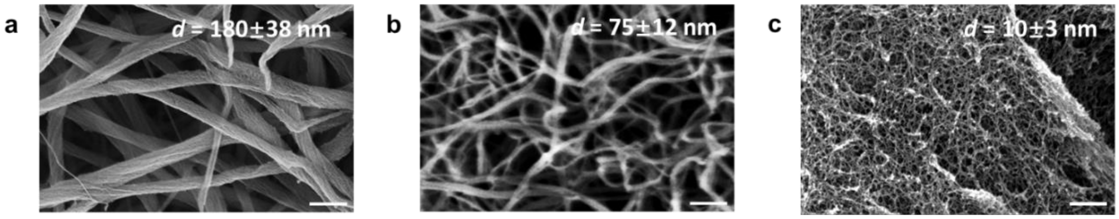

Supplementary Figure 5: Structure of fibrin microgels. SEM images of **a**, SM, **b**, MM and **c**, RM. Scale bars are 500 nm.

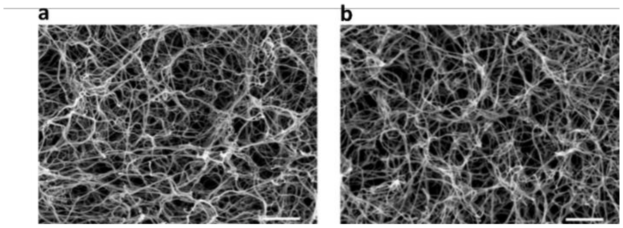

Supplementary Figure 6: Structure of stored fibrin microgels Representative SEM images of fibrin microgels (SMs) **a**, one day and **b**, 1 month after their preparation. The microgels were stored at 4 ºC. Scale bars are 2 μm.

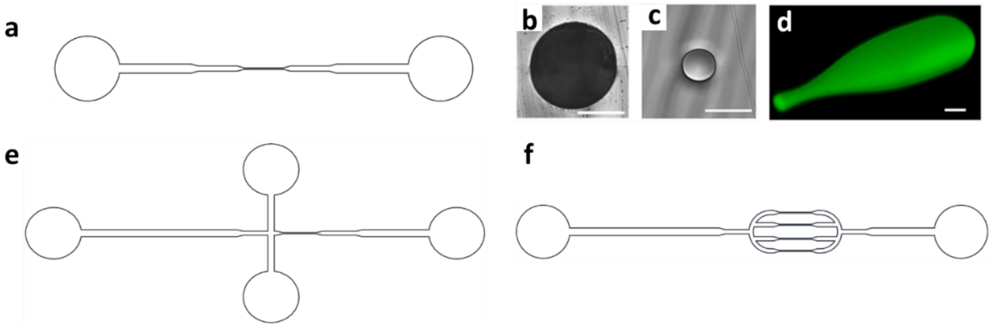

Supplementary Figure 7: Design of the MF devices. **a**, Design of the MF device with a channel-at-large (width of 300 μm, length of 3.3 mm), transitional channel (width of 200 μm, length of 1 mm) and constriction (width of 50 μm, length of 1.5 mm). **b, c**, Cross-sectional view of the transitional channel and the constriction, respectively. **d**, A tilted angle view of the 3D reconstructed microchannel (from b to c). The 3D image was obtained from a *z*-stack of CFM images upon scanning at a step of 5 μm for an entire *z* range of 200 μm. The microchannel was filled with 0.03 mg/mL FITC-Dextran aqueous solution. Scale bars are 100 μm in (b-d). **e**, Design of the MF device for the lysis experiments. Two symmetric side channels (width of 300 μm; length of 1.8 mm) are placed orthogonally to the main channel upstream of the constriction at a distance of 100 μm from the beginning of the tapered region (one of these channels was blocked in the microgel lysing experiment). **f**, Design of the MF device used for the preparation occlusive microgels for TEM imaging.

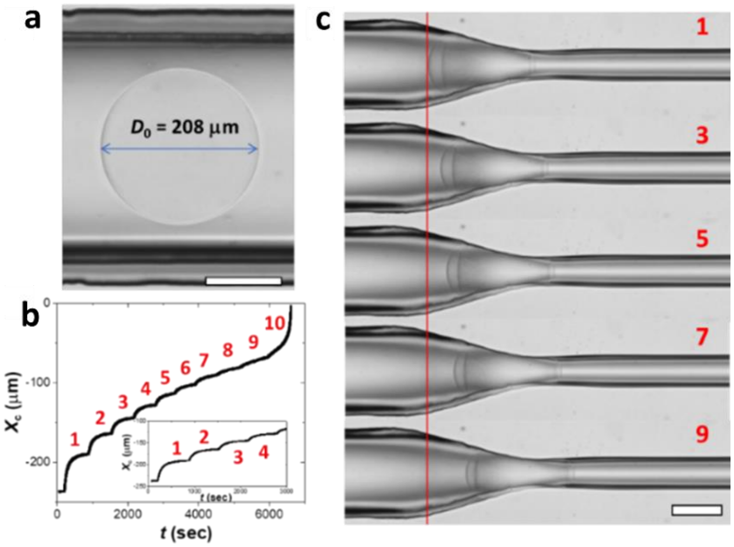

Supplementary Figure 8: Introduction of microgels in the microchannel. **a**, Optical microscopy image of an unperturbed RM (D_0 = 208 μm) in the channel-at-large. **b**, Progression of a RM in the microchannel at increasing applied pressure difference (ΔP). Points 1-10 correspond to applied pressure differences, ΔP, of 1281, 1779, 2277, 2775, 3273, 3771, 4269, 4766, 5264, and 5762 Pa, respectively. Inset shows enlarged microgel progression for ΔP increasing from 1281 to 2775 Pa. **c**, Selected images of RM at positions 1-9, as in (b) over 5 hours. Scale bars are 100 μm.

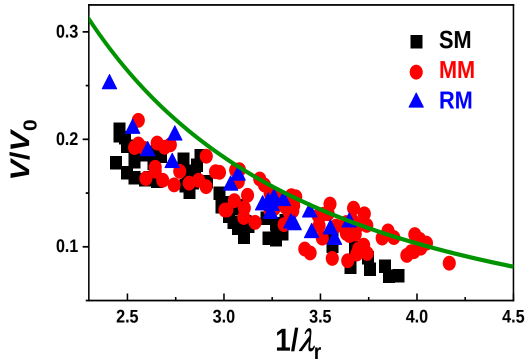

Supplementary Figure 9: Variation in the relative volume of biaxially confined SMs (■), MMs (●), and RMs (▲), plotted as a function of the degree of radial microgel compression. The green solid line show the predicted variation in V/V_0 for $\gamma_e \approx 12\%$. The reduction in V agreed with a prediction of the effect of shape change of the spherical microgel with diameter D_0 to a cylinder with a base diameter of d_c and length of $1.1D_0$.

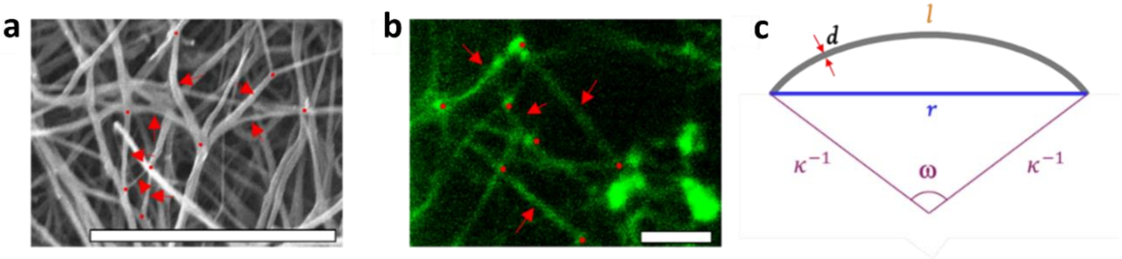

Supplementary Figure 10: Measurement of filament characteristics of fibrin gel from SEM images and CFM images. **a**, Representative SEM and **b**, CFM images of SM structure, with two ends of filaments labeled with red dots, and the center point of the filament marked with a red arrow). Scale bars are 5 µm in (a) and (b). **c**, Measured structural characteristics of a filament: end-to-end distance (*r*), contour length (*l*), and filament diameter (*d*) and determined characterizatics of a filament (curvature $\varkappa$ and central angle ω).

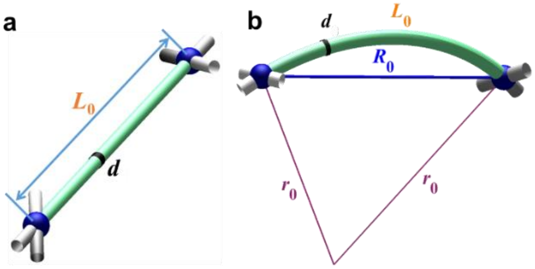

Supplementary Figure 11: Schematic of filaments in an undeformed network. The network is formed by filaments with diameter *d* and contour length L_0. The crosslinks are marked with blue color. **a**, A straight filament with the distance $R_0 = L_0$ betweent two crosslinks. **b**, A curved filament forming a central angle ψ_0, with the radius of curvataure $r_0 = \kappa_0^{-1}$, and the end-to-end distance $R_0 < L_0$.

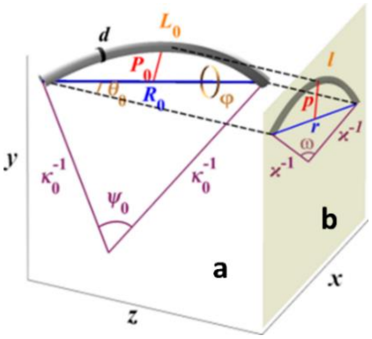

Supplementary Figure 12: A filament in the undeformed network and its 2D projection on the observation plane (highlighted in grey color). a, In the undeformed network, filament orientation is characterized by the angles θ_0 and φ in the spherical coordinate system. **b**, The 2D filament projection is characterized by the characteristics *l*, *p*, *r*, and central angle ω.

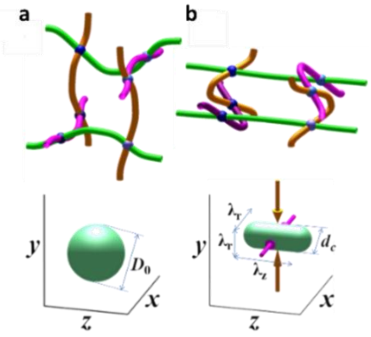

Supplementary Figure 13: Schematic of deformation of fibrin filaments. a, Fibrin filaments before deformation**. b,** Fibrin filaments after biaxial compression**.** Bottom: the shapes of the corresponding networks before and after deformation. The compression directions x and y are shown by the magenta and brown

arrows, respectively. In the top cartoons, the network strands oriented along these x and y directions are shown as the corresponding magenta and brown color lines, while the stands oriented in the unconstrained z-direction are depicted by green lines. The magenta and brown strands oriented in the x and y directions are bent more than in the undeformed state, whereas the green strands oriented in the z-direction are bent and stretched. Stress between compression and stretching directions is transmitted through the filaments with intermediate orientations.

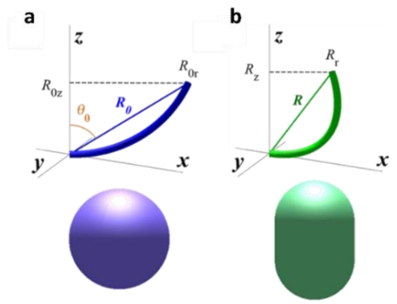

Supplementary Figure 14: Filament coordinates in the (a) undeformed (blue) and (b) deformed (green) states of the gel. The *z*-axis is taken in the unconstrained direction of the network under biaxial compression in the transverse (*x*-*y*) directions. The angle θ_0 characterizes the inclination of the filament (blue) in the undeformed state with respect to the *z*-axis.

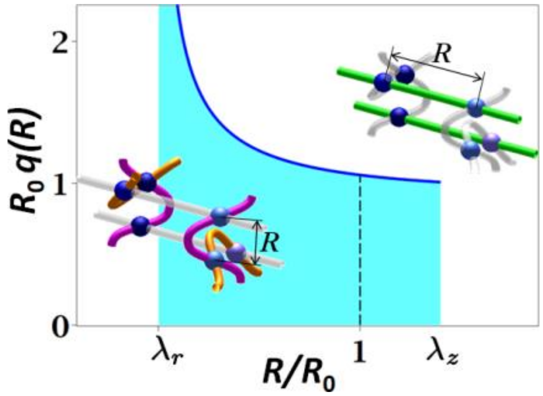

Supplementary Figure 15: Variation in the distribution of the end-to-end distances of filaments. The distribution function $q(R)$ of the end-to-end distances R (Eq. $S51$) for the gel equi-

biaxially compressed for a particular λ_r, and the examples of the configurations of the filaments, contributing to this distribution at different deformation ratios R/R_0.

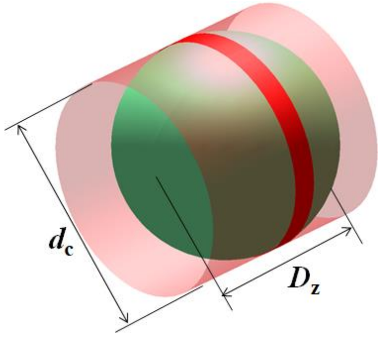

Supplementary Figure 16: Change is shape of the confined hydrogel. Spherical network weakly compressed in the constriction of the diameter d_c and deformed only locally near the equatorial ring (shown in red).

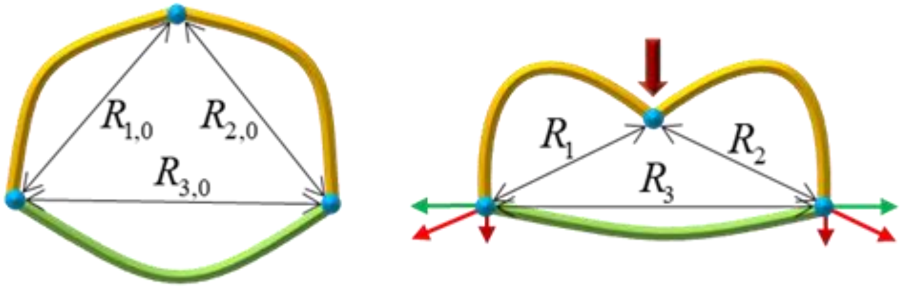

Supplementary Figure 17: Logitudinal stretching of equi-biaxially compressed fibrin gel. Under transverse compression (shown by the upper brown arrow) of the fibrin gel, the filaments oriented at an angle to the compression axis (marked in yellow) are compressed with end-to-end distances $R1 < R_{1,0}$, $R_2 < R_{2,0}$. The forces acting from these filaments (red arrows) have extensional components (green arrows) that stretch the filament (marked in green with end-to-end distance $R_3 > R_{3,0}$) oriented in the direction perpendicular to compression, thus stretching the fibrous network in this direction.

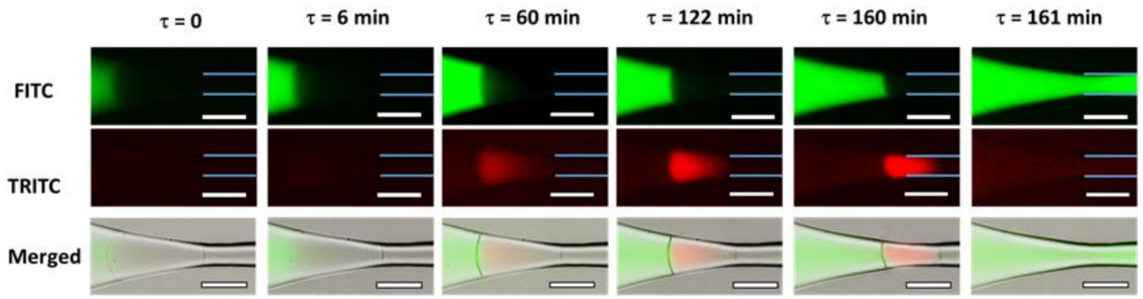

Supplementary Figure 18: Lysis of fibrin microgel (MM). Individual channel and merged-channel images of a microgel (D_0 = 200 μm) during lysis, as in Fig. 3d in the main text. Scale bars are 100 μm. Red color originates from Alexa Fluor 633-labelled t-PA, and green color originates from FITC-Dextran. Horizonal blue lines outline the constriction.

Supplementary References

1. Holtze, C. *et al.* Fluorocarbon emulsion stabilizing surfactants. (2010).
2. Kumacheva, E. & Garstecki, P. *Microfluidic Reactors for Polymer Particles*. (John Wiley and Sons, 2011). doi:10.1002/9780470979228.
3. Bastide, J. & Leibler, L. Large-scale heterogeneities in randomly cross-linked networks. *Macromolecules* **21**, 2647–2649 (1988).
4. Kumachev, A. *et al.* High-throughput generation of hydrogel microbeads with varying elasticity for cell encapsulation. *Biomaterials* **32**, 1477–1483 (2011).
5. Brown, G. O. Henry Darcy and the making of a law. *Water Resour. Res.* **38**, 11-1-11–12 (2002).
6. Prince, E., Chen, Z., Khuu, N. & Kumacheva, E. Nanofibrillar Hydrogel Recapitulates Changes Occurring in the Fibrotic Extracellular Matrix. *Biomacromolecules* **22**, 2352–2362 (2021).
7. Kumachev, A., Tumarkin, E., Walker, G. C. & Kumacheva, E. Characterization of the mechanical properties of microgels acting as cellular microenvironments. *Soft Matter* **9**, 2959–2965 (2013).
8. Wang, G. J., Ho, K. H., Hsu, S. H. & Wang, K. P. Microvessel scaffold with circular microchannels by photoresist melting. *Biomed. Microdevices* **9**, 657–663 (2007).
9. Xia, Y. & Whitesides, G. M. Soft lithography. *Annu. Rev. Mater. Sci.* **28**, 153–184 (1998).
10. Li, Y., Pan, C., Li, Y., Kumacheva, E. & Ramachandran, A. An exploration of the reflow technique for the fabrication of an in vitro microvascular system to study occlusive clots. *Biomed. Microdevices* **19**, 82 (2017).
11. Li, Y. *et al.* Universal behavior of hydrogels confined to narrow capillaries. *Sci. Rep.* **5**, 17017 (2015).
12. Blombäck, B. & Okada, M. Fibrin gel structure and clotting time. *Thromb. Res.* **25**, 51–70 (1982).
13. Wufsus, A. R., MacEra, N. E. & Neeves, K. B. The hydraulic permeability of blood clots as a function of fibrin and platelet density. *Biophys. J.* **104**, 1812–1823 (2013).
14. Kleinberger, R. M., Burke, N. A. D., Dalnoki-Veress, K. & Stöver, H. D. H. Systematic study of alginate-based microcapsules by micropipette aspiration and confocal fluorescence microscopy. *Mater. Sci. Eng. C* **33**, 4295–4304 (2013).
15. Weisel, J. W. Structure of fibrin: impact on clot stability. *J. Thromb. Haemost.* **5**, 116–124 (2007).
16. Boudou, T. *et al.* An extended modeling of the micropipette aspiration experiment for the characterization of the Young's modulus and Poisson's ratio of adherent thin biological samples: numerical and experimental studies. *J. Biomech.* **39**, 1677–1685 (2006).

17. Xia, J., Cai, L. H., Wu, H., MacKintosh, F. C. & Weitz, D. A. Anomalous mechanics of Zn^{2+}-modified fibrin networks. *Proc. Natl. Acad. Sci. U. S. A.* **118**, e2020541118 (2021).

18. Rubinstein, M. & Colby, R. H. *Polymer Physics*. (Oxford University Press, 2003

19. Landau, L.D., Lifshitz, E.M., Theory of Elasticity, Vol. 7 of Course of Theoretical Physics. Second English Edition, Oxford: Pergamon Press, 1975.

20. Flory, P. J. & Rehner Jr., J. Effect of deformation on the swelling capacity of rubber. *J. Chem. Phys.* **12**, 412–414 (1944).

21. Pekarski, P., Tkachenko, A. & Rabin, Y. Deformation-induced anomalous swelling of topologically disordered gels. *Macromolecules* **27**, 7192–7196 (1994).

22. Fujine, M., Takigawa, T. & Urayama, K. Strain-driven swelling and accompanying stress reduction in polymer gels under biaxial stretching. *Macromolecules* **48**, 3622–3628 (2015).
