# Supplementary material for: Fibrous hydrogels under biaxial confinement
Source: Nat Commun. 2022 Jun 7;13:3264. doi: 10.1038/s41467-022-30980-7 (PMC9174476; doi:10.1038/s41467-022-30980-7)
Supplement: Supplementary file 2 — Description of Additional Supplementary Files [file 41467_2022_30980_MOESM2_ESM.pdf]

Description of Additional Supplementary Files

Supplementary Movie 1: Mixing of fibrinogen, thrombin and buffer in the precursor droplets.

Supplementary Movie 2: Precursor droplets moving through the microfluidic device.
