# Supplementary figures and images for: Fibrous hydrogels under biaxial confinement
Source: Nat Commun. 2022 Jun 7;13:3264. doi: 10.1038/s41467-022-30980-7 (PMC9174476; doi:10.1038/s41467-022-30980-7)

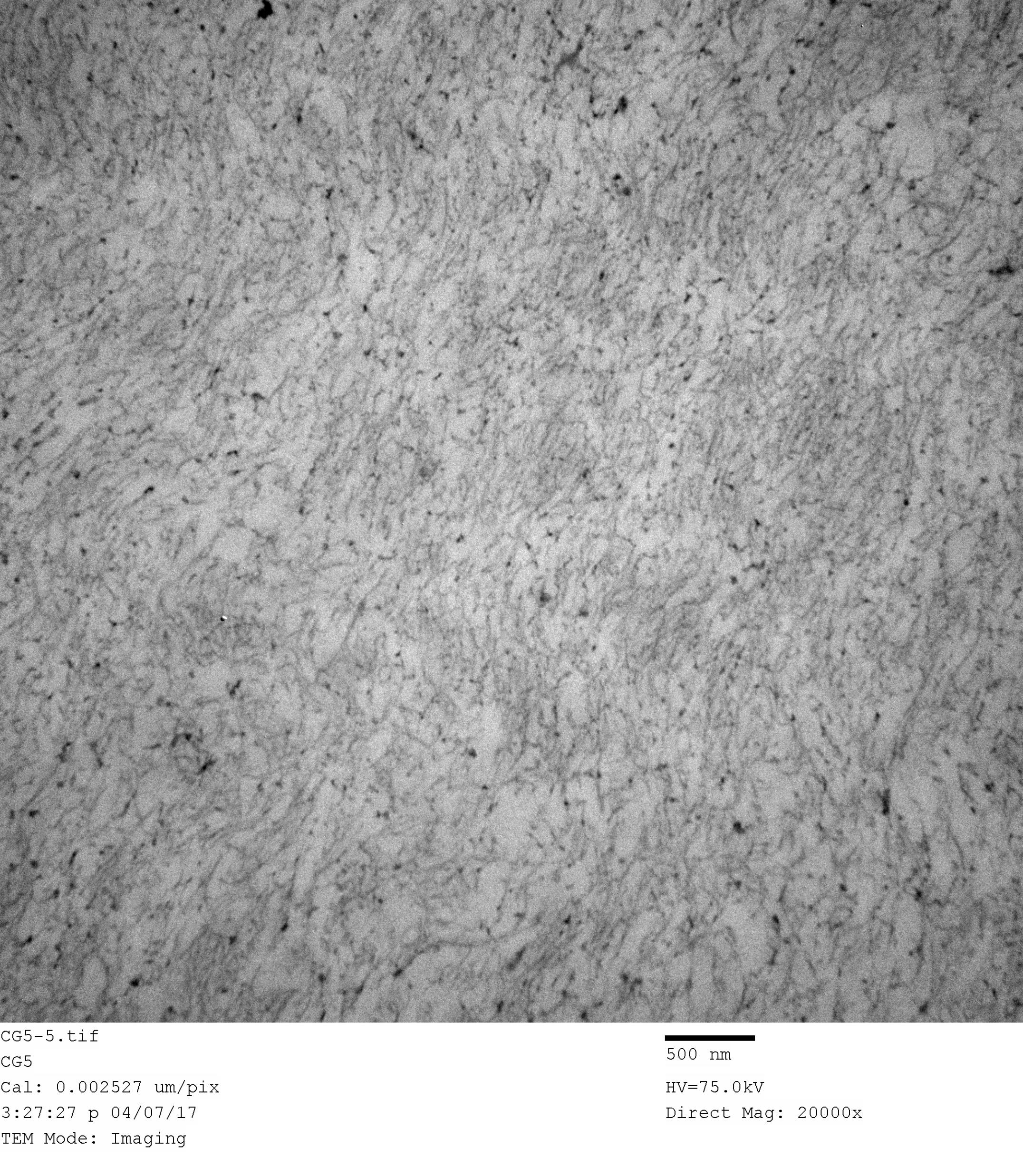

Supplement: Supplementary file 5 — Source Data [file 41467_2022_30980_MOESM5_ESM.zip › TEM images of fibrin gel before and after confinement/After confinement.jpeg]

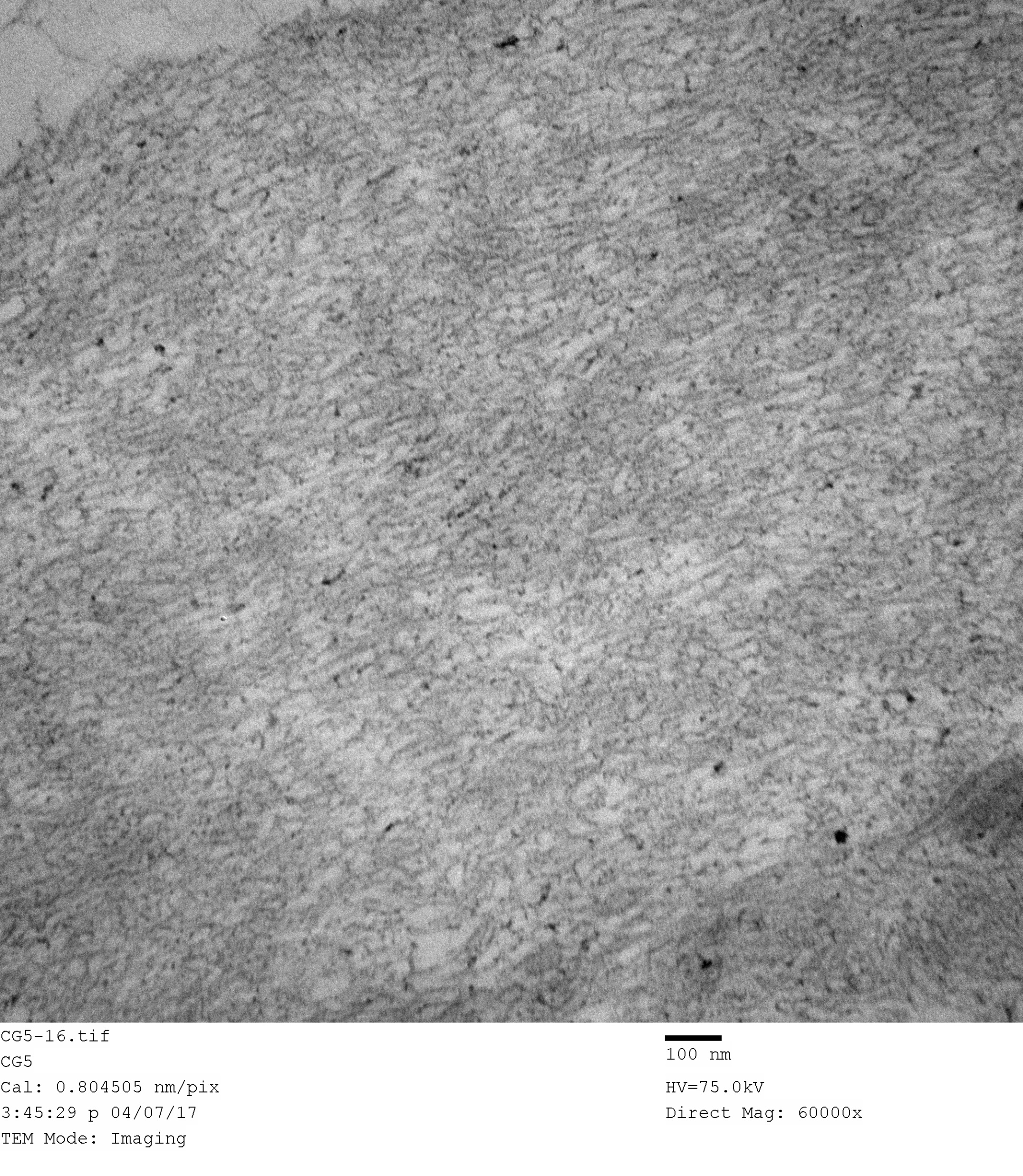

Supplement: Supplementary file 5 — Source Data [file 41467_2022_30980_MOESM5_ESM.zip › TEM images of fibrin gel before and after confinement/After confinement_2.jpeg]

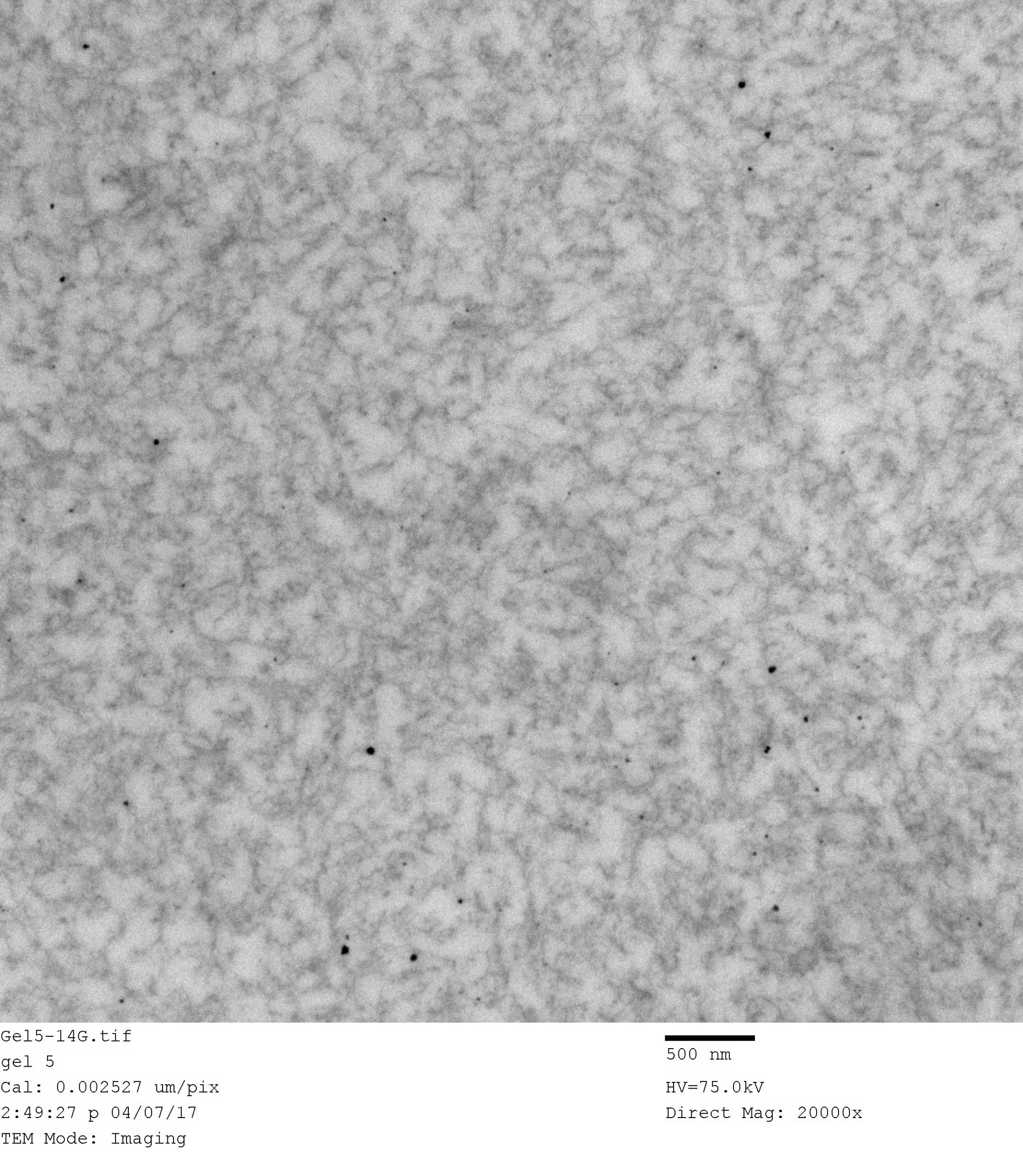

Supplement: Supplementary file 5 — Source Data [file 41467_2022_30980_MOESM5_ESM.zip › TEM images of fibrin gel before and after confinement/Before confinement.jpeg]

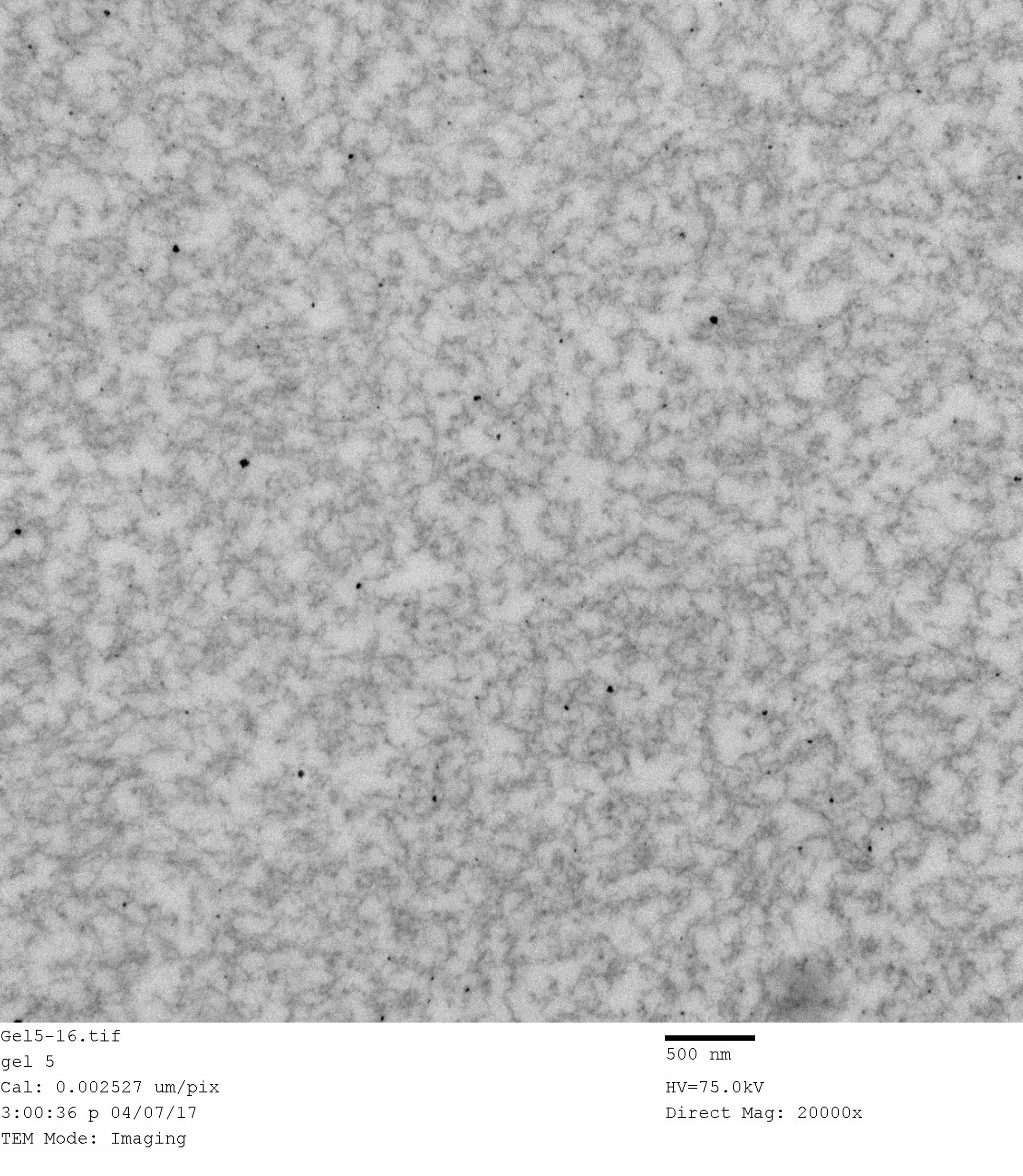

Supplement: Supplementary file 5 — Source Data [file 41467_2022_30980_MOESM5_ESM.zip › TEM images of fibrin gel before and after confinement/Before confinement_2.jpeg]

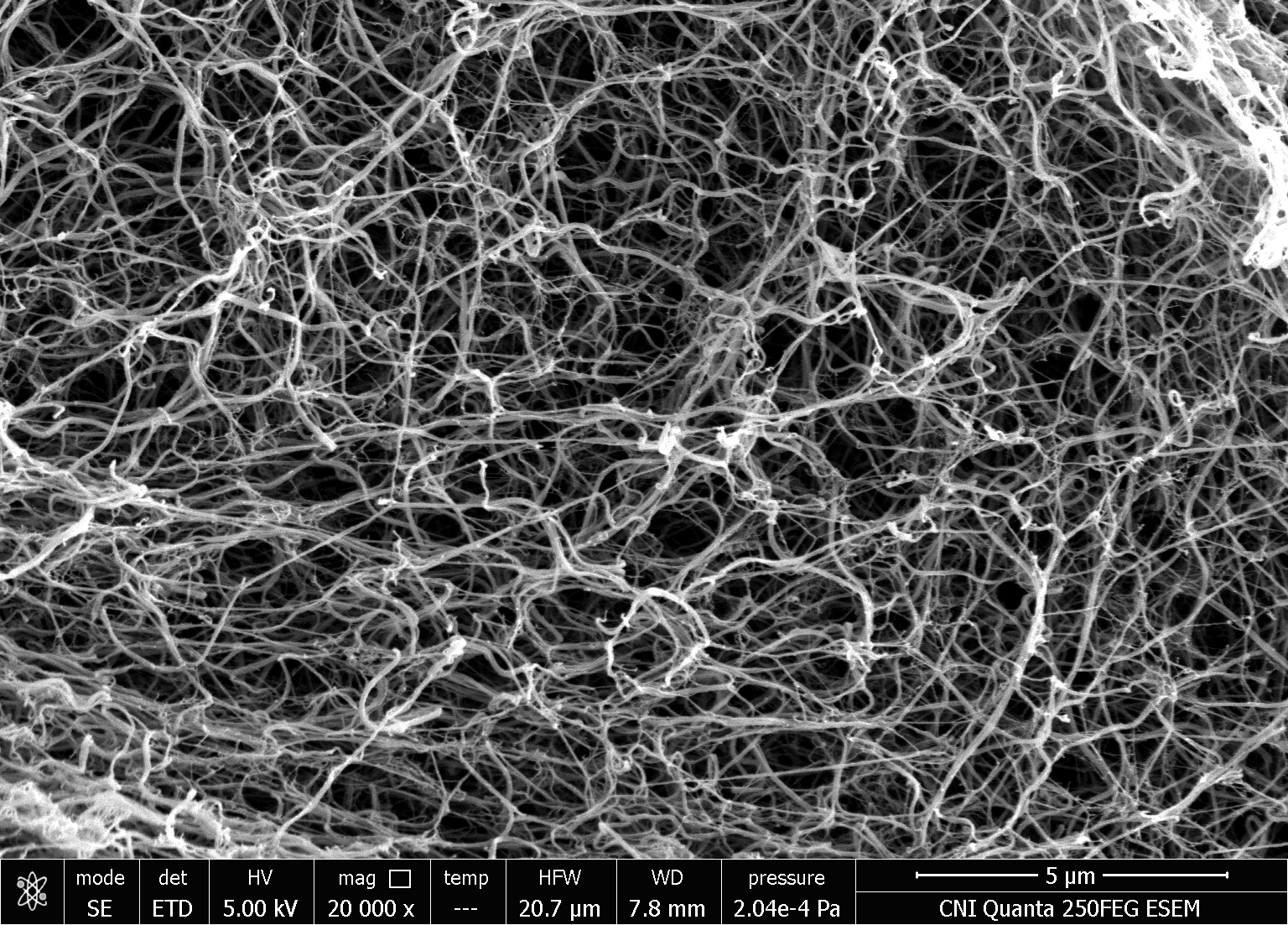

Supplement: Supplementary file 5 — Source Data [file 41467_2022_30980_MOESM5_ESM.zip › SEM images of fibrin gel/MM/3-1_006.tif]

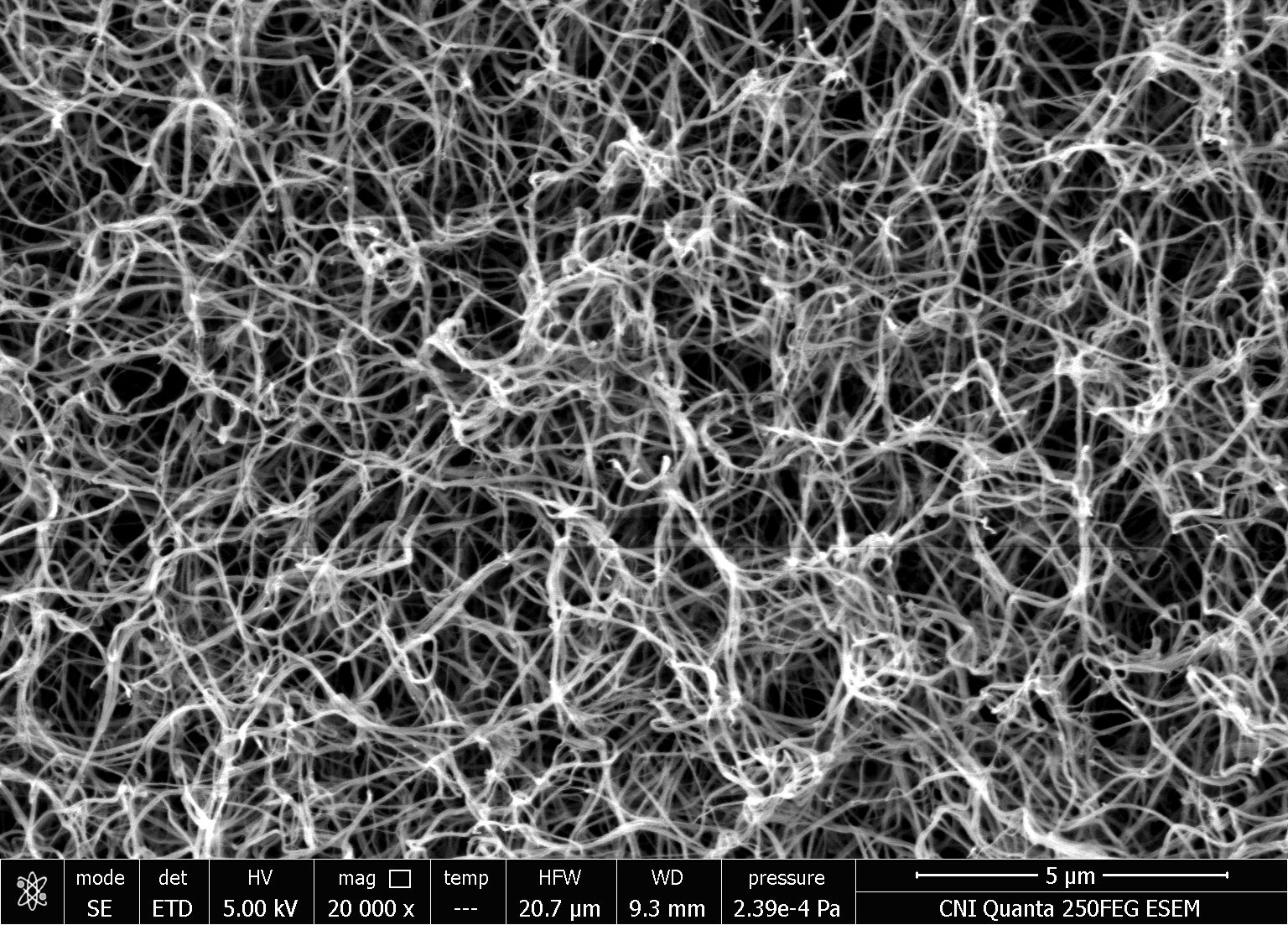

Supplement: Supplementary file 5 — Source Data [file 41467_2022_30980_MOESM5_ESM.zip › SEM images of fibrin gel/MM/3-1_014.jpg]

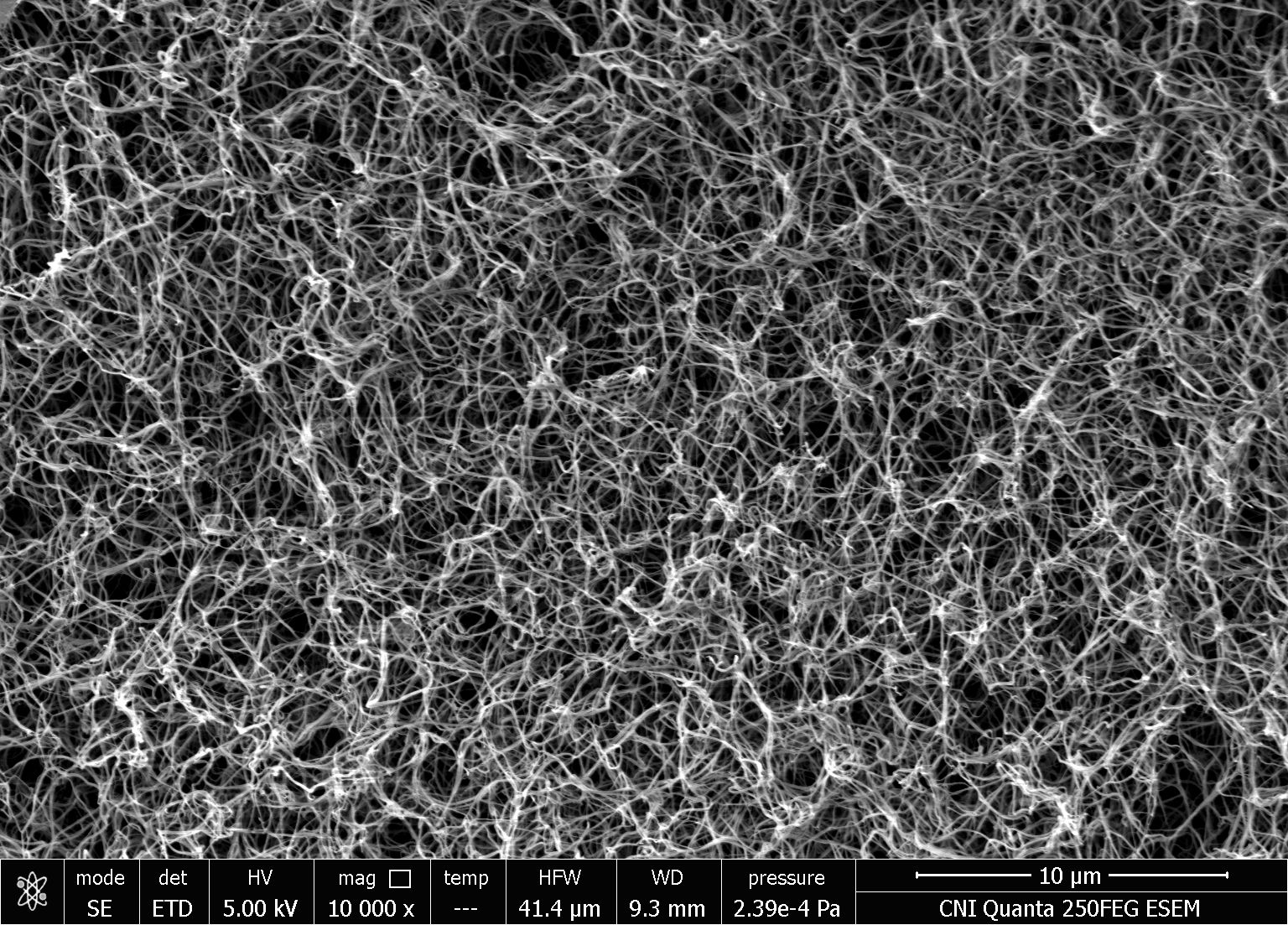

Supplement: Supplementary file 5 — Source Data [file 41467_2022_30980_MOESM5_ESM.zip › SEM images of fibrin gel/MM/3-1_015.jpg]

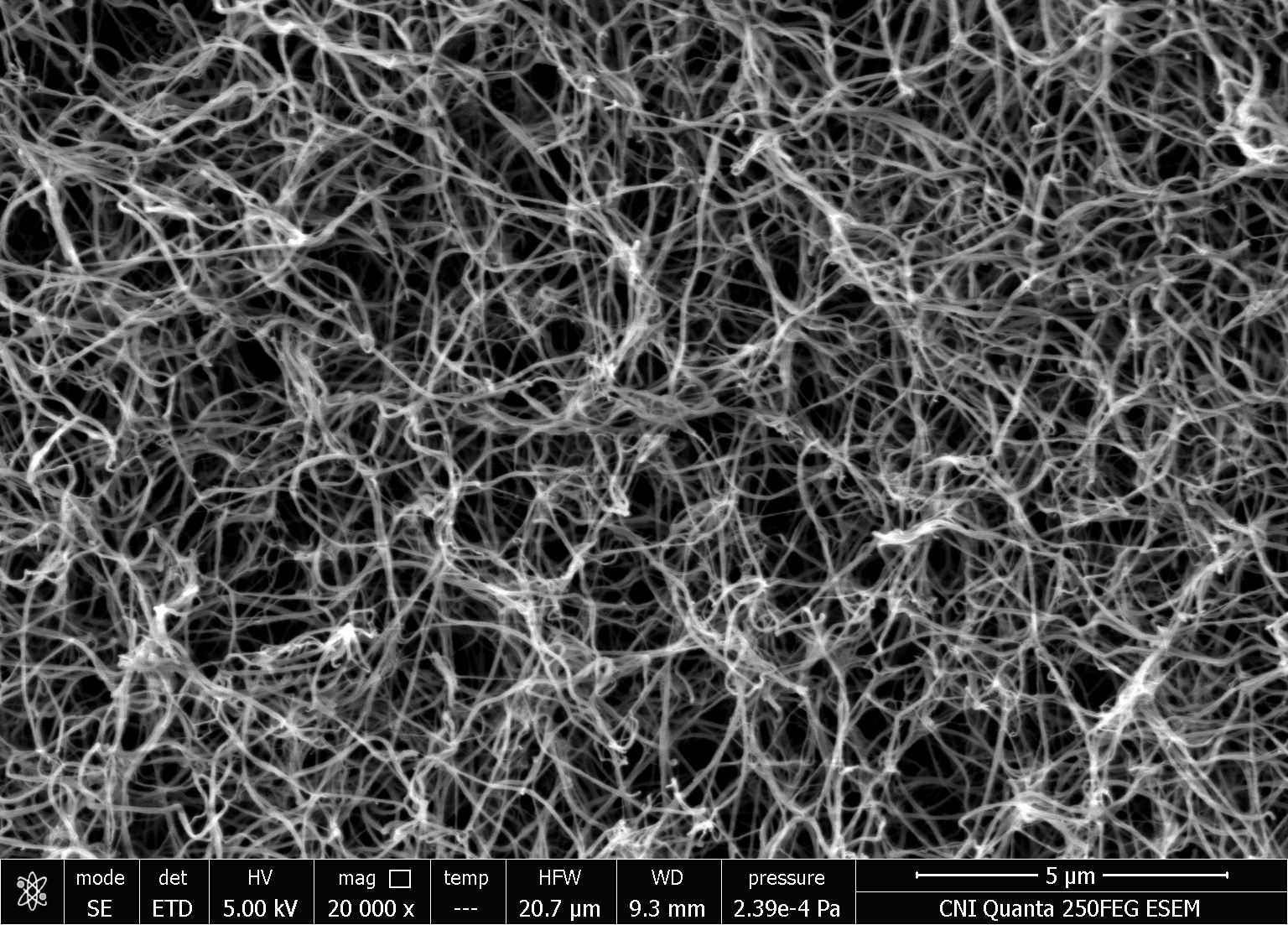

Supplement: Supplementary file 5 — Source Data [file 41467_2022_30980_MOESM5_ESM.zip › SEM images of fibrin gel/MM/3-1_016.jpg]

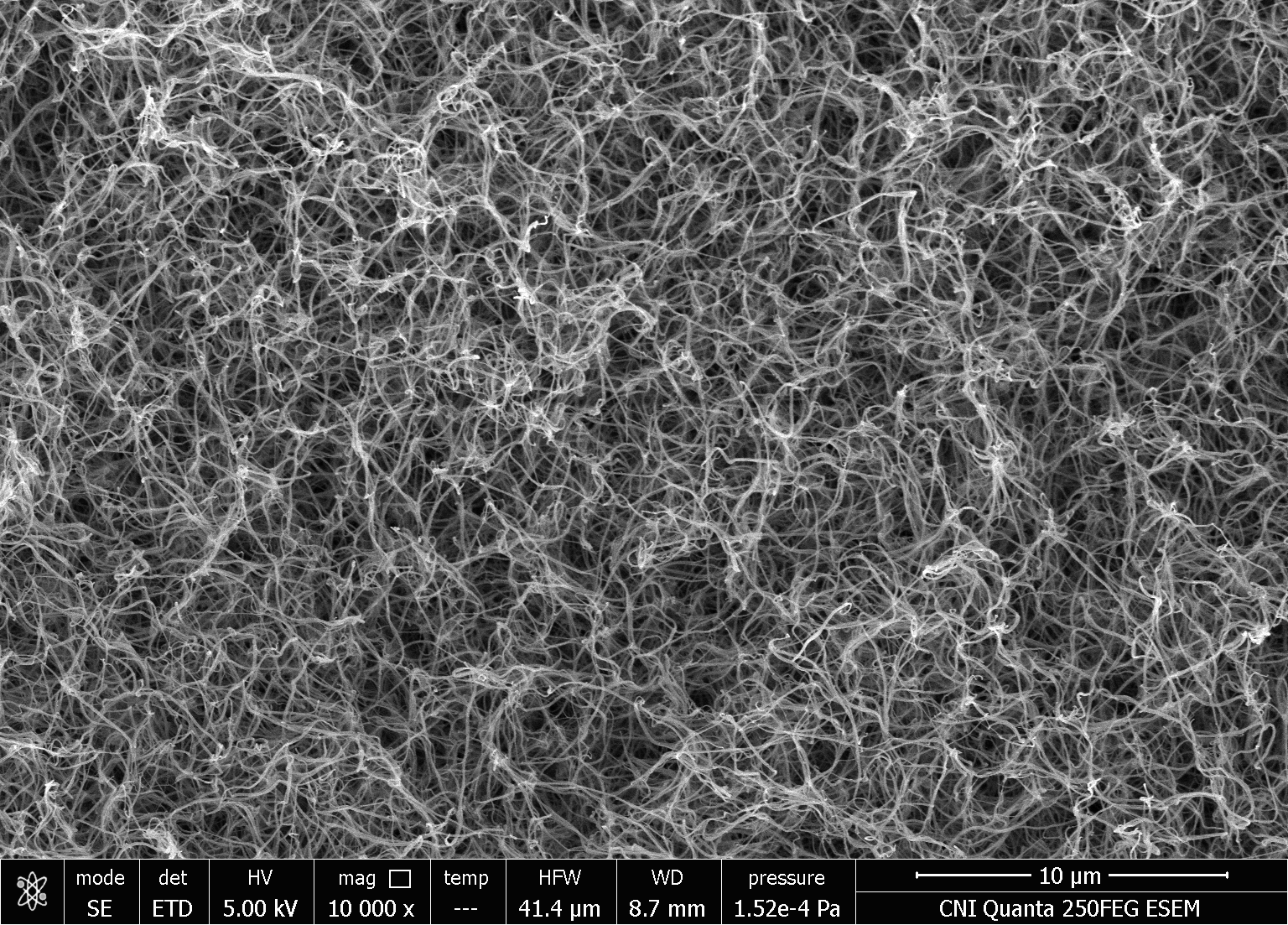

Supplement: Supplementary file 5 — Source Data [file 41467_2022_30980_MOESM5_ESM.zip › SEM images of fibrin gel/MM/3-3_002.tif]

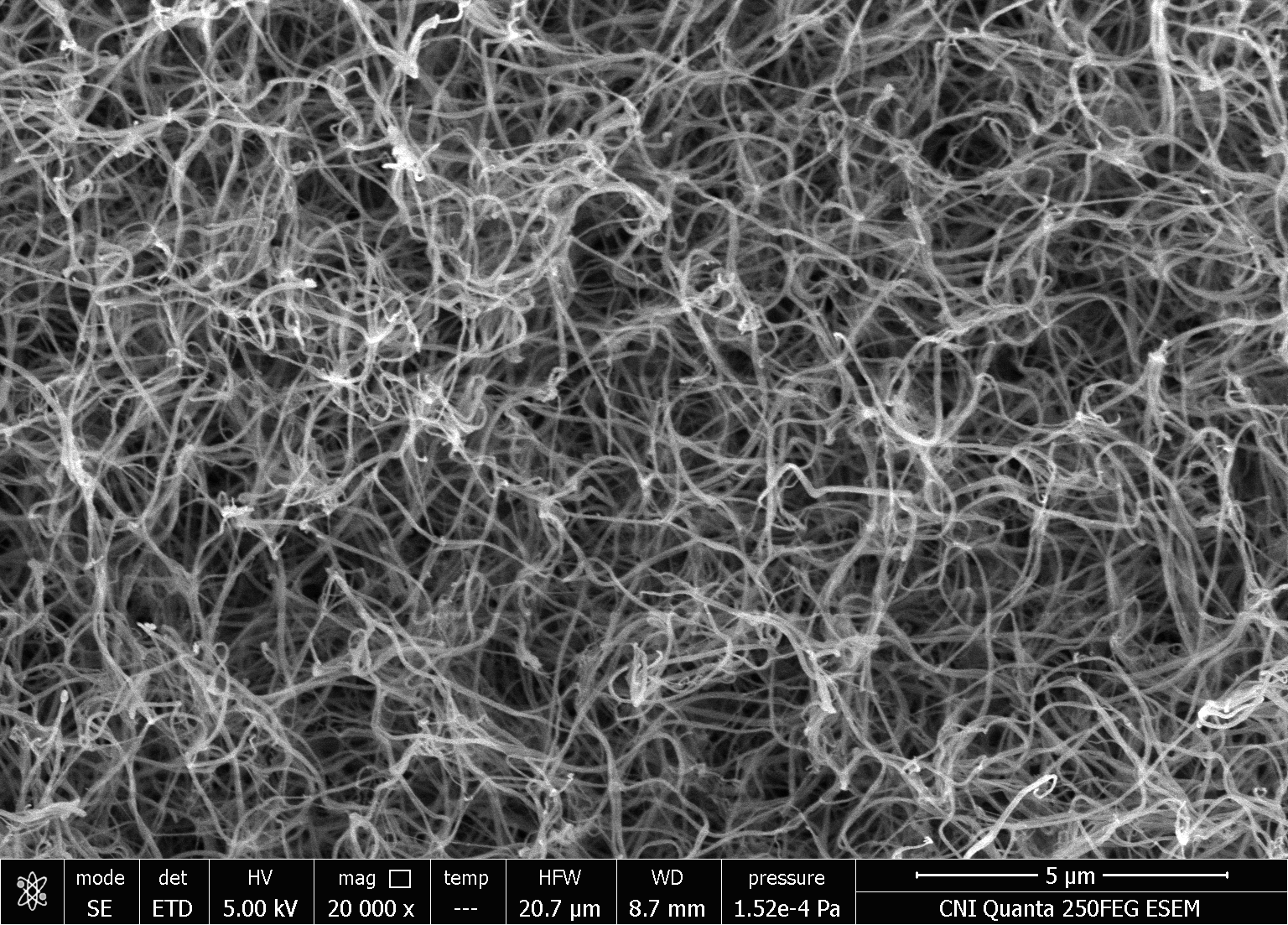

Supplement: Supplementary file 5 — Source Data [file 41467_2022_30980_MOESM5_ESM.zip › SEM images of fibrin gel/MM/3-3_003.tif]

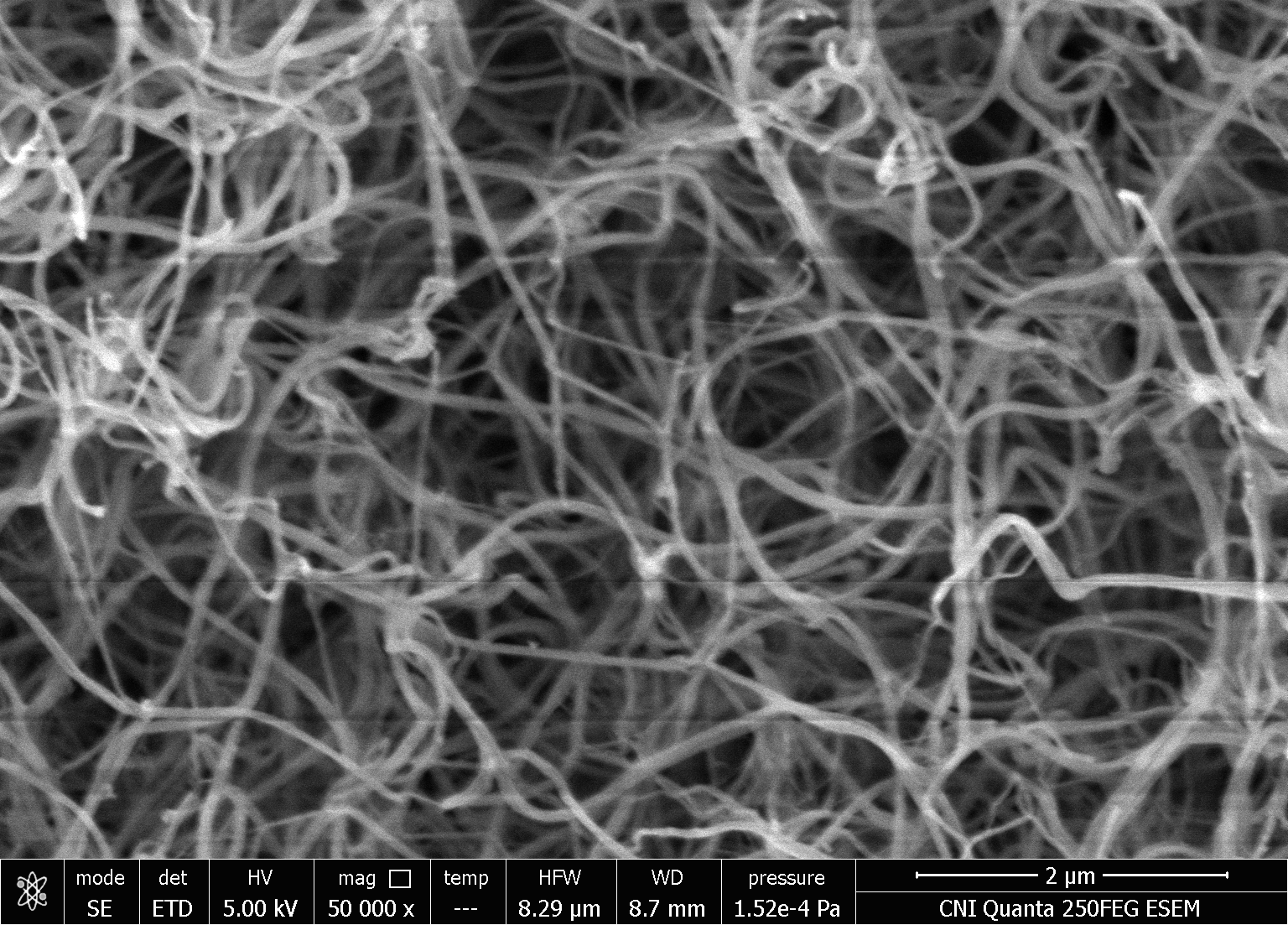

Supplement: Supplementary file 5 — Source Data [file 41467_2022_30980_MOESM5_ESM.zip › SEM images of fibrin gel/MM/3-3_004.tif]

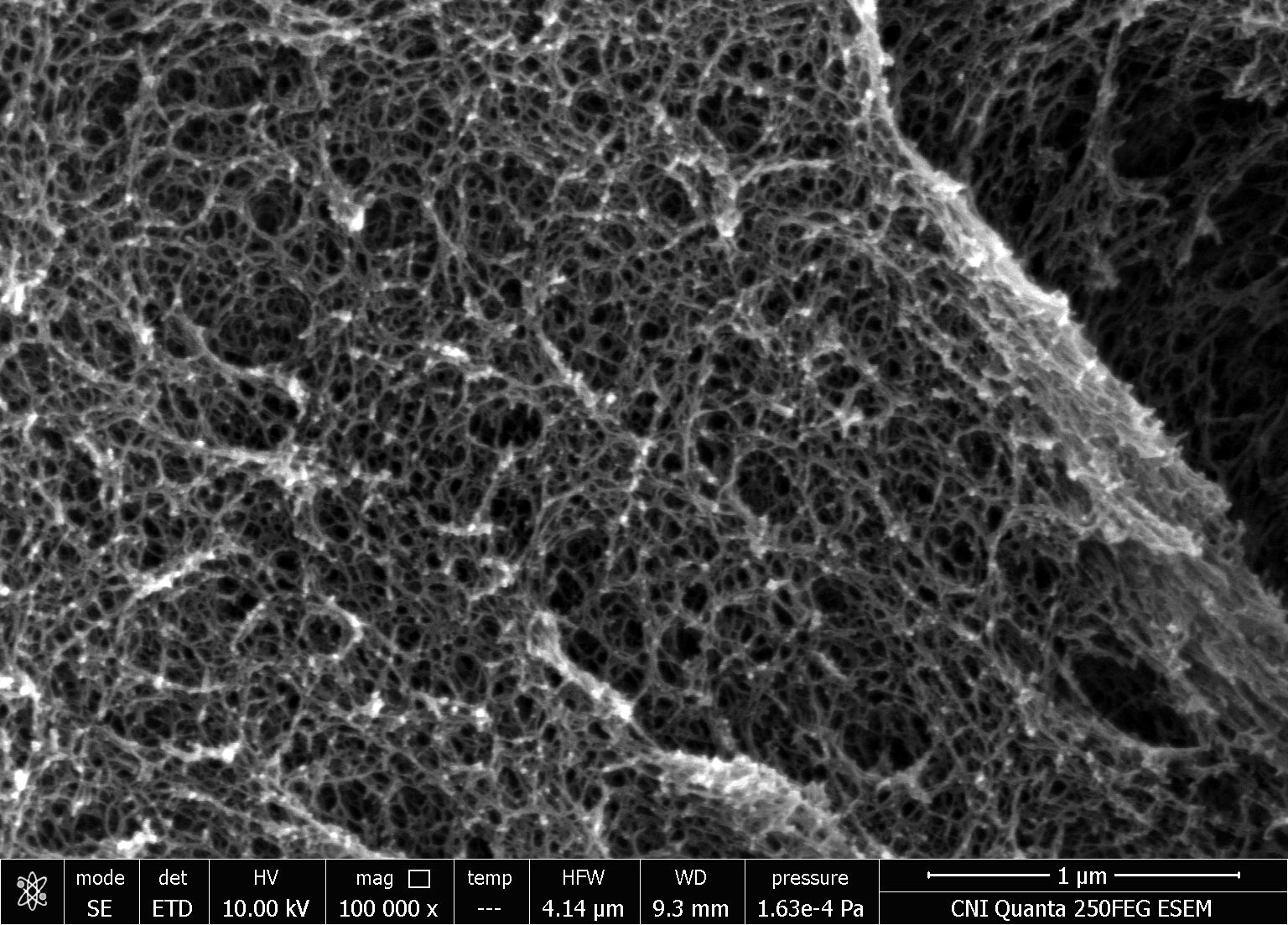

Supplement: Supplementary file 5 — Source Data [file 41467_2022_30980_MOESM5_ESM.zip › SEM images of fibrin gel/RM/5-1_005.jpg]

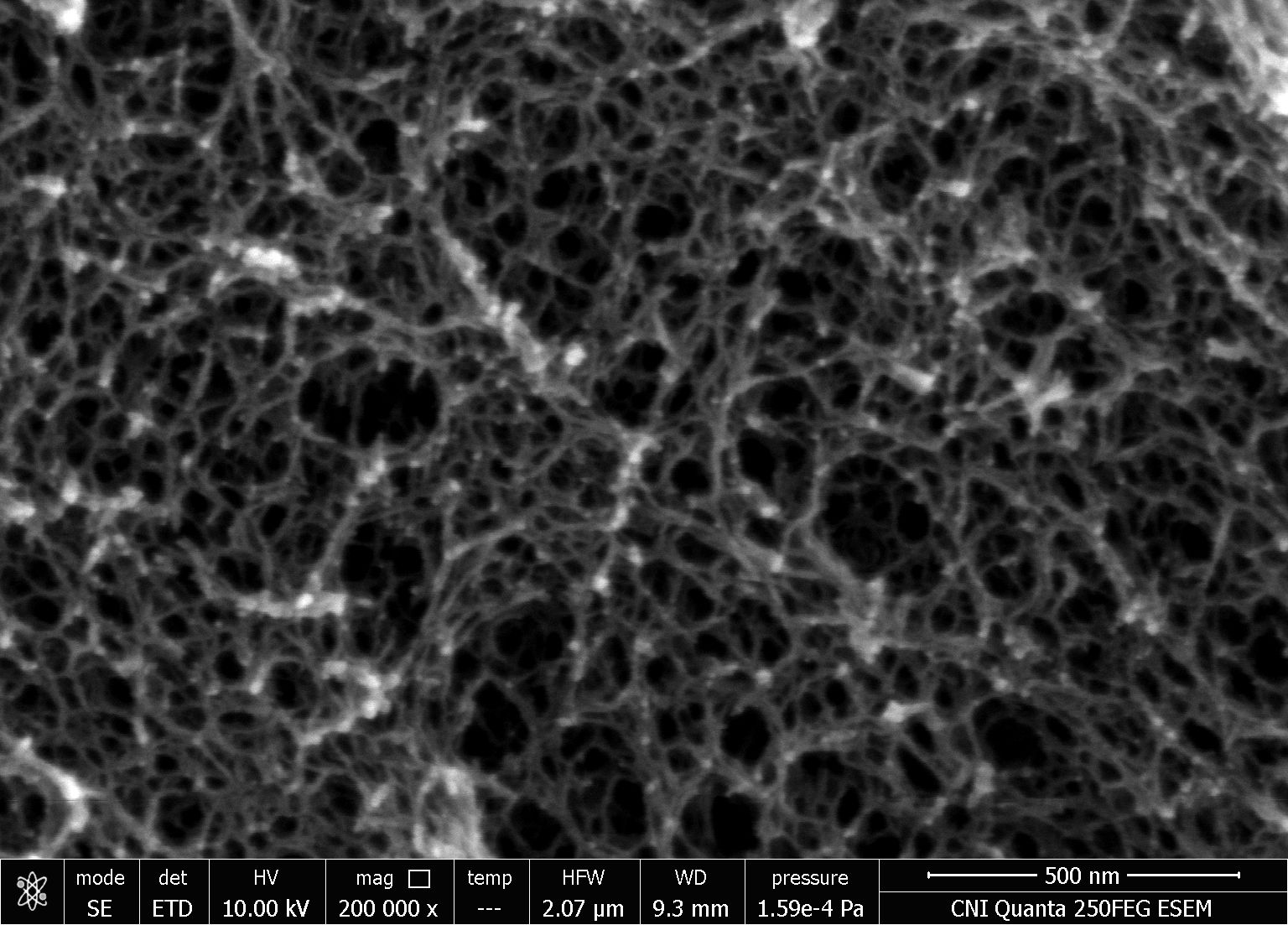

Supplement: Supplementary file 5 — Source Data [file 41467_2022_30980_MOESM5_ESM.zip › SEM images of fibrin gel/RM/5-1_006.jpg]

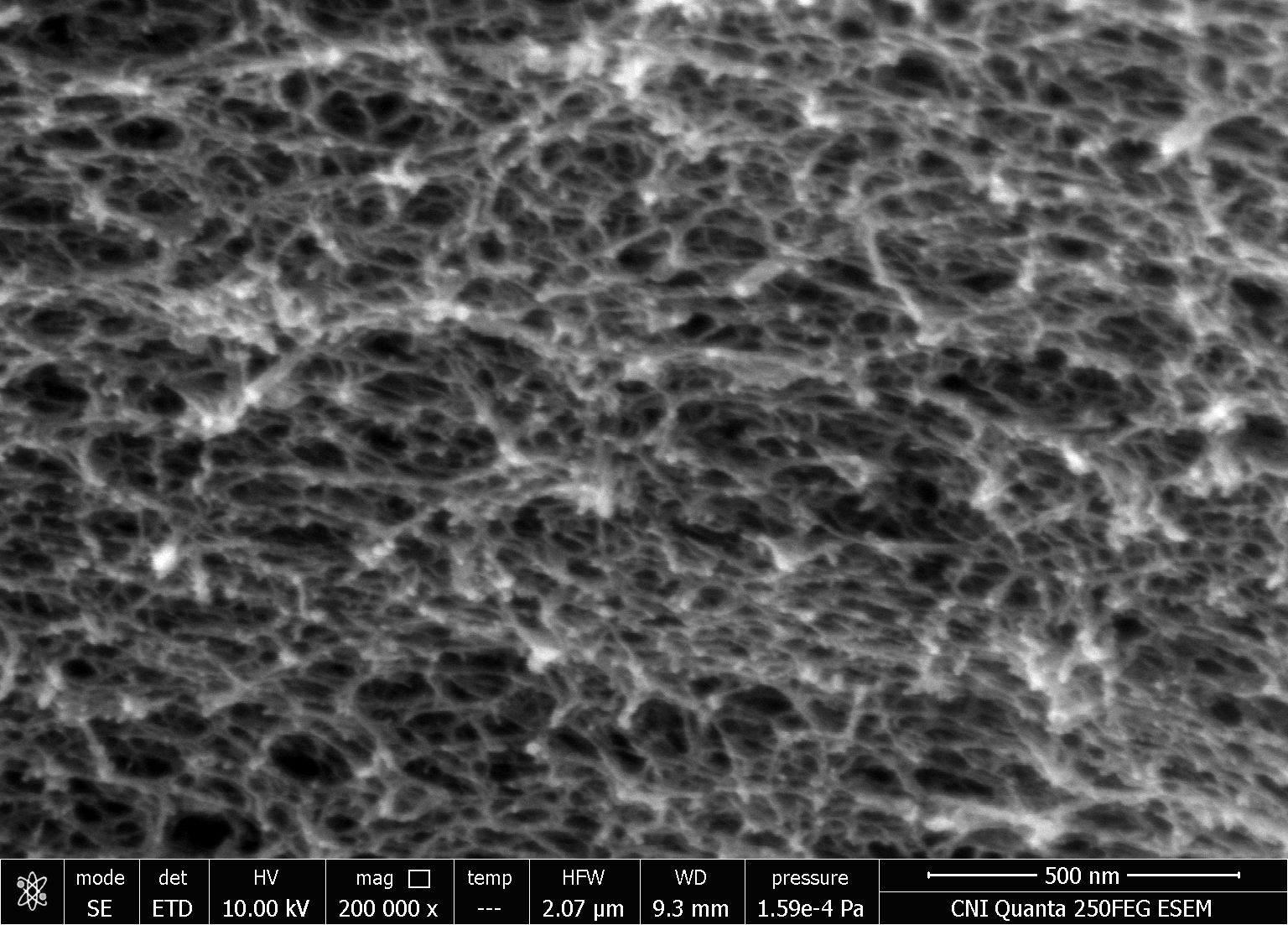

Supplement: Supplementary file 5 — Source Data [file 41467_2022_30980_MOESM5_ESM.zip › SEM images of fibrin gel/RM/5-1_007.jpg]

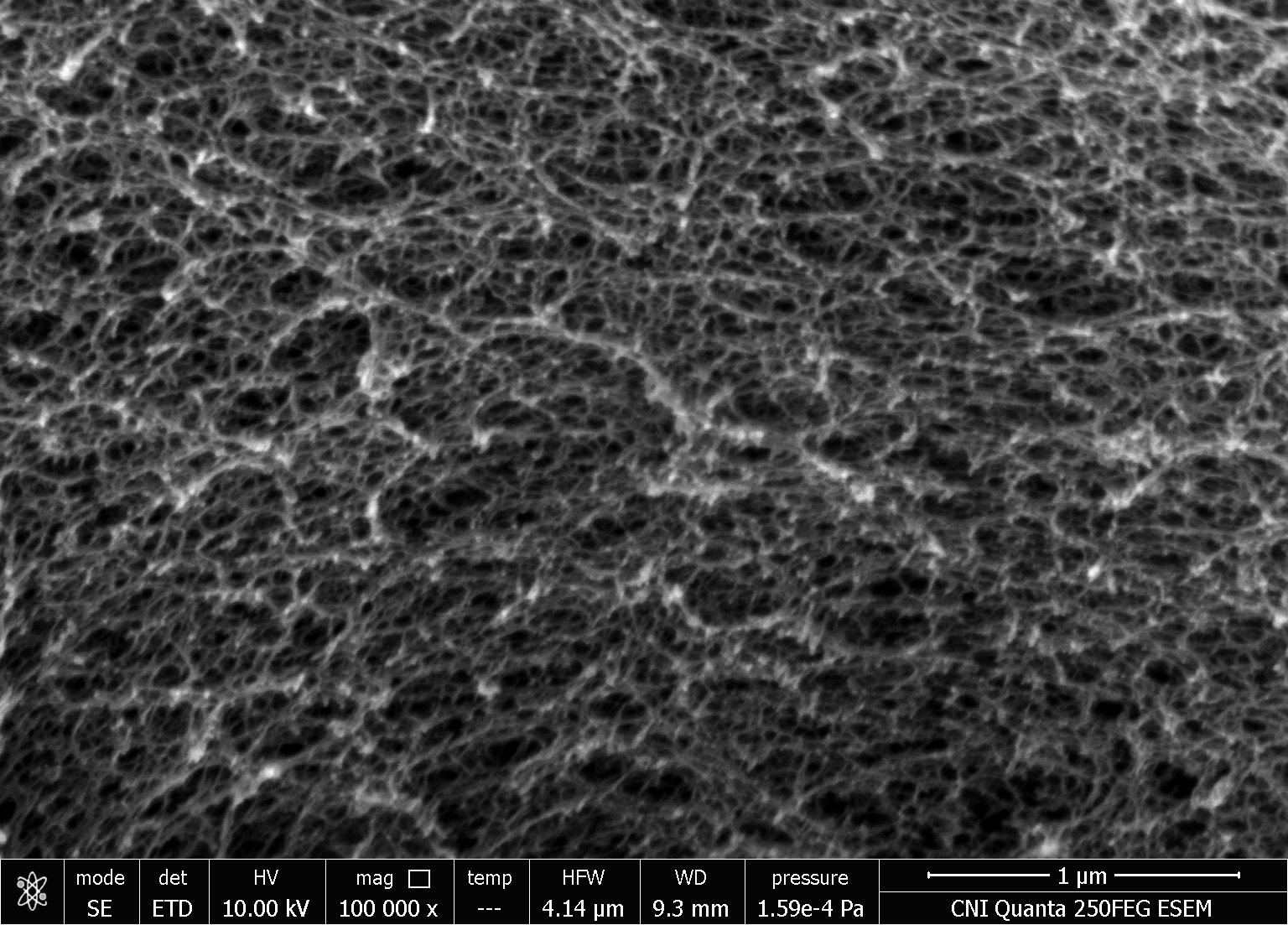

Supplement: Supplementary file 5 — Source Data [file 41467_2022_30980_MOESM5_ESM.zip › SEM images of fibrin gel/RM/5-1_008.jpg]

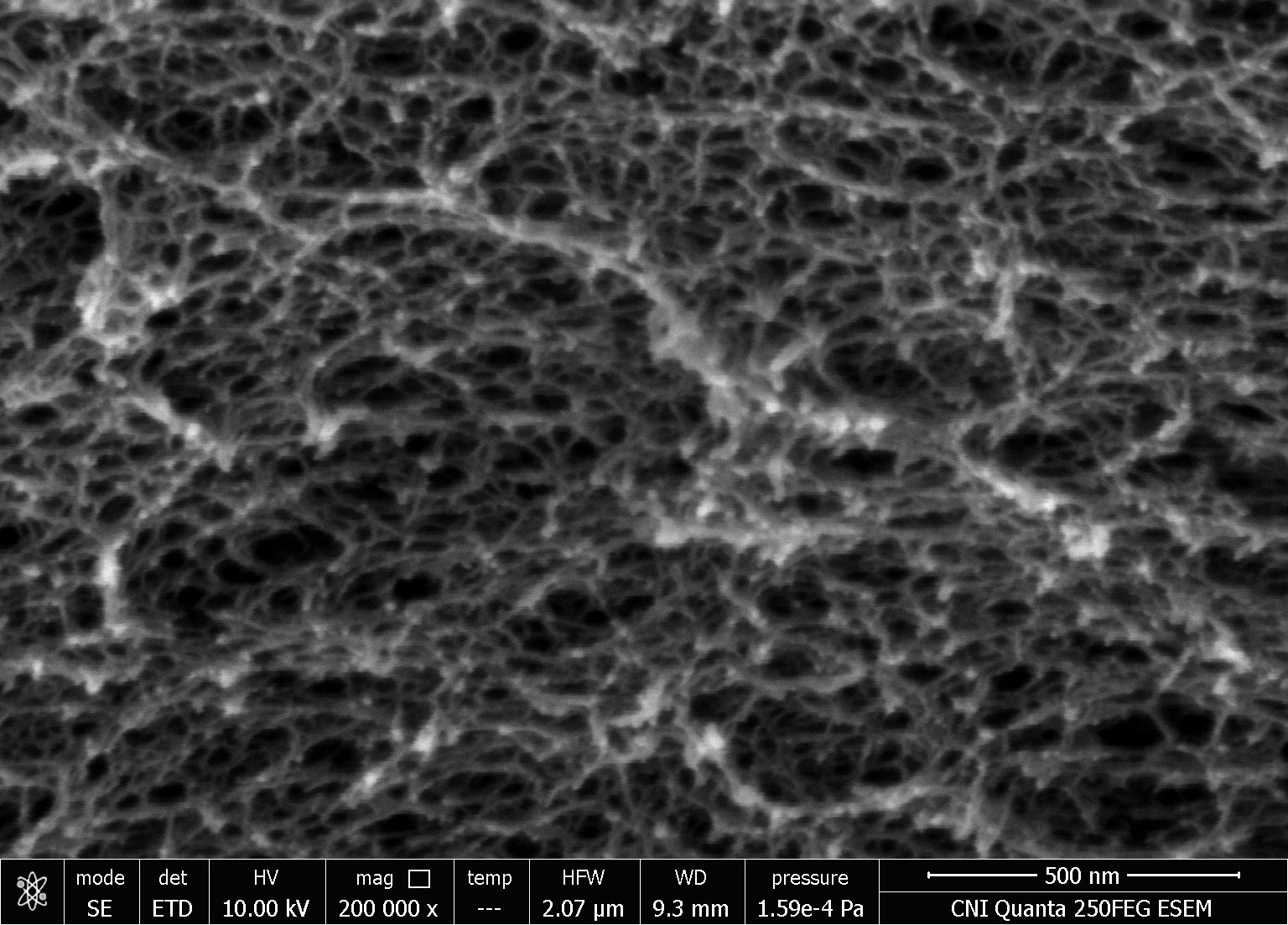

Supplement: Supplementary file 5 — Source Data [file 41467_2022_30980_MOESM5_ESM.zip › SEM images of fibrin gel/RM/5-1_009.jpg]

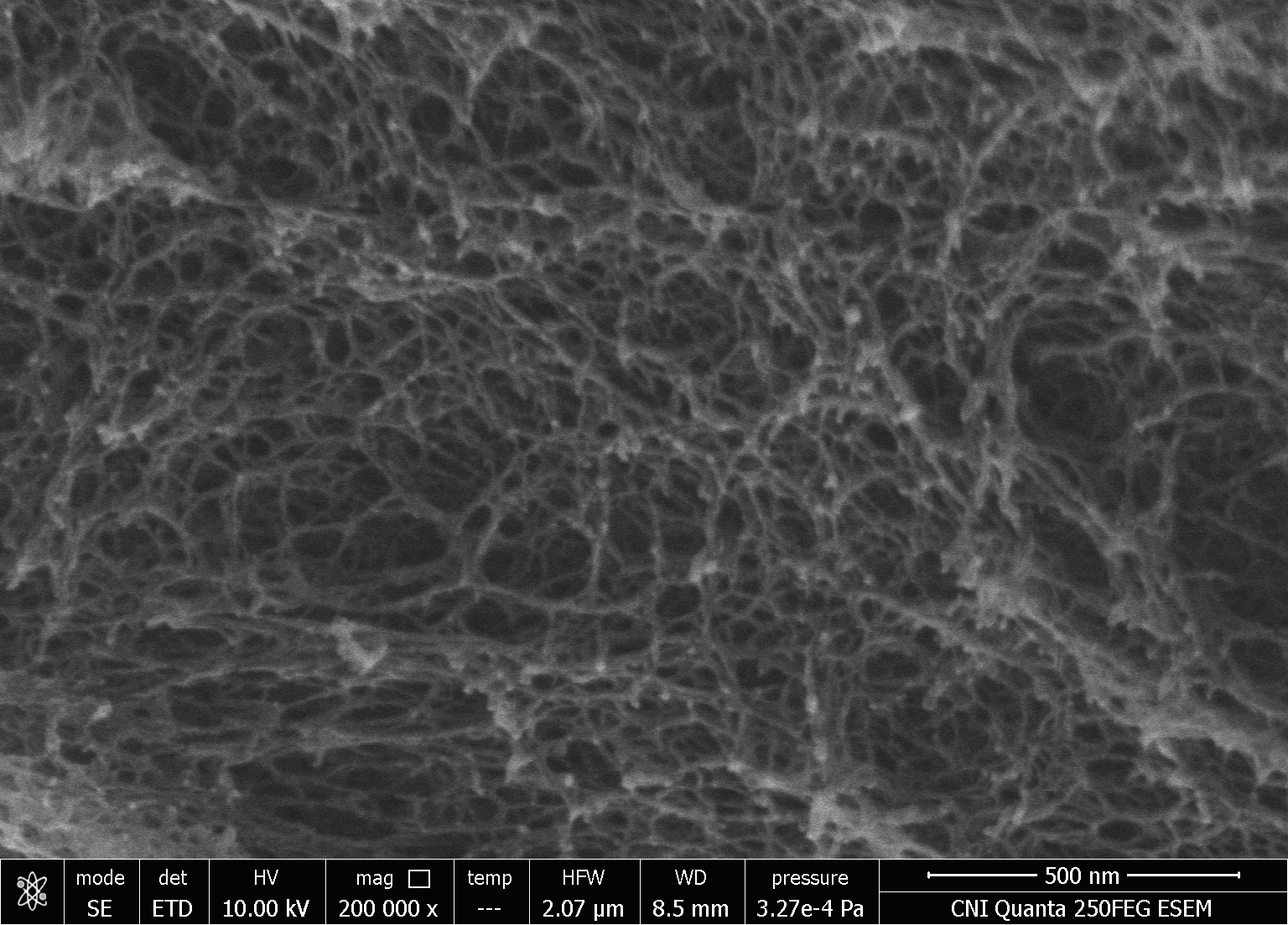

Supplement: Supplementary file 5 — Source Data [file 41467_2022_30980_MOESM5_ESM.zip › SEM images of fibrin gel/RM/5-2_009.tif]

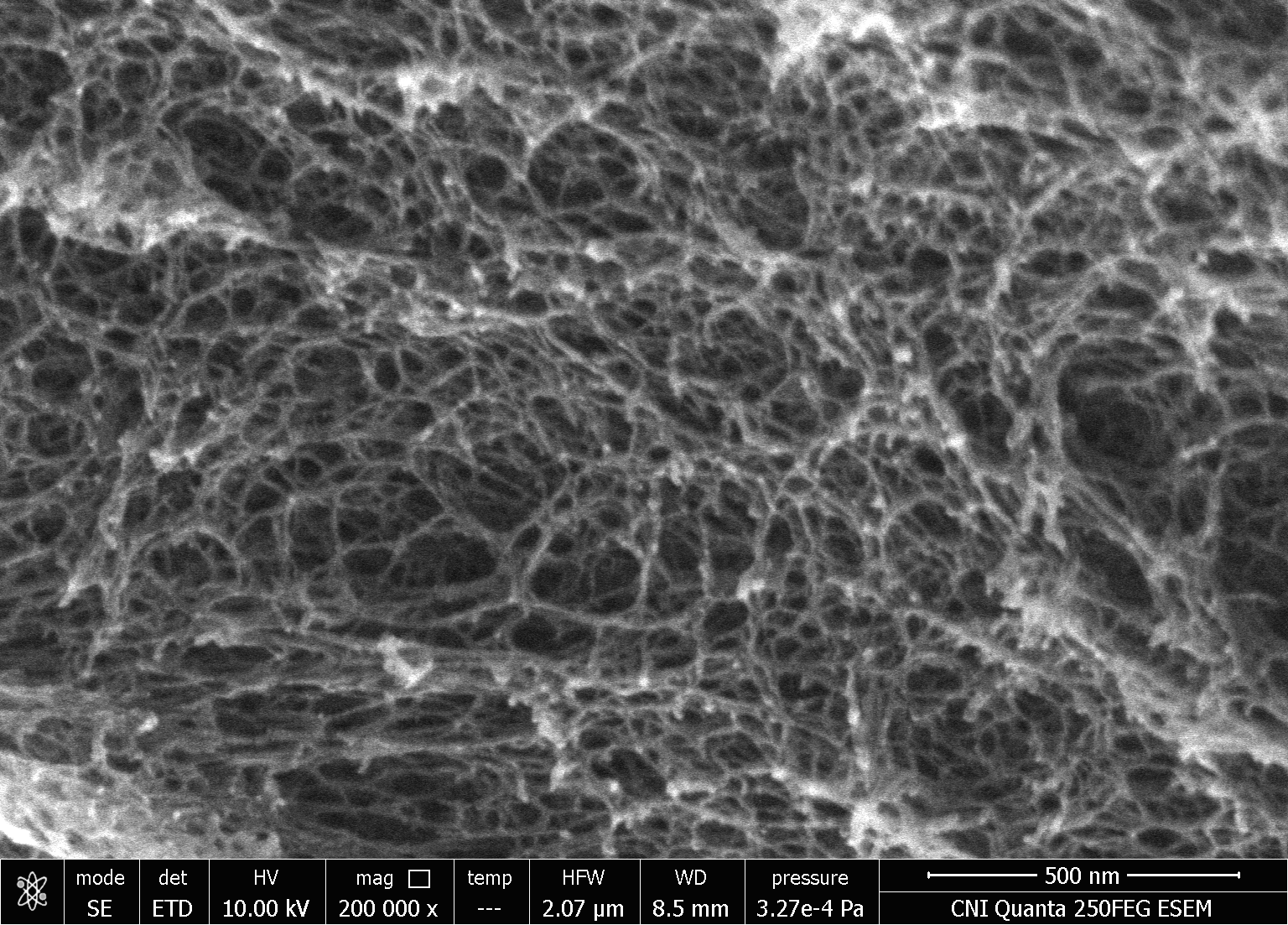

Supplement: Supplementary file 5 — Source Data [file 41467_2022_30980_MOESM5_ESM.zip › SEM images of fibrin gel/RM/5-2_010.tif]

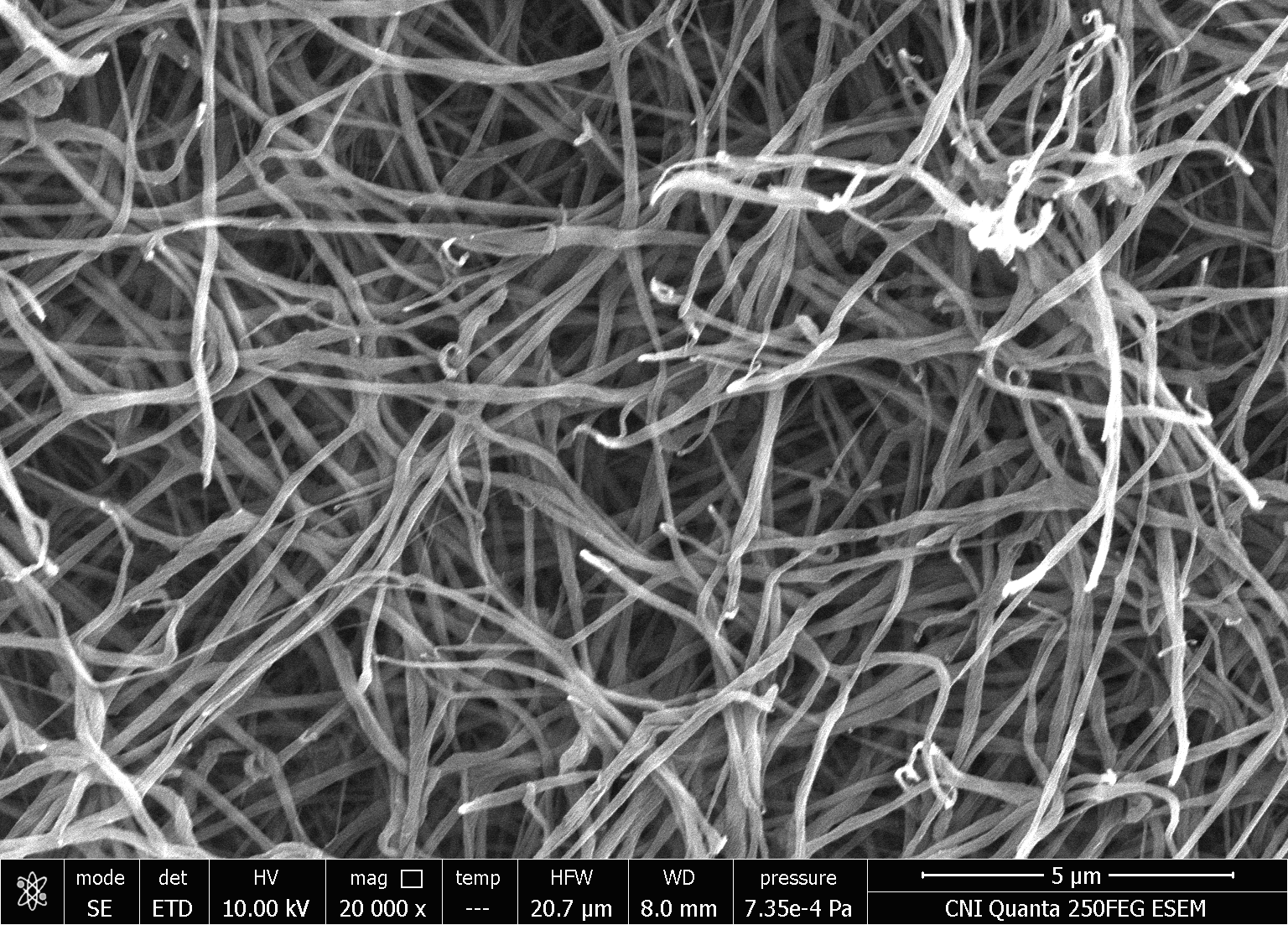

Supplement: Supplementary file 5 — Source Data [file 41467_2022_30980_MOESM5_ESM.zip › SEM images of fibrin gel/SM/1-2_004.tif]

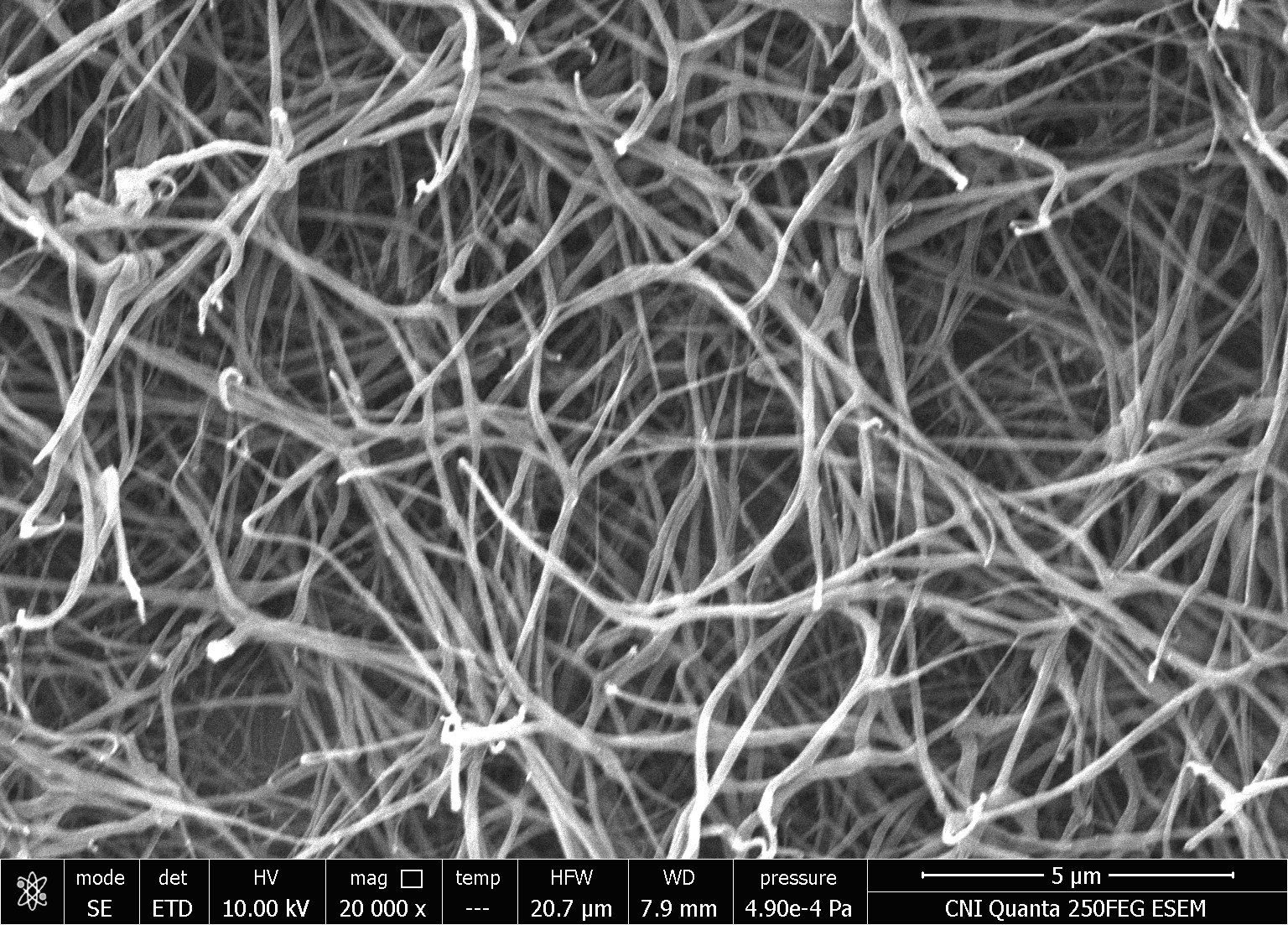

Supplement: Supplementary file 5 — Source Data [file 41467_2022_30980_MOESM5_ESM.zip › SEM images of fibrin gel/SM/1-4 5_002.tif]

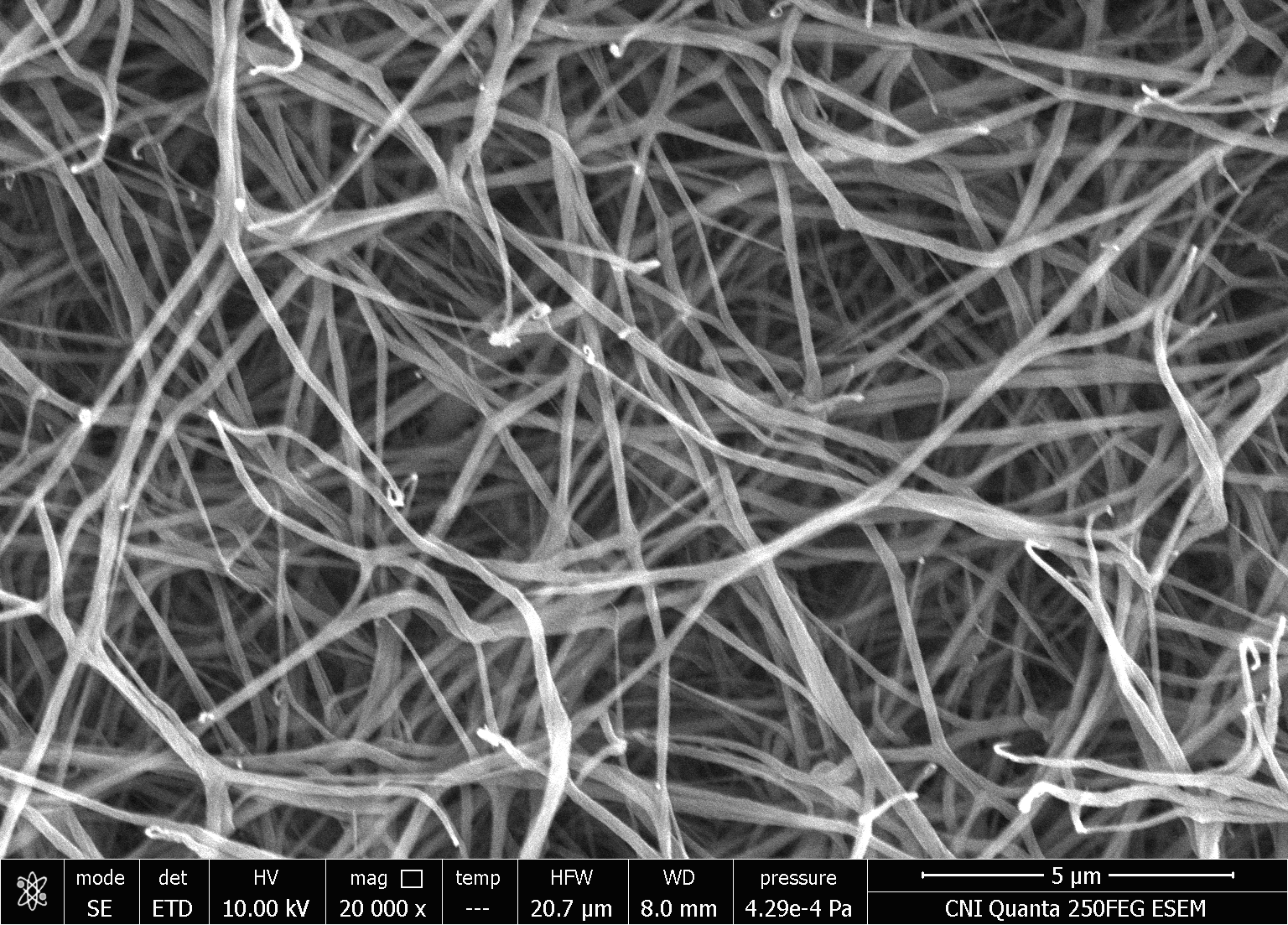

Supplement: Supplementary file 5 — Source Data [file 41467_2022_30980_MOESM5_ESM.zip › SEM images of fibrin gel/SM/1-6_003.tif]

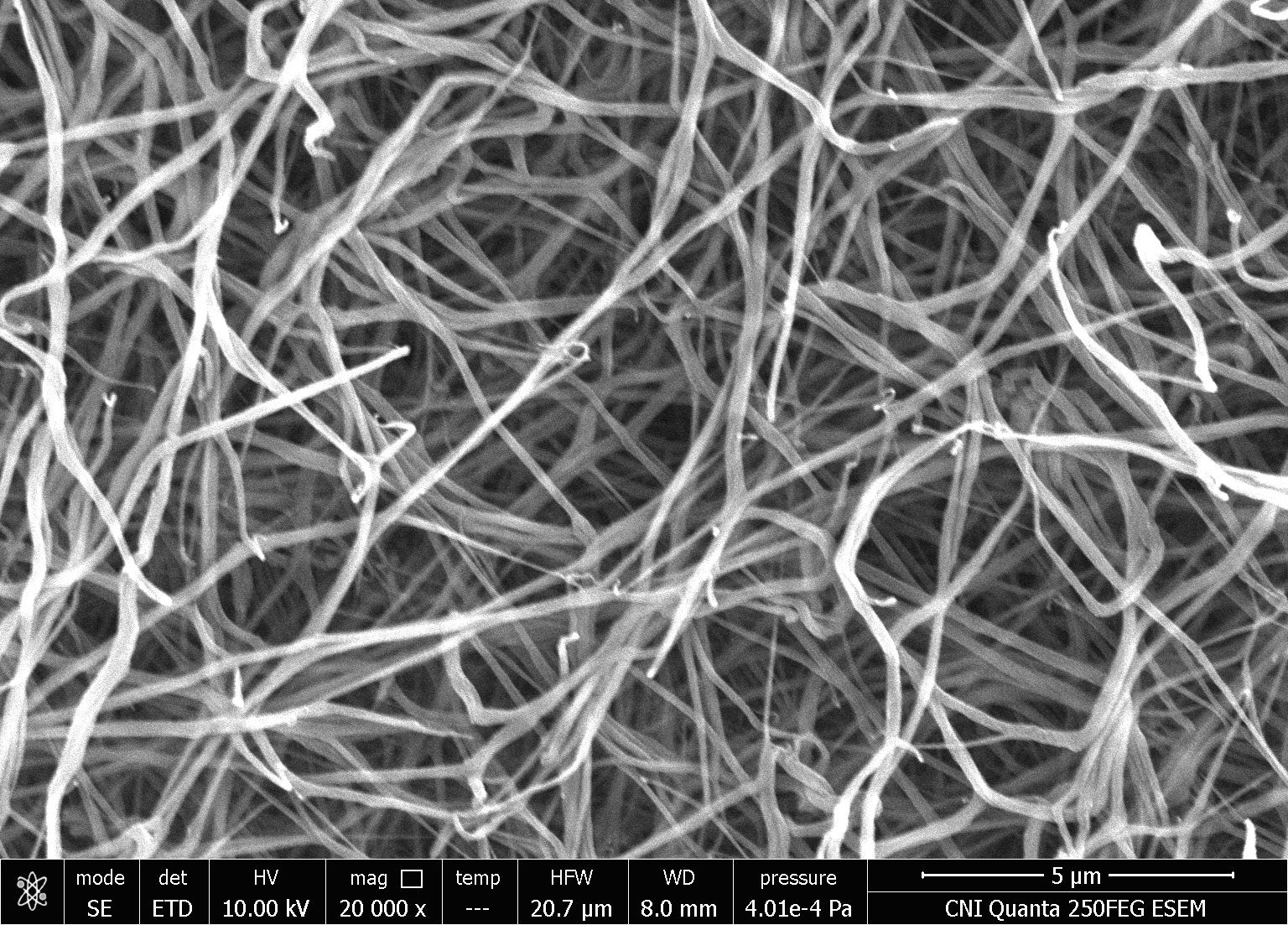

Supplement: Supplementary file 5 — Source Data [file 41467_2022_30980_MOESM5_ESM.zip › SEM images of fibrin gel/SM/1-6_005.tif]

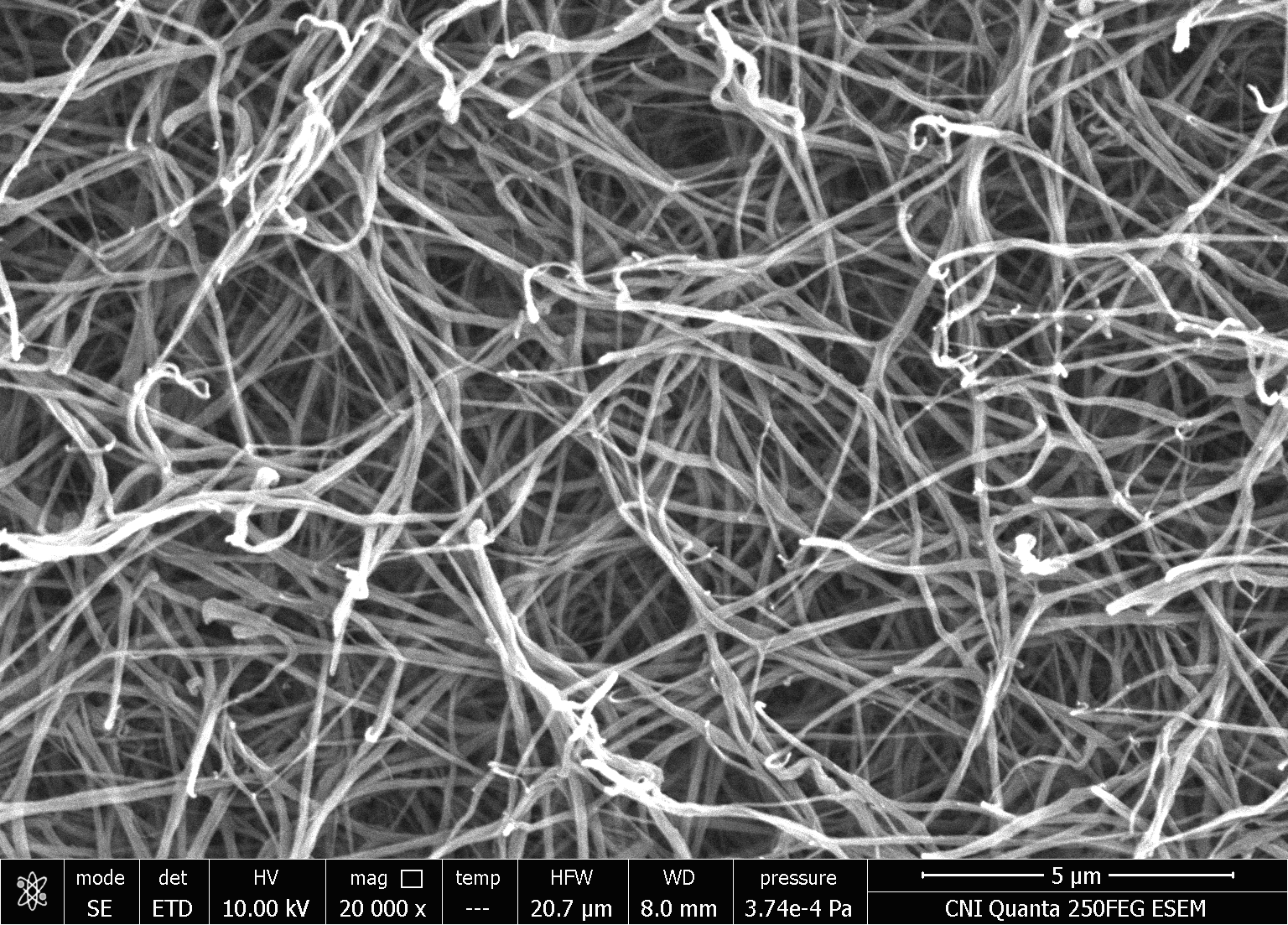

Supplement: Supplementary file 5 — Source Data [file 41467_2022_30980_MOESM5_ESM.zip › SEM images of fibrin gel/SM/1-8_001.tif]
